# The functional decline of tomato plants infected by *Candidatus* Liberbacter solanacearum: an RNA-seq transcriptomic analysis

**DOI:** 10.3389/fpls.2024.1325254

**Published:** 2024-02-01

**Authors:** Jiacheng Chuan, Jingbai Nie, William Rodney Cooper, Wen Chen, Lawrence Hale, Xiang Li

**Affiliations:** ^1^ Charlottetown Laboratory, Canadian Food Inspection Agency, Charlottetown, PE, Canada; ^2^ Biology Department, University of Prince Edward Island, Charlottetown, PE, Canada; ^3^ Temperate Tree Fruit and Vegetable Research Unit, USDA-ARS, Wapato, WA, United States; ^4^ Ottawa Research and Development Centre, Agriculture and Agri-Food Canada, Ottawa, ON, Canada

**Keywords:** *Candidatus* Liberibacter solanacearum, zebra chip, transcriptomics, gene expression, bioinformatics

## Abstract

**Introduction:**

*Candidatus* Liberibacter solanacearum (*C*Lso) is a regulated plant pathogen in European and some Asian countries, associated with severe diseases in economically important Apiaceous and Solanaceous crops, including potato, tomato, and carrot. Eleven haplotypes of *C*Lso have been identified based on the difference in rRNA and conserved genes and host and pathogenicity. Although it is pathogenic to a wide range of plants, the mechanisms of plant response and functional decline of host plants are not well defined. This study aims to describe the underlying mechanism of the functional decline of tomato plants infected by *C*Lso by analyzing the transcriptomic response of tomato plants to *C*Lso haplotypes A and B.

**Methods:**

Next-generation sequencing (NGS) data were generated from total RNA of tomato plants infected by *C*Lso haplotypes A and B, and uninfected tomato plants, while qPCR analysis was used to validate the *in-silico* expression analysis. Gene Ontology and KEGG pathways were enriched using differentially expressed genes.

**Results:**

Plants infected with *C*Lso haplotype B saw 229 genes upregulated when compared to uninfected plants, while 1,135 were downregulated. Healthy tomato plants and plants infected by haplotype A had similar expression levels, which is consistent with the fact that *C*Lso haplotype A does not show apparent symptoms in tomato plants. Photosynthesis and starch biosynthesis were impaired while starch amylolysis was promoted in plants infected by *C*Lso haplotype B compared with uninfected plants. The changes in pathway gene expression suggest that carbohydrate consumption in infected plants was more extensive than accumulation. In addition, cell-wall-related genes, including steroid biosynthesis pathways, were downregulated in plants infected with *C*Lso haplotype B suggesting a reduction in membrane fluidity, cell signaling, and defense against bacteria. In addition, genes in phenylpropanoid metabolism and DNA replication were generally suppressed by *C*Lso infection, affecting plant growth and defense.

**Discussion:**

This study provides insights into plants’ defense and functional decline due to pathogenic *C*Lso using whole transcriptome sequencing and qPCR validation. Our results show how tomato plants react in metabolic pathways during the deterioration caused by pathogenic *C*Lso. Understanding the underlying mechanisms can enhance disease control and create opportunities for breeding resistant or tolerant varieties.

## 1 Introduction

*Candidatus* Liberibacter solanacearum (*C*Lso) is a phloem-limited pathogen associated with diseases in many Apiaceous and Solanaceous plants, including potato, tomato, carrot, pepper, eggplant, celery, and leek (Hansen et al., 2008; Liefting et al., 2008; Sumner-Kalkun et al., 2020). This pathogen is associated with foliar dieback in susceptible plants, and causes zebra chip disease of potato (Li et al., 2013). The *C*Lso has a wide range of psyllid vectors, including *Bactericera cockerelli*, *B. trigonica*, and *Trioza apicalis* (Munyaneza et al., 2007b; Sumner-Kalkun et al., 2020). Also, it has been suggested that non-solanaceous psyllids might be the potential vectors of *C*Lso, and therefore more plant hosts might carry the bacteria (Borges et al., 2017; Swisher Grimm and Garczynski, 2019).

*C*Lso is categorized into haplotypes based on single nucleotide polymorphisms (SNPs) in ribosomal RNA and housekeeping genes. Different haplotypes vary in pathogenicity to different host plants (Mendoza-Herrera et al., 2018; Swisher Grimm et al., 2018; Harrison et al., 2019; Harrison et al., 2020; Sumner-Kalkun et al., 2020). Until now, 15 haplotypes of *C*Lso have been identified around the world, including A, B (Wen et al., 2009), C (Munyaneza et al., 2010b), D (Nelson et al., 2013), E (Teresani et al., 2014), F (Swisher Grimm and Garczynski, 2019), G (Mauck et al., 2019), H (Haapalainen et al., 2020), H(Con) (Contreras-Rendon et al., 2020), U (Haapalainen et al., 2018), Cras1, Cras2 (Sumner-Kalkun et al., 2020), Aph1, Aph2, and Aph3 (Grimm et al., 2022). Haplotypes A, B and F are pathogens of ZC in the United States, with haplotypes A and B also occurring in Mexico and New Zealand (Hansen et al., 2008; Liefting et al., 2009; Munyaneza et al., 2009; Antolinez et al., 2017; Haapalainen et al., 2020). Haplotype C was found in carrots in Finland, Sweden and Norway (Nelson et al., 2013). Haplotype D was found in carrots and celeries in Spain, the Canary Islands, Southern Europe and Morocco (Nelson et al., 2013; Alfaro-Fernández et al., 2017). Haplotype E was found in Southern Europe, including Spain and Morocco, in carrot and celery (Alfaro-Fernández et al., 2017; Ben Othmen et al., 2018). Haplotype G was discovered and recovered in herbarium specimens of wild species (*Solanum elaeagnifolium, S. americanum*, and *S. umbelliferum*), indicating that *C*Lso has been in South America since at least 1970 (Mauck et al., 2019). Haplotype U was found in *Urtica dioica* in Finland (Haapalainen et al., 2018). Cras1 and Cras2 were found in psyllid vectors in Scotland (Sumner-Kalkun et al., 2020). Aph1, Aph2 and Aph3 were identified in psyllid vectors collected from yellow sticky cards near potato farms in southern Oregon of the United States; the impact of Aph1 to Aph3 on crops is not known (Grimm et al., 2022)

In general, *C*Lso is injected into the phloem through the psyllids’ saliva. If infected, plants cannot offer an effective immune response, resulting in *C*Lso reproduction in the phloem. This blocks the nutrition transmission in the phloem, inducing erectness and stunting of new foliage, basal cupping of leaves with chlorosis and purpling, upward curving or scorching of all leaves, compressed and enlarged terminal internodes leading to resetting, hypertrophic nodes, axillary branches or tubers on the ground, breach of fruit set, and production of plenty of tiny, misshapen, and bastard fruits (Munyaneza et al., 2007a; Munyaneza et al., 2007b; Liefting et al., 2009; Secor et al., 2009; Crosslin et al., 2010; Munyaneza et al., 2010a; Munyaneza, 2012).

The underground symptoms in infected potatoes include folded stolons, browning of vascular tissue accompanying necrotic flecking of internal tissues, and dark medullary rays, all of which deteriorate throughout the tuber. Upon frying, the tuber-related symptoms are more evident, and chips and crisps present grey blotches and stripes which lead to loss of business value and privation of farmers (Munyaneza et al., 2007a; Munyaneza et al., 2007b; Secor et al., 2009; Crosslin et al., 2010; Miles et al., 2010; Goolsby et al., 2012; Munyaneza, 2012). The term “Zebra Chip disease” generally refers to those symptoms of potato tuber (Munyaneza et al., 2007a; Munyaneza et al., 2007b; Munyaneza, 2012). In carrots with infection of *C*Lso, symptoms include leaf twist and discoloration, stunting of shoots and roots, and proliferation of secondary roots (Munyaneza et al., 2010a; Munyaneza et al., 2010b; Alfaro-Fernandez et al., 2012a; Alfaro-Fernandez et al., 2012b).

Growth experiments show *C*Lso-A-infected tomato plants were stunted after three weeks of infection, but the heights of plants were not significantly less than the negative control group (Harrison et al., 2022). The *C*Lso-B-infected tomato plants’ growth was considerably impaired (Harrison et al., 2022).

Analysis shows that most of the differentially expressed genes were down-regulated in the *C*Lso samples; those genes were generally involved in plant defense against stressors, growth, plant metabolism, transport and signaling and transcription/translation (Levy et al., 2017; Harrison et al., 2022).

Among the genes for enzymes involved in cellulose synthesis in the cell wall, cellulose synthase-like A1, transferase (transferring glycosyl groups), UPA15, glycosyltransferase (CAZy family GT2), and cellulose synthase decreased in transcript abundance (FPKM) in *C*Lso-infected samples comparing to *C*Lso-free samples (Levy et al., 2017). Cell wall modification genes, expansin, XTH3, xyloglucan endotransglycosylase, and xyloglucan endotransglucosylase, were also down-regulated with *C*Lso-infection (Levy et al., 2017).

Although pathogenicity variation is observed, the host response mechanism to the pathogen has not been well studied. This study aims to describe the underlying mechanism of the functional decline of plant hosts infected by *C*Lso by analyzing the transcriptomic response of tomato plants to *C*Lso haplotypes A and B.

## 2 Materials and methods

A total of 30 ‘Moneymaker’ tomato plants were planted for four weeks, with each plant treated with five male psyllids carrying the same *C*Lso haplotype for 5-7 days in a whole plant cage. The psyllids were vectors of *C*Lso haplotype A or B. Four weeks after inoculation, the DNA of stems and leaves was extracted and purified using the MagneSil KF, Genomic kit according to the manufacturer’s protocol. All tomato plants were tested with *C*Lso PCR primers CLipoF/OI2c (Liefting et al., 2009; Secor et al., 2009) (16S rRNA), and the positive plants were also tested with the 50s rplJ/rplL primers [CL514F/CL514R (Munyaneza et al., 2009)]. PCR gel products of positive samples were extracted using QIAquick Gel Extraction Kit according to the manufacturer’s protocol and sent out for Sanger sequencing at the Ottawa Hospital Research Institute. The raw sequences were trimmed using a 0.01 quality score in CLC Genomics Workbench 20.0.2. Clasnip (the alpha version) was used to confirm *C*Lso haplotypes using the trimmed sequences (Chuan et al., 2023). Grafting was also performed using the branches of tomato plants infected by *C*Lso, which had a probability of producing newly infected plants. The new plants were tested *C*Lso with the same primers as well. Grafted plants were used to maintain the unculturable pathogen, and they were not included in the RNA-Seq samples.

Four weeks after inoculation, total RNA of stem and leaves was extracted from tomato plants infected by *C*Lso haplotype A, tomato plants infected by haplotype B, and healthy tomato plants. NGS sequencing libraries were generated from total RNA using the Illumina TruSeq Library Preparation Kit (300 bp×2) and then sequenced using the Illumina MiSeq Platform. The tomato SL4.0 genome and ITAG4.1 annotation reference were downloaded from the Sol Genomics Network (https://solgenomics.net/). Atria v2.1.0 was used to trim adapter sequences and low-quality reads (Chuan et al., 2021). Salmon v0.12.0 was used for transcript quantification (Patro et al., 2017). The ITAG4.1 coding sequence reference contained hypothetical proteins. However, these proteins lacked experimental evidence to confirm that they could be expressed *in vivo*. Therefore, coding sequences were removed if they had less than 10x coverage in half of the samples. DESeq2 was used to find differentially expressed genes (DEGs) (Love et al., 2014). DEGs were calculated while controlling for plant and batch variables (**Table 1**). The ITAG4.1 coding sequences were annotated with Gene Ontology (GO) and Kyoto Encyclopedia of Genes and Genomes (KEGG) databases using Blast2GO v1.1.0 (Gotz et al., 2008) and InterPro v70.0 (Paysan-Lafosse et al., 2023). After that, GO and KEGG pathways were enriched using DEGs and visualized with ClusterProfiler v4.2.1 (Wu et al., 2021).

**Table 1:** RNA-seq sample statistics.

Sample	Plant	Batch	Pathogen	Total Clean Read Pairs	Average Read Length	Mapping Rate (%)	Transcriptome Coverage
A10-EricLib	A10	EricLib	HA	6,895,357	130	62.3	395.2
A3-1807	A3	1807	HA	7,062,307	144	21.4	139.9
A3-1809	A3	1809	HA	6,482,136	125	21.4	128.4
A3-EricLib	A3	EricLib	HA	2,133,652	139	45.1	87.4
A5-1807	A5	1807	HA	3,493,851	146	14.5	46.8
A5-1809	A5	1809	HA	3,204,197	126	15.6	42.9
A5-EricLib	A5	EricLib	HA	2,823,550	131	64.8	168.4
A6-1807	A6	1807	HA	3,500,041	143	41.7	134.8
A6-1809	A6	1809	HA	3,228,682	124	41.5	123.7
A6-EricLib	A6	EricLib	HA	2,364,243	129	58.6	129.0
A7-1807	A7	1807	HB	4,058,312	130	5.4	18.9
A7-1809	A7	1809	HB	2,263,510	120	5.3	10.6
B4-1807	B4	1807	Negative	4,894,465	154	42.5	192.2
B4-1809	B4	1809	Negative	4,516,132	130	42.2	176.2
B5-1807	B5	1807	HB	5,381,256	120	13.7	64.0
B5-1809	B5	1809	HB	4,980,824	112	13.7	59.2
B6-1807	B6	1807	Negative	2,772,633	145	21.5	55.5
B6-1809	B6	1809	Negative	2,552,139	125	21.7	51.3
B7-1807	B7	1807	Negative	4,614,647	142	10.1	43.1
B7-1809	B7	1809	Negative	2,546,351	126	10.1	23.7
B9-1807	B9	1807	Negative	7,686,752	161	57.7	413.6
B9-1809	B9	1809	Negative	4,245,862	135	56.9	225.1
Healthy-1807	Healthy1	1807	Negative	3,526,381	149	53.5	175.7
Healthy-1809	Healthy1	1809	Negative	1,956,855	130	53.0	96.5
Healthy-EricLib	Healthy2	EricLib	Negative	2,602,756	135	60.7	145.7
T14-EricLib	T14	EricLib	HB	2,135,158	132	61.4	122.4
T5-EricLib	T5	EricLib	HB	2,071,019	136	63.3	121.3

In addition, we used qPCR analysis to validate the gene expression levels estimated from NGS sequencing. We selected 54 (including two internal control) genes to design primers using CLC Genomics Workbench 20.0.2. Those genes were DEGs or enriched in GO or KEGG pathways. Up to three primer sets were designed for each gene. The primers were preliminarily tested with normal PCR using extracted total DNA of tomato samples. Suitable primers were then used to quantify the gene expression levels between three *C*Lso haplotype B infected tomato plants and three healthy tomato plants using SensiMix II Probe Mix and Eva Green dye. Each reaction had two technical replications. A reaction would be re-run if the Ct values of technical replications were not in a reasonable range, such as the Ct difference greater than two. Delta-delta Ct value was used to compute the gene’s expression levels. The expression levels using qPCR and RNA-Seq analyses were tested using Pearson correlation.

## 3 Results and discussions

A total of 27 samples were successfully sequenced (**Table 1**). Three samples failed library preparation because of low RNA concentration. The number of clean read pairs ranged from 1.9 to 7.1 million (**Table 1**). The average read length is around 135 bp (**Table 1**). The mapping rates of tomato transcriptome varied from 5.3% to 63.3% (**Table 1**), which might be a result from diverse pathogen to host ratio and rRNA to mRNA ratio. The mean coverage of transcripts ranged from 10.6 to 413.6 (**Table 1**), indicating all samples had enough sequencing depth for RNA-Seq analysis.

### 3.1 Differentially expressed gene analysis

**Figure 1:**
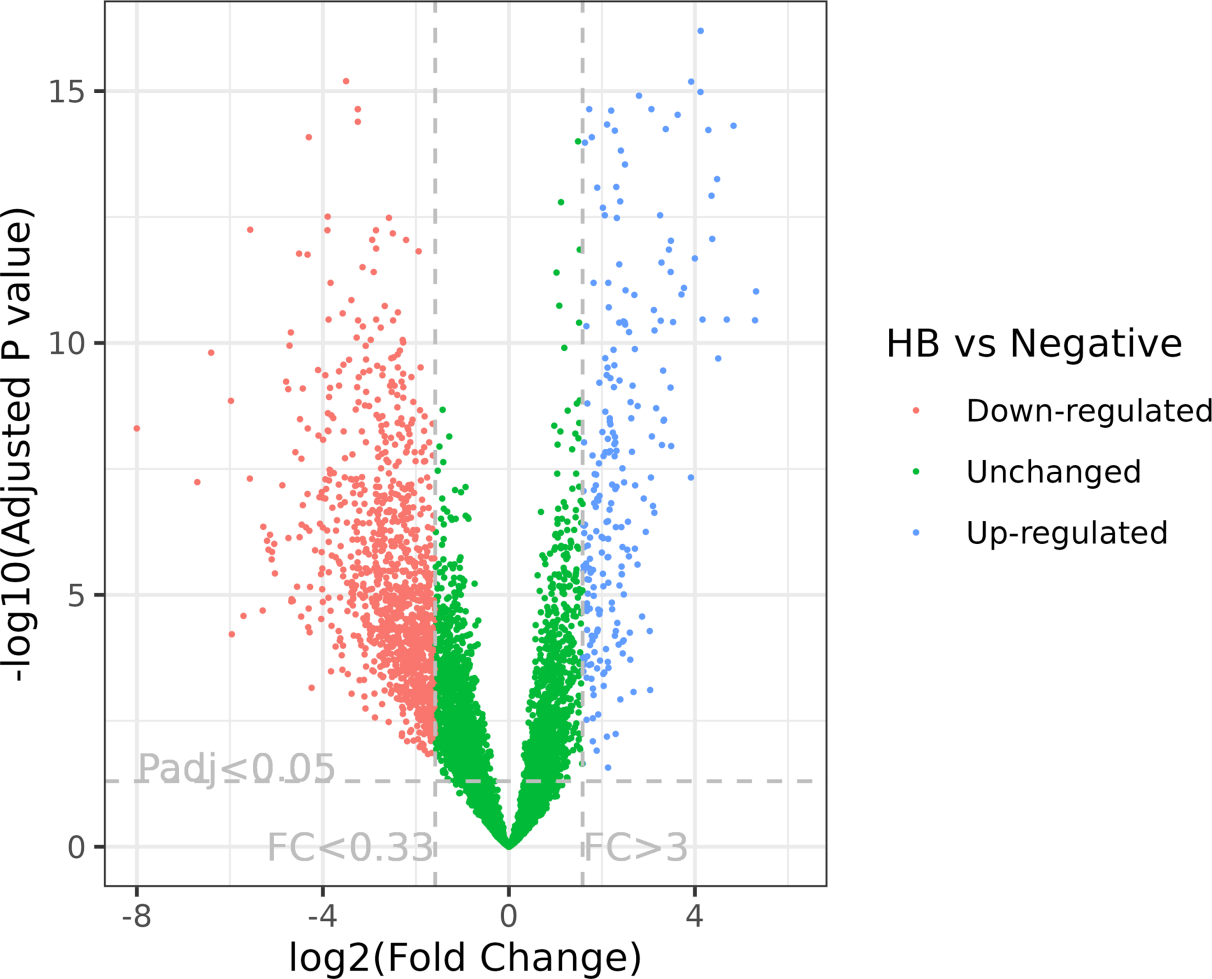
Volcano plot of RNA-Seq expression analysis of *C*Lso haplotype B versus negative plants. FC, fold change; Padj, adjusted P value. The fold change and adjusted P value cutoffs are displayed.

**Figure 2:**
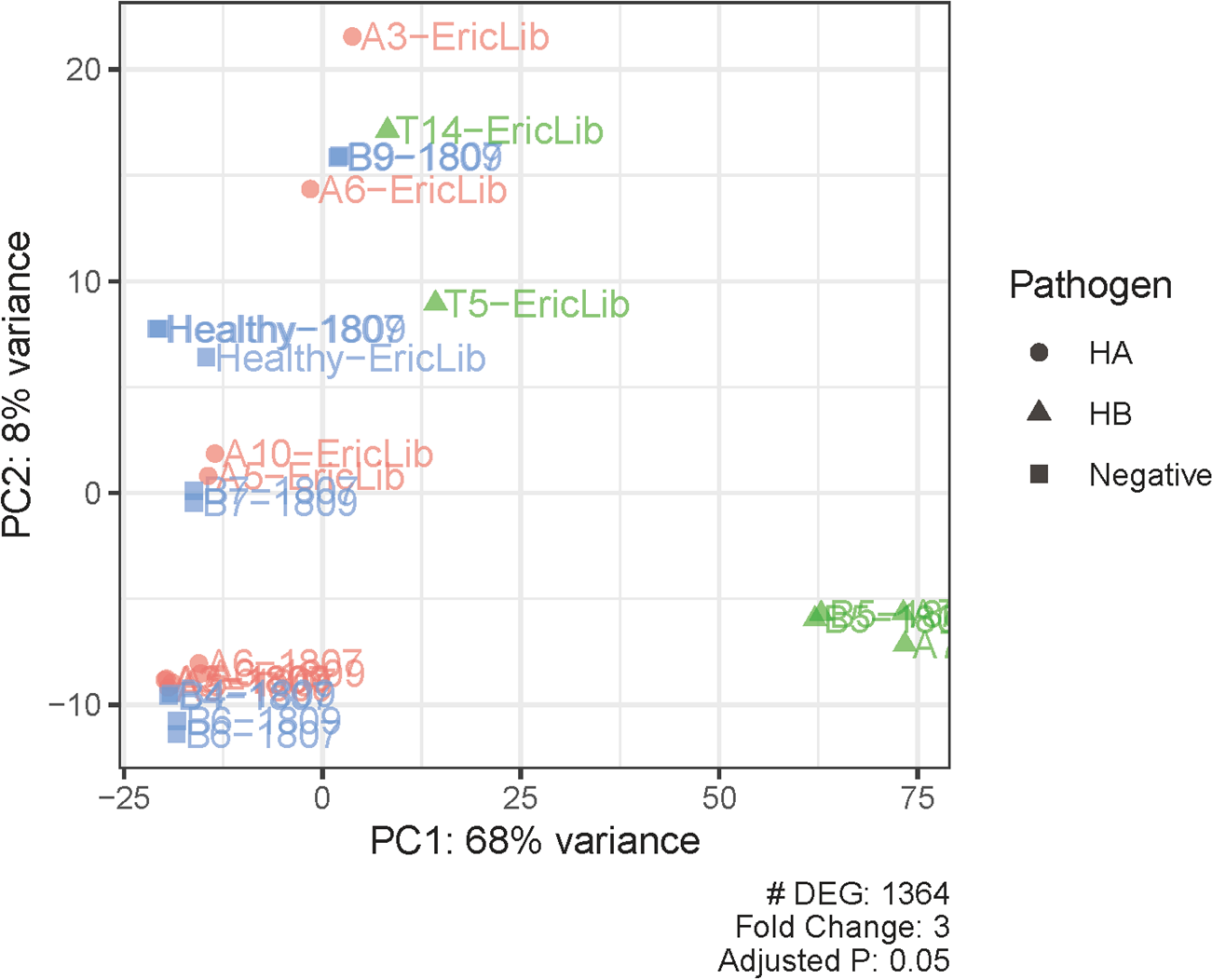
Principal component analysis of differentially expressed genes and samples. PC, principal component. Pathogen statuses are marked with colors and shapes.

**Figure 3:**
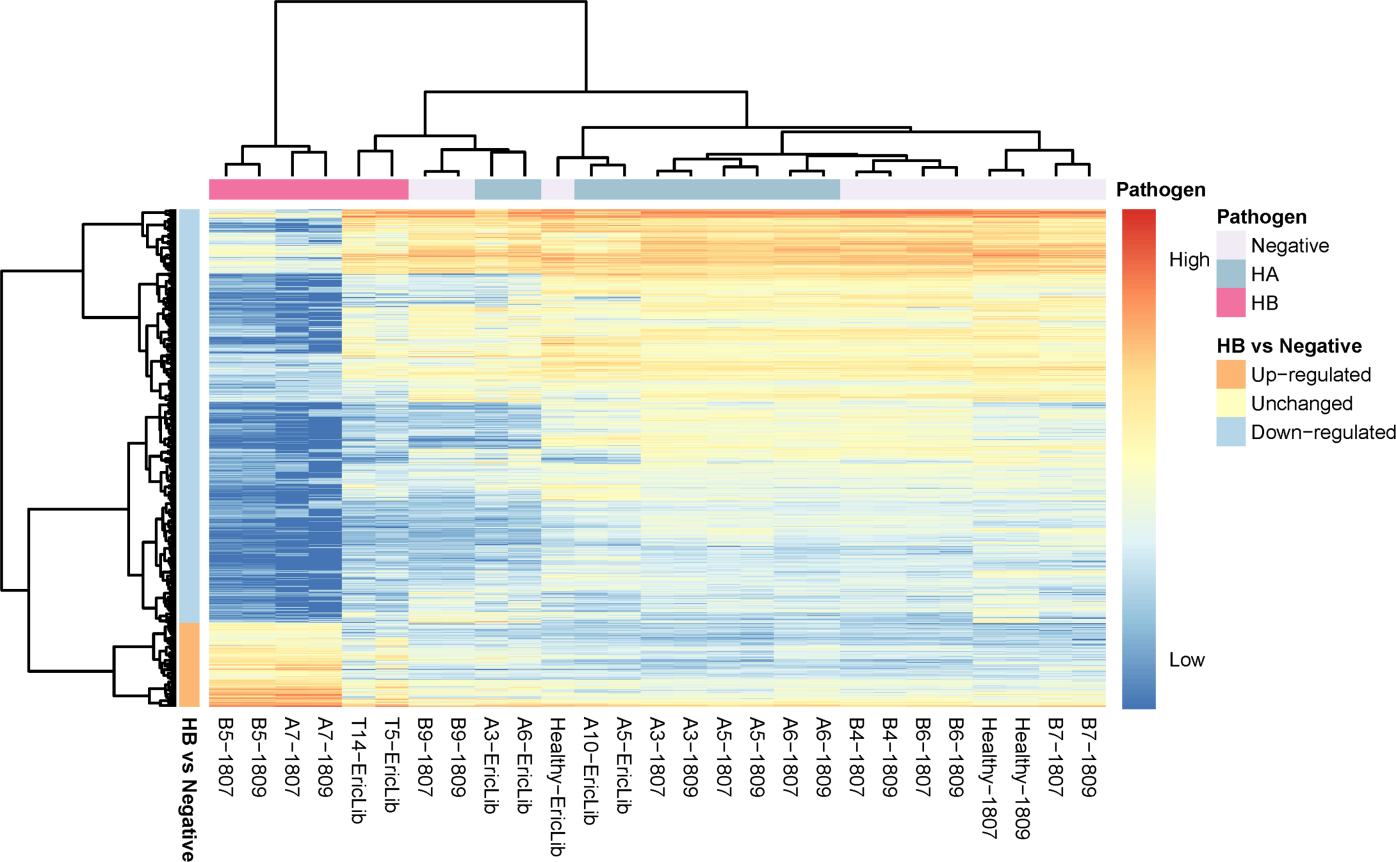
Heatmap of deferentially expressed genes of tomato hosts infected by *C*Lso haplotypes. The column labels are in the sample-batch format, and the rows are deferentially expressed genes (DEGs). The high expression level marks are red, and the low expression level is blue. The pathogen information is labeled at the top. The directions of gene expression (up-regulated, down-regulated) are labeled on the left.

In total, the expression levels of 9,888 genes in tomato plants were quantified in RNA-Seq analysis. The volcano plot illustrated the statistical significance (adjusted P value) versus the magnitude of change (fold change) of DEG results (**Figure 1**). We selected the differentially expressed genes using adjusted P value (<0.05) and fold change (≥3) (**Figures 1**–**3**; **Supplementary Table 1**). Thus, comparing haplotype B to the negative control, 1,364 DEGs were selected, among which 229 genes were upregulated and 1,135 were downregulated (**Figures 2**, **3**). The Principal Component Analysis (PCA) shows that the principal component 1 (PC1) comprised 68% variance and could be used to differentiate *C*Lso haplotype B and other groups (**Figure 2**), implying that the expression level of tomato plants infected by haplotype B varied from healthy tomato plants and those infected by *C*Lso haplotype A.

Healthy tomato plants and plants infected by haplotype A had similar expression levels, consistent with observations that *C*Lso haplotype A does not show apparent symptoms in tomato plants (**Figures 2**, **3**). In the hosts infected by *C*Lso haplotype B, the expression levels were generally in a distinct cluster compared to healthy plants and plants infected by *C*Lso haplotype A, despite the fact that T5 and T14 had relatively different patterns in gene expression compared with other haplotype B samples, which was likely the result of batch effect or latent infection (**Figures 2**, **3**).

**Figure 4:**
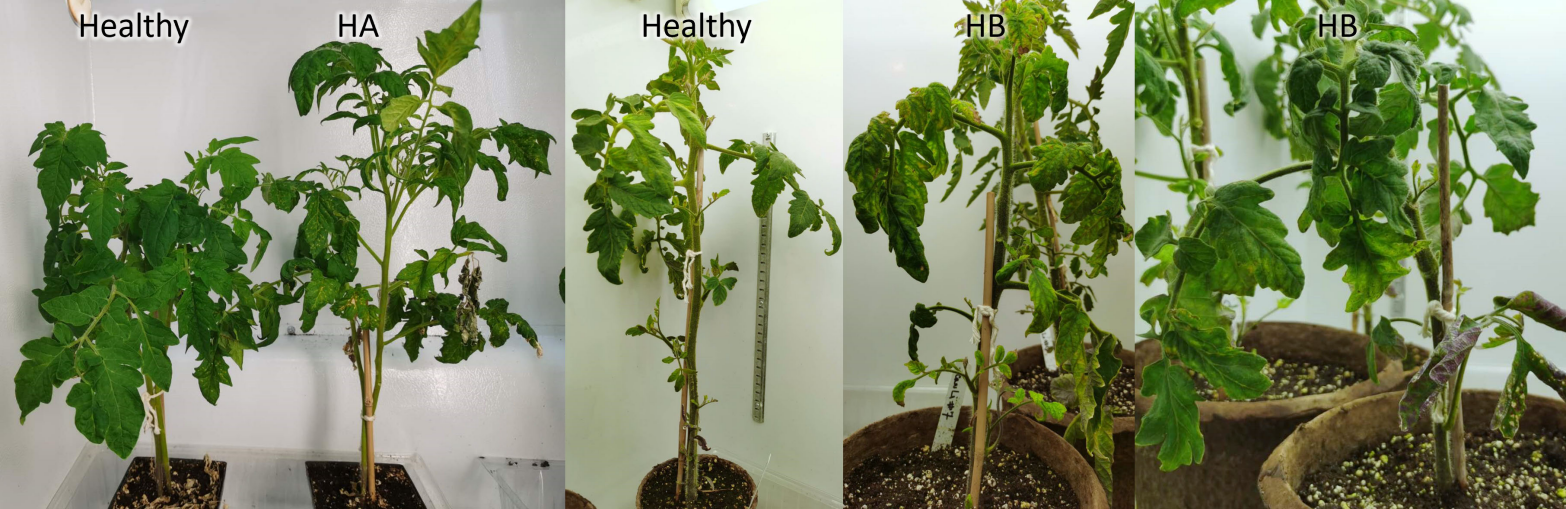
Symptoms of tomato plants infected by *C*Lso. Healthy plants were PCR negative. HA, *C*Lso haplotype A. HB, *C*Lso haplotype B.

**Figure 4** illustrates the symptoms observed in tomato plants four weeks post-inoculation. The two plants on the left belong to one batch, while the three plants on the right are from a different batch (**Figure 4**). Notably, the growth patterns of the healthy plants varied between batches (**Figure 4**). The first healthy plant, despite having larger leaves, was shorter in stature compared to the second healthy plant (**Figure 4**).

The plants with haplotype B also exhibited distinct symptoms (**Figure 4**). The entirety of the first haplotype B plant’s leaves were curled and displayed yellowing (**Figure 4**). In contrast, the second haplotype B plant had one stunted stem with scorched leaves, while its other stems appeared healthier, showing no severe curling or yellowing of the foliage (**Figure 4**).

These observations align with the latent *C*Lso infection previously reported in grafted tomato plants (Li et al., 2013). It’s worth noting that the batch of T5 and T14 differed from the other haplotype B plants, and they could be latent CLso carriers with no severe symptoms at the time of RNA extraction.

### 3.2 Enrichment analyses of GO and KEGG pathways

**Figure 5:**
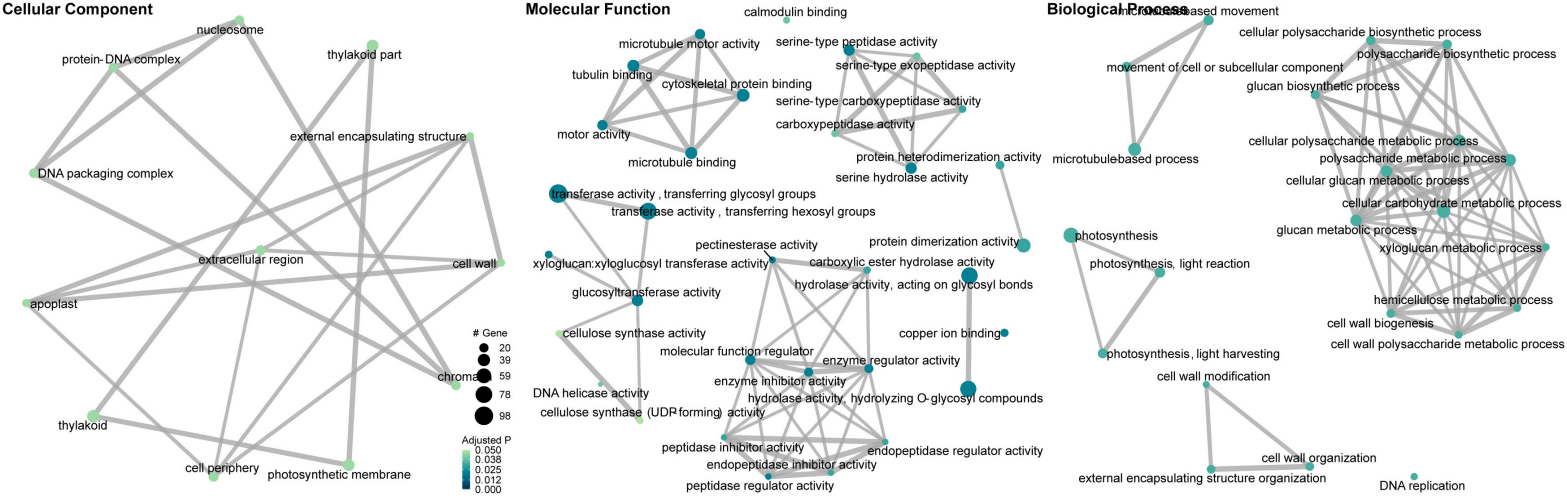
Gene ontology enrichment map and associations between gene ontology terms (*C*Lso haplotype B vs. negative). Dots are GO terms. Grey lines connect adjacent GO terms. Dot size indicates number of DEGs. Darker dot color means lower adjusted P value.

**Figure 6:**
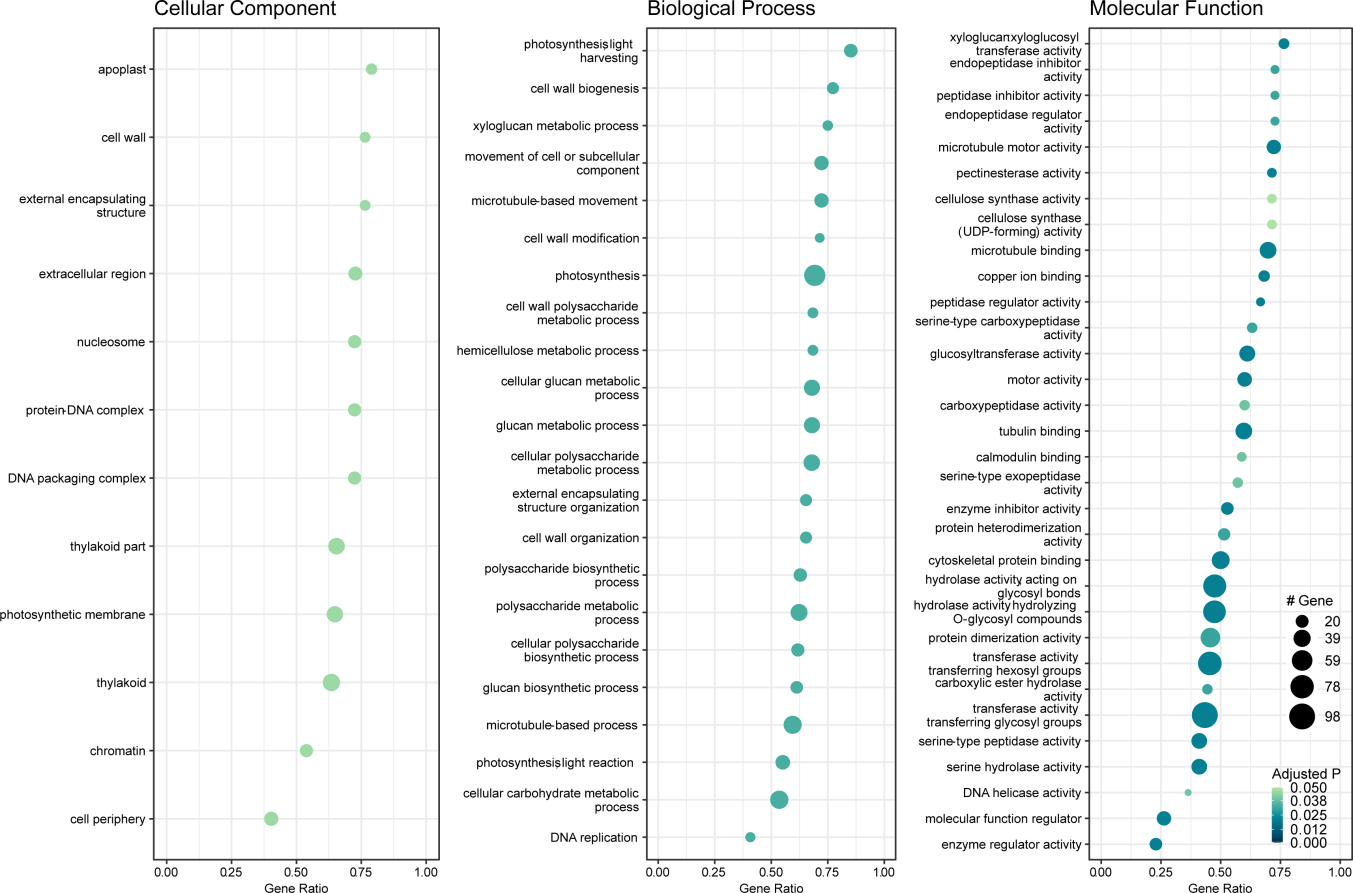
Dot plot of gene set enrichment analysis of gene ontology (GO) (*C*Lso haplotype B vs. negative). X-axis is DEG ratio. Y-axis is the GO terms. Dot size indicates the number of DEGs. Darker dot color means lower adjusted P value.

**Figure 7:**
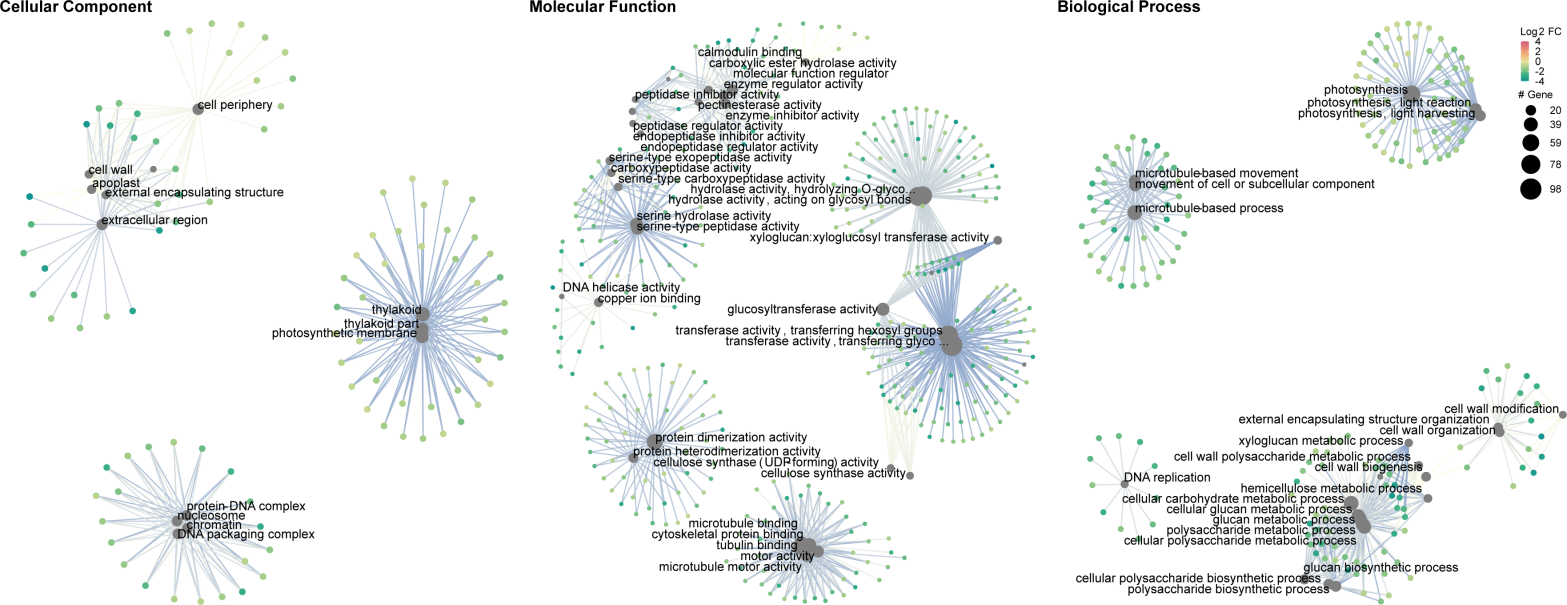
Gene concept network of gene ontology (*C*Lso haplotype B vs. negative). Big grey dots are GO terms. Sizes of grey dots indicate the number of DEGs. Small dots represent genes, colored with fold changes of gene expression levels.

Gene set enrichment analysis of gene ontology (GO) identified genes in three categories: cellular component, molecular function, and biological process (**Supplementary Table 2**). The enrichment map and associations between GO terms are illustrated in **Figure 5**. The gene ratio within each GO term is shown in **Figure 6**. The connection between GO terms and genes is depicted using a gene concept network (**Figure 7**), and the heatmaps of GO terms and detailed gene annotation are plotted (**Supplementary Figures 1**–**8**). KEGG pathways were enriched using the over-representation and gene set methods (**Supplementary Table 3**). The pathways of steroid biosynthesis, phenylpropanoid biosynthesis, flavonoid biosynthesis, DNA replication, and hormone signal transduction are shown and enriched genes are colored with expression levels (**Supplementary Figures 10**–**13**).

#### 3.2.1 Photosynthesis and carbohydrate metabolism

Plants absorb energy from sunlight to synthesize glucose in the chloroplasts, and this process is called photosynthesis. Some glucose is further processed to form starch for energy storage. The energy metabolism of plants can be altered when they are exposed to biotic stress, including bacterial pathogen invasion (Berger et al., 2004).

**Figure 8:**
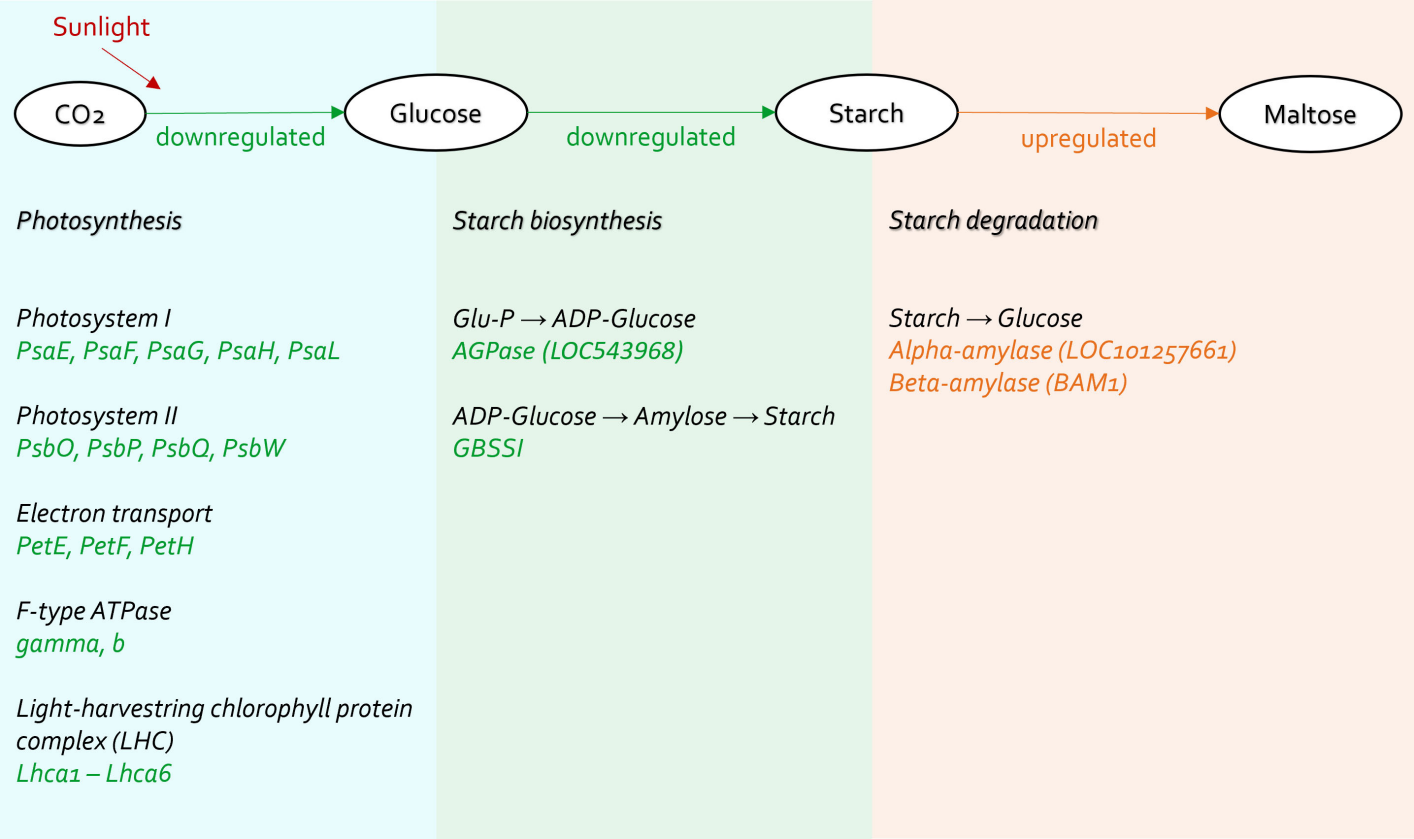
Carbohydrate metabolism affected in tomato plants infected by *C*Lso haplotype B.

Photosynthesis, especially in the light reactions, was significantly compromised in *C*Lso haplotype B infected tomato plants (**Figures 5**–**7**). The genes in the cellular components of the photosynthetic membrane and thylakoid were downregulated (**Figure 7**). Photosystems I and II are the two protein complexes with pigments to catalyze primary photosynthetic reactions. Proteins in both photosystem I (PsaE, PsaF, PsaG, PsaH, and PsaL) and photosystem II (PsbO, PsbP, PsbQ, and PsbW) were downregulated in *C*Lso haplotype B infected tomato (**Figure 8**). Electron transport proteins (PetE, PetF, and PetH) and F-type ATPases were also downregulated (**Figure 8**).

Concurrently, starch biosynthesis was significantly affected in plants infected by the pathogenic *C*Lso (**Figures 7**, **8**). Core enzymes catalyzing starch biosynthesis from glucose (AGPase [LOC543968] and GBSSI) were downregulated (**Figure 8**).

Thus, the systematic under expression of both photosynthetic and starch biosynthetic genes influenced the carbon fixation and energy harvesting of the tomato hosts, which would potentially lead to plant wilt and malnutrition.

On the contrary, starch degradation, was upregulated in tomato plants infected by *C*Lso haplotype B (**Figure 8**). Specifically, alpha-amylase (LOC101257661) and beta-amylase 1 (BAM1) were upregulated (**Figure 8**). Those alternations indicate that carbohydrate consumption in infected plants was highly active. *C*Lso encodes a glucose/galactose transporter, so it is possible to utilize glucose and galactose from host plants (Lin et al., 2011). This implies that the energy production of infected plants was insufficient for plants to grow and maintain a healthy state. The plants had to use starch to produce disaccharides and monosaccharides, which are uptaken by *C*Lso.

The impact of starch metabolism is more profound in potatoes than in tomatoes. Starch formation and accumulation affect tuber sizes, directly influencing potato yield and profit. Potato tubers infected by *C*Lso caused the conversion of potato starch to water-soluble sugars, which developed discoloration along with the vascular tissue, causing the chips to have unsightly dark blotches, stripes, or streaks after cooking (Pitman et al., 2011; Rashed et al., 2013).

#### 3.2.2 Chromatin and DNA replication

Chromatin, nucleosome, protein-DNA complexes and DNA packing complexes were enriched and clustered in cellular components (**Figure 5**). Expression variation of those components directly influences the binding factors to DNA, which are critical for gene regulation (Ramirez-Prado et al., 2018; Ahmad et al., 2022). Histones comprise protein complexes to package genomic DNA and form chromatin. Histones H1, H2A, H2B, and H3 were downregulated in *C*Lso haplotype B infected tomato plants (**Supplementary Figure 1**). The six minichromosome maintenance protein complex (MCM) proteins (MCM2 - MCM7) forming the MCM complexes were downregulated, which affected genomic DNA replication (**Supplementary Figure 12**) (Maine et al., 1984). This indicates that biotic stress suppressed the expression of histone genes and hindered gene transcription in plants (Yuan et al., 2013).

#### 3.2.3 Cell wall and plant defense

The cell wall is the outer structure that prevents bacteria from penetrating the host defense mechanisms, and is essential in growth-regulating signal transduction (Rui and Dinneny, 2020). Tomato plants infected by *C*Lso haplotype B expressed lower levels of cell wall modification and biosynthesis proteins, which might fail to prevent the bacteria from initiating direct contact with host cells. Successful adaptation to abiotic stress response in the cell wall is often related to an increased expression of xyloglucan endotransglucosylase/hydrolase (XTH) and expansin proteins (Le Gall et al., 2015). In response to pathogenic *C*Lso, the level of XTH was depressed, implying that plant defense was suppressed by the biotic stress (**Supplementary Figures 1**, **4**).

Besides xyloglucan, pectin metabolism may also play an important role in plant defense response and cell wall integrity (Wang et al., 2023a; Wang et al., 2023b). Pectin is also vital to intercellular communication and signal transduction (Shin et al., 2021). Both pectinesterase and its inhibitors are down-regulated, relating to the antagonistic action of cell-wall breakdown (**Supplementary Figure 4**).

#### 3.2.4 Steroid biosynthesis

Steroids are essential components of cell membranes, altering membrane fluidity and functioning as signaling molecules. The steroid biosynthesis pathway was significantly impaired in tomato plants infected by *C*Lso haplotype B (**Supplementary Figure 9**). Key proteins in the steroid biosynthesis pathway were all downregulated, such as sterol side chain reductase (SISSR1), sterol C14-demethylase (CPY51), and delta14-sterol reductase (FK), resulting insufficient production of cholesterol and other phytosterols (**Supplementary Figure 9**). A fungal and maize study also reports steroids are essential components of network regulation of plant immunity (Agostini et al., 2019).

#### 3.2.5 Phenylpropanoid biosynthesis

Phenylpropanoids are one of the largest classes of secondary metabolites, including flavonoids, anthocyanins, monolignols, and tannins, functioning in photosynthesis, growth regulation, nutrient process, and stress response (Pratyusha and Sarada, 2022). Phenylpropanoids are required for plant immune response to biotic and abiotic stresses (Bauters et al., 2021). In response to *C*Lso haplotype B, the gene expression levels of the tomato plants were generally suppressed, such as cinnamoyl-CoA reductase 2 (CCR2), peroxidase (CEVI-1) and hydroxycinnamoyl CoA quinate transferase (HQT) (**Supplementary Figure 10**). Carrying the pathogen, the host cannot produce sufficient secondary metabolites for plant development and defense, including scopoline, coumarin, lignin, coniferin, and syringin (**Supplementary Figure 10**).

The flavonoid biosynthesis pathway was also widely suppressed, such as chalcone synthase 1 (CHS1) and chalcone isomerase 1 (CHI1), flavanone 3-dioxygenase (F3H) and hydroxycinnamoyl CoA quinate transferase (HQT) (**Supplementary Figure 11**). The function of flavonoids spans from plant development and pigmentation to defense and signaling between plants and microorganisms (Mathesius, 2018).

#### 3.2.6 Plant hormone signal transduction

Plant hormones regulate downstream signaling components through a core pathway or independently from pathways (Bunsick et al., 2021), and are essential in plant growth, development, and stress responses (Jaillais and Chory, 2010; Ku et al., 2018).

KEGG analysis shows that disease resistance and stomatal closure pathways were promoted, and cell elongation, enlargement, and division signals were suppressed in the tomato plants infected by *C*Lso haplotype B (**Supplementary Figure 13**). Specifically, in disease resistance, NPR1 was down-regulated, while its downstream genes TGA and PR-1 were upregulated (**Supplementary Figure 13**). NPR1 is the key regulator of salicylic acid in the systemic acquired resistance pathways (Fan and Dong, 2002). In the nucleus, NPR1 interacts with TGA transcription factors to promote the expression of PR genes (Chen et al., 2019). The proteins encoded by those PR genes triggered broad-spectrum resistance to pathogens (Fan and Dong, 2002; Kesarwani et al., 2007; Ding et al., 2020). However, *C*Lso can counteract expression of NPR1 by producing salicylate hydroxylase to degrade salicylic acid (Wang et al., 2021; Levy et al., 2023). This explains why NPR1 was down-regulated in the *C*Lso-B infected tomato plants.

ABF and snRK2 were promoted for stomatal closure and seed dormancy (**Supplementary Figure 13**), which play a crucial role in plant responses to environmental stresses (Nakashima and Yamaguchi-Shinozaki, 2013; Rehman et al., 2021). The signal transduction genes of cell enlargement and plant growth were generally suppressed, such as AUX1, TIR1, IAA21, ARF, GH3, and SAUR (**Supplementary Figure 13**). Cell elongation and division genes BZR1, BZR2, TCH4 and CYCD3 were impaired (**Supplementary Figure 13**). A-AAR and B-AAR, all of which are related to cell division and shoot initiation, were suppressed (**Supplementary Figure 13**). Those suppressed genes align with the plant’s mechanism against pathogens, which can be seen as a trade-off between growth and defense, where the plant reallocates its resources from growth to defense in response to pathogen attack (Zhang et al., 2022).

### 3.3 qPCR validation

**Figure 9:**
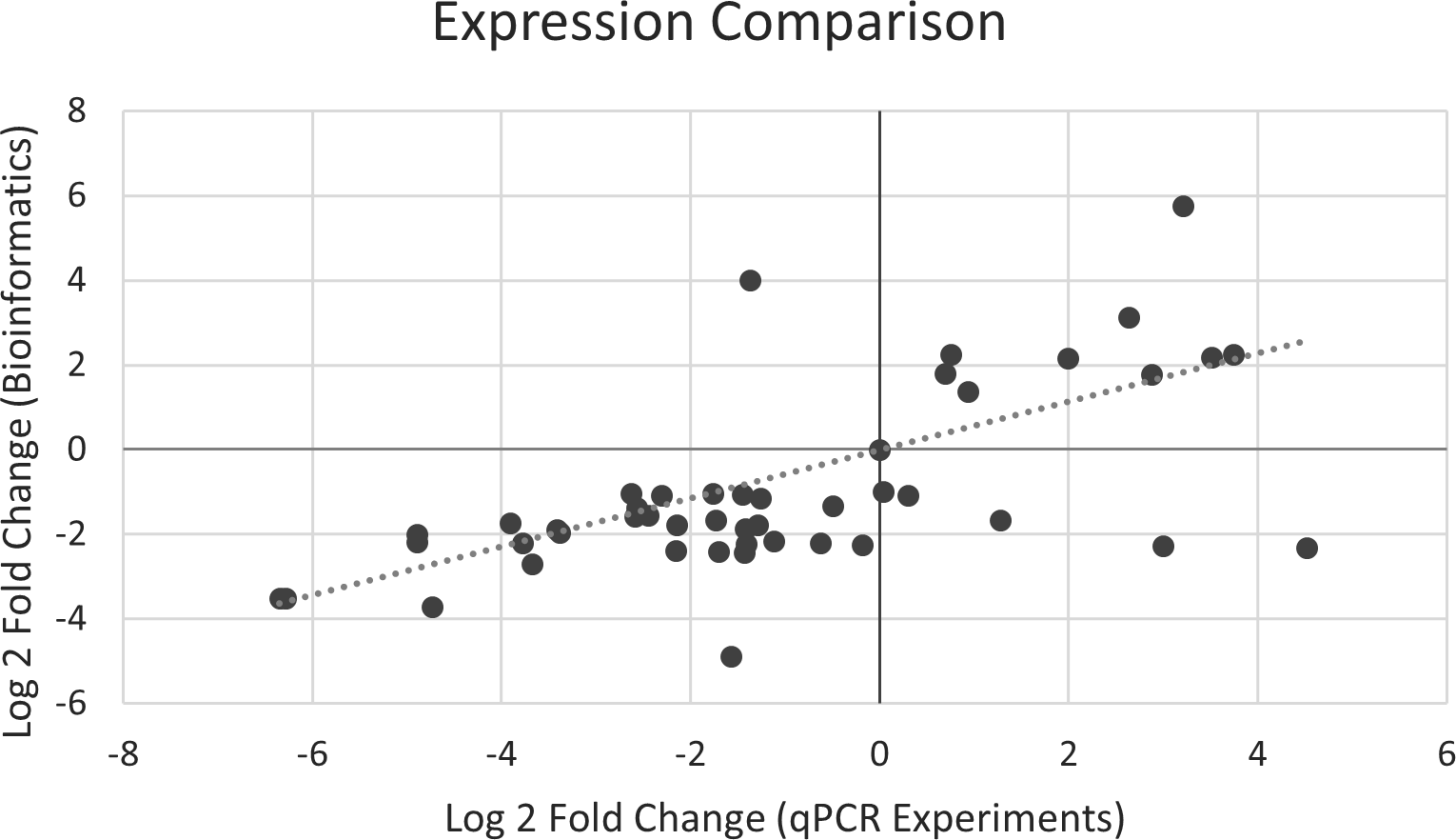
Correlation of expression magnitude between qPCR and RNA-Seq experiments.

*In silico* RNA-Seq expression was validated using qPCR analysis. We successfully designed 150 primer sets for the 54 selected genes (**Supplementary Table 4**). In the preliminary primer validation, 52 primer sets were selected for 52 genes, except that two genes, Solyc05g052280.3.1 (PSEP7) and Solyc01g107590.3.1 (CAD1), had no valid primer set (**Supplementary Table 4**). The expression levels of 47 genes were successfully generated from qPCR analysis, and five genes failed (**Supplementary Tables 4**, **5**). Among the five failed genes, two [Solyc07g052510.4.1 (PSE3) and Solyc02g083490.3.1 (PSE64)] had no ΔCt value for positive samples, Solyc04g077970.5.1 (APRT) was an unused internal control, and two [Solyc02g082930.3.1 (CHI17) and Solyc02g030170.4.1 (SISSR1)] were failed because of primer design or low concentration (**Supplementary Table 5**). The Pearson correlation coefficient between the expression levels of qPCR and Bioinformatics was 0.62 (P value = 2.75×10^-5^) (**Figure 9**), indicating the expression levels of qPCR and RNA-Seq were consistent, and the differential expression analysis of RNA-Seq was reliable.

## 4 Conclusion

This study provided insights into plants’ defense and functional decline to pathogenic *C*Lso, using whole transcriptome sequencing and qPCR validation. Our results showed how tomato plants react in metabolic pathways during the deterioration caused by pathogenic *C*Lso. Understanding the underlying mechanisms can enhance disease control and create opportunities for breeding resistant or tolerant varieties.

## Data availability statement

The original contributions presented in the study are included in the article/**Supplementary Files**, further inquiries can be directed to the corresponding author/s.

## Author contributions

JC: Data curation, Formal analysis, Methodology, Software, Validation, Visualization, Writing – original draft, Writing – review & editing. JN: Methodology, Writing – review & editing. WRC: Methodology, Resources, Writing – review & editing. WC: Data curation, Formal analysis, Resources, Software, Writing – review & editing. LH: Project administration, Resources, Supervision, Writing – review & editing. XL: Conceptualization, Methodology, Project administration, Resources, Supervision, Validation, Writing – review & editing.
