# Supplementary material for: The functional decline of tomato plants infected by Candidatus Liberbacter solanacearum: an RNA-seq transcriptomic analysis
Source: Front Plant Sci. 2024 Feb 1;15:1325254. doi: 10.3389/fpls.2024.1325254 (PMC10867784; doi:10.3389/fpls.2024.1325254)
Supplement: Supplementary file 1 [file DataSheet_1.docx]

Supplementary Material

# Supplementary Figures and Tables

## Supplementary Figures


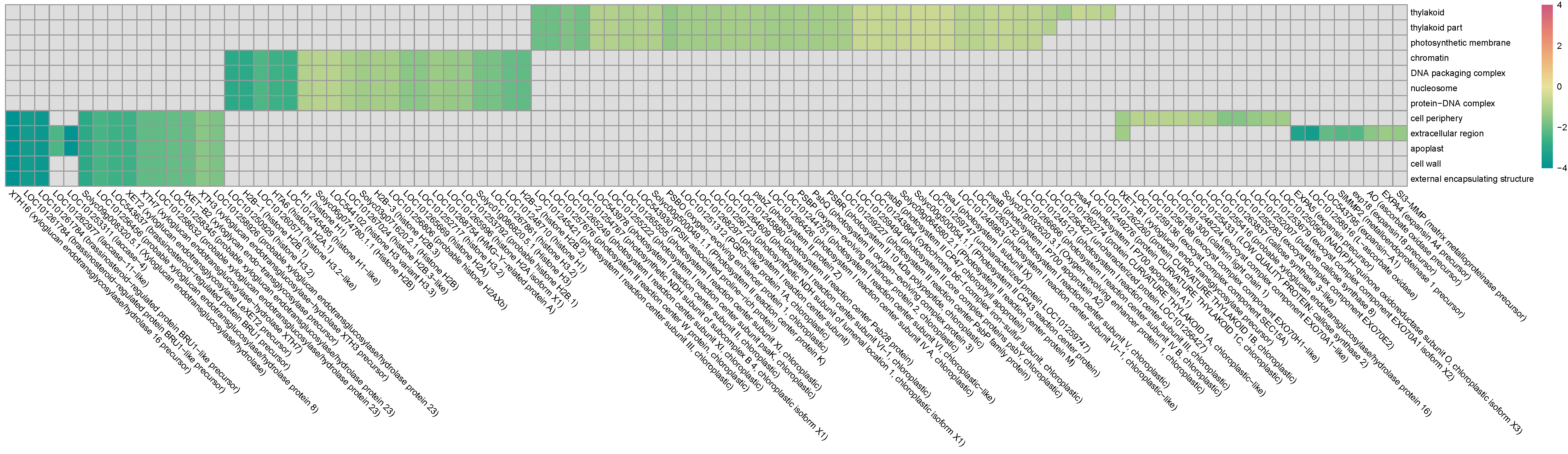


Supplementary Figure 1 Gene ontology heatmap of cellular component (*C*Lso haplotype B vs. negative).

X-axis is gene IDs and names. Y-axis is labeled with gene ontology terms. Expression levels (fold change) are colored from red (up-regulated) to green (down-regulated).


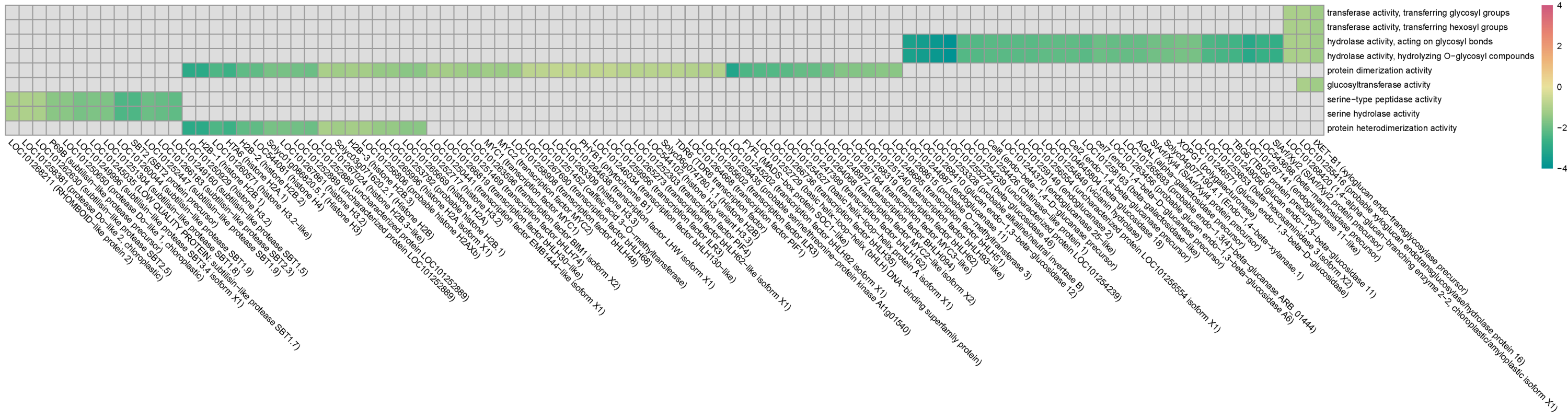


Supplementary Figure 2 Gene ontology heatmap of molecular function (*C*Lso haplotype B vs. negative) (part 1).

X-axis is gene IDs and names. Y-axis is labeled with gene ontology terms. Expression levels (fold change) are colored from red (up-regulated) to green (down-regulated).


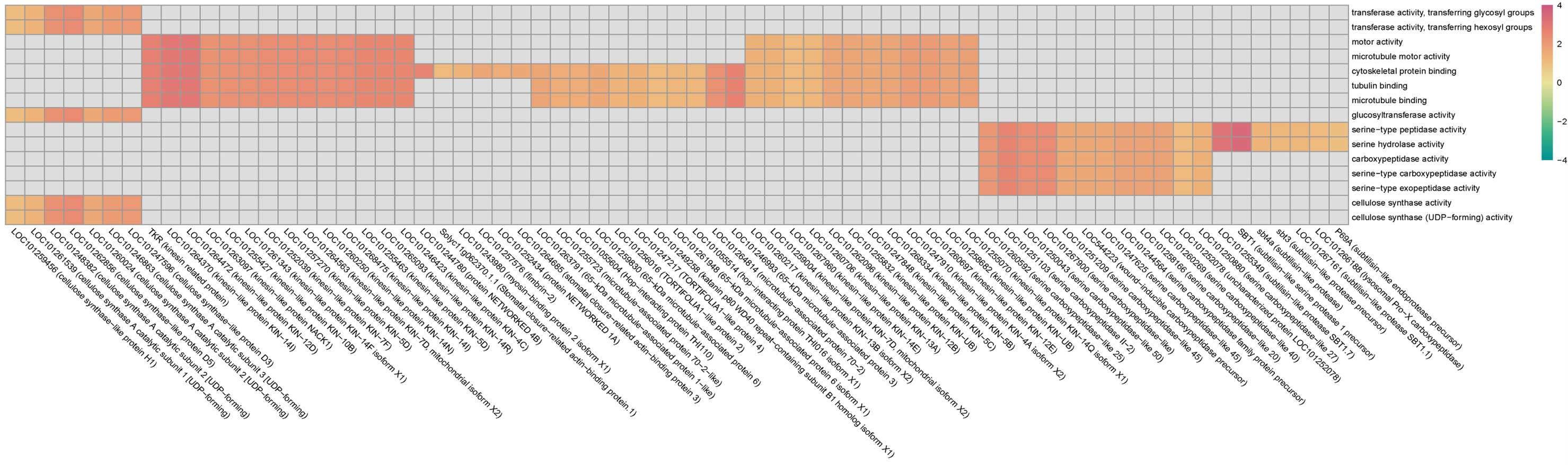


Supplementary Figure 3 Gene ontology heatmap of molecular function (*C*Lso haplotype B vs. negative) (part 2).

X-axis is gene IDs and names. Y-axis is labeled with gene ontology terms. Expression levels (fold change) are colored from red (up-regulated) to green (down-regulated).


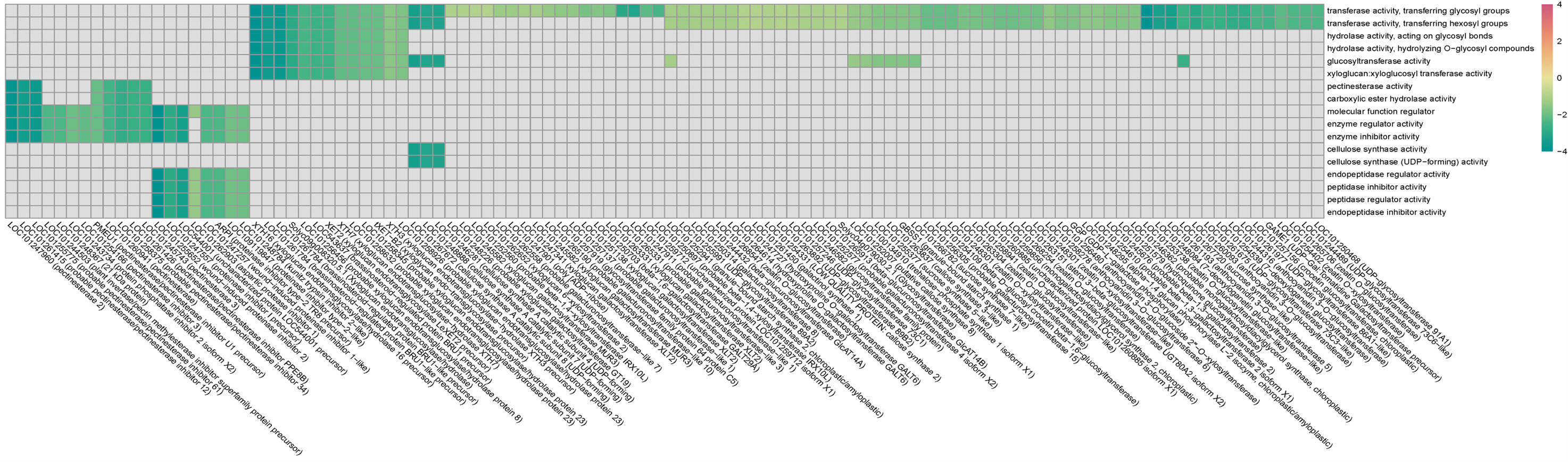


Supplementary Figure 4 Gene ontology heatmap of molecular function (*C*Lso haplotype B vs. negative) (part 3).

X-axis is gene IDs and names. Y-axis is labeled with gene ontology terms. Expression levels (fold change) are colored from red (up-regulated) to green (down-regulated).


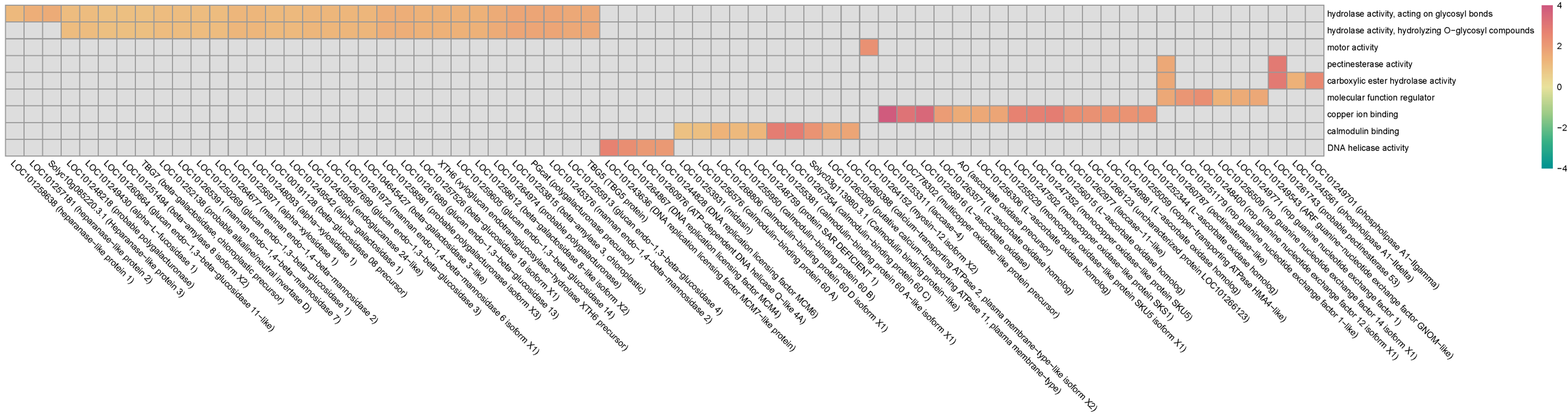


Supplementary Figure 5 Gene ontology heatmap of molecular function (*C*Lso haplotype B vs. negative) (part 4).

X-axis is gene IDs and names. Y-axis is labeled with gene ontology terms. Expression levels (fold change) are colored from red (up-regulated) to green (down-regulated).


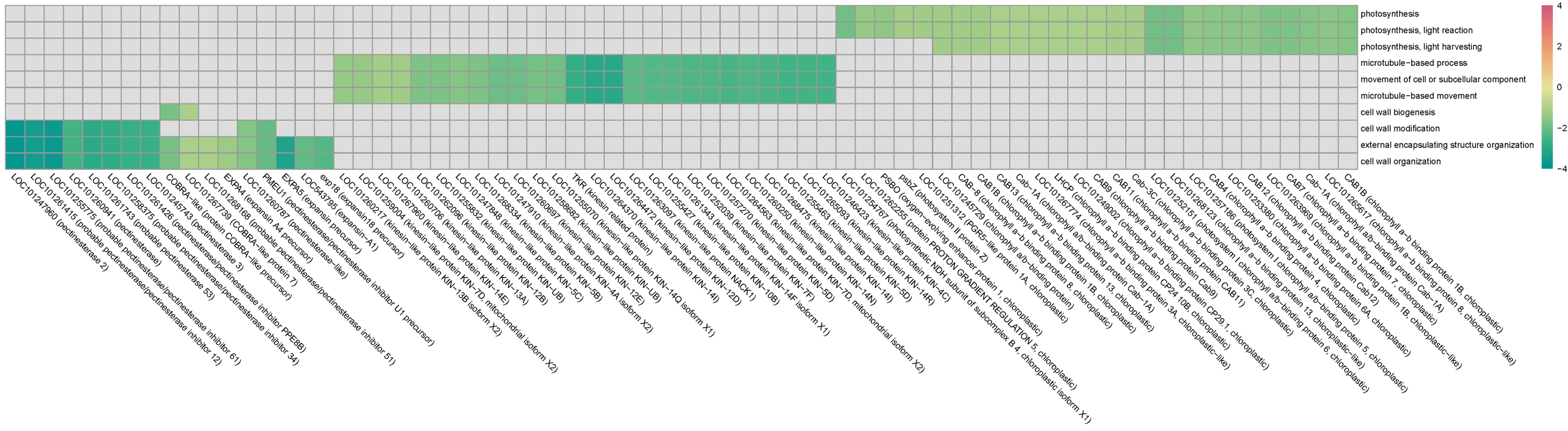


Supplementary Figure 6 Gene ontology heatmap of biological process (*C*Lso haplotype B vs. negative) (part 1).

X-axis is gene IDs and names. Y-axis is labeled with gene ontology terms. Expression levels (fold change) are colored from red (up-regulated) to green (down-regulated).


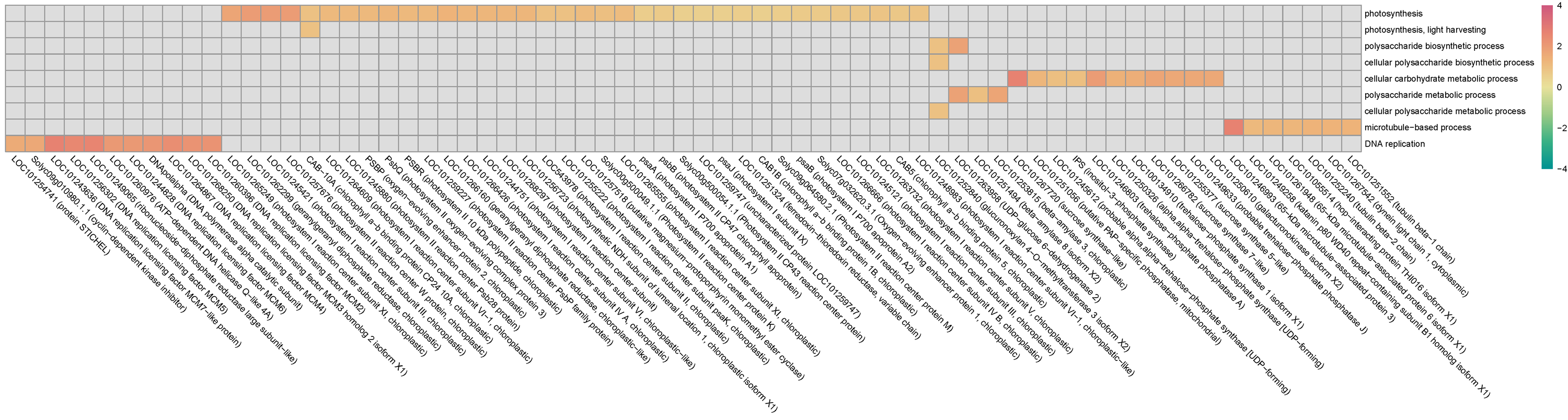


Supplementary Figure 7 Gene ontology heatmap of biological process (*C*Lso haplotype B vs. negative) (part 2).

X-axis is gene IDs and names. Y-axis is labeled with gene ontology terms. Expression levels (fold change) are colored from red (up-regulated) to green (down-regulated).


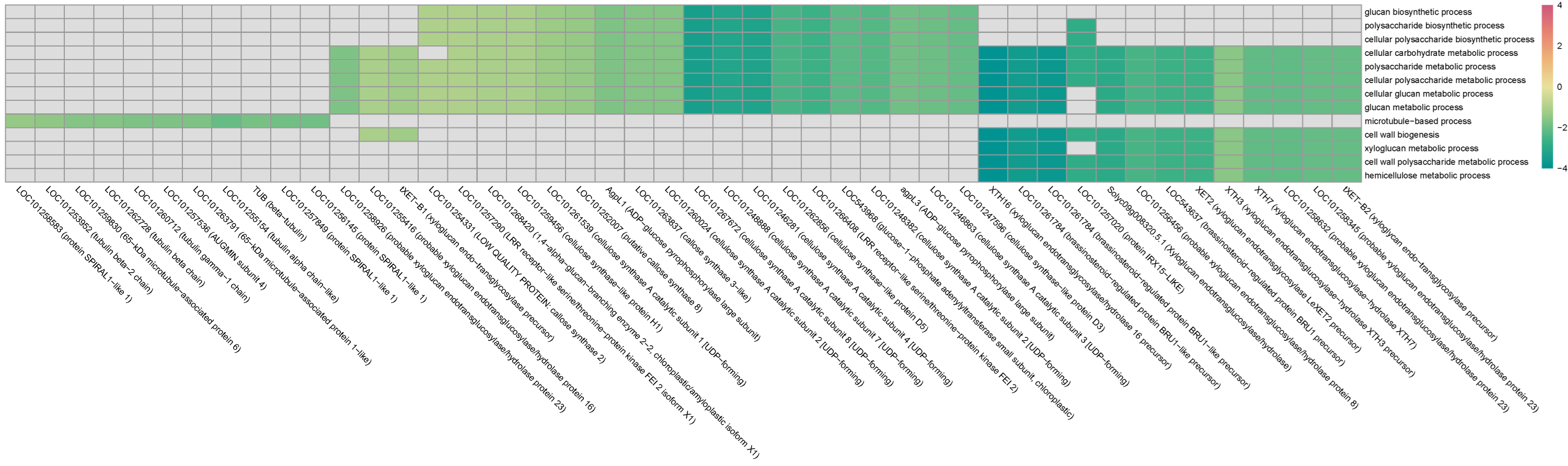


Supplementary Figure 8 Gene ontology heatmap of biological process (*C*Lso haplotype B vs. negative) (part 3).

X-axis is gene IDs and names. Y-axis is labeled with gene ontology terms. Expression levels (fold change) are colored from red (up-regulated) to green (down-regulated).


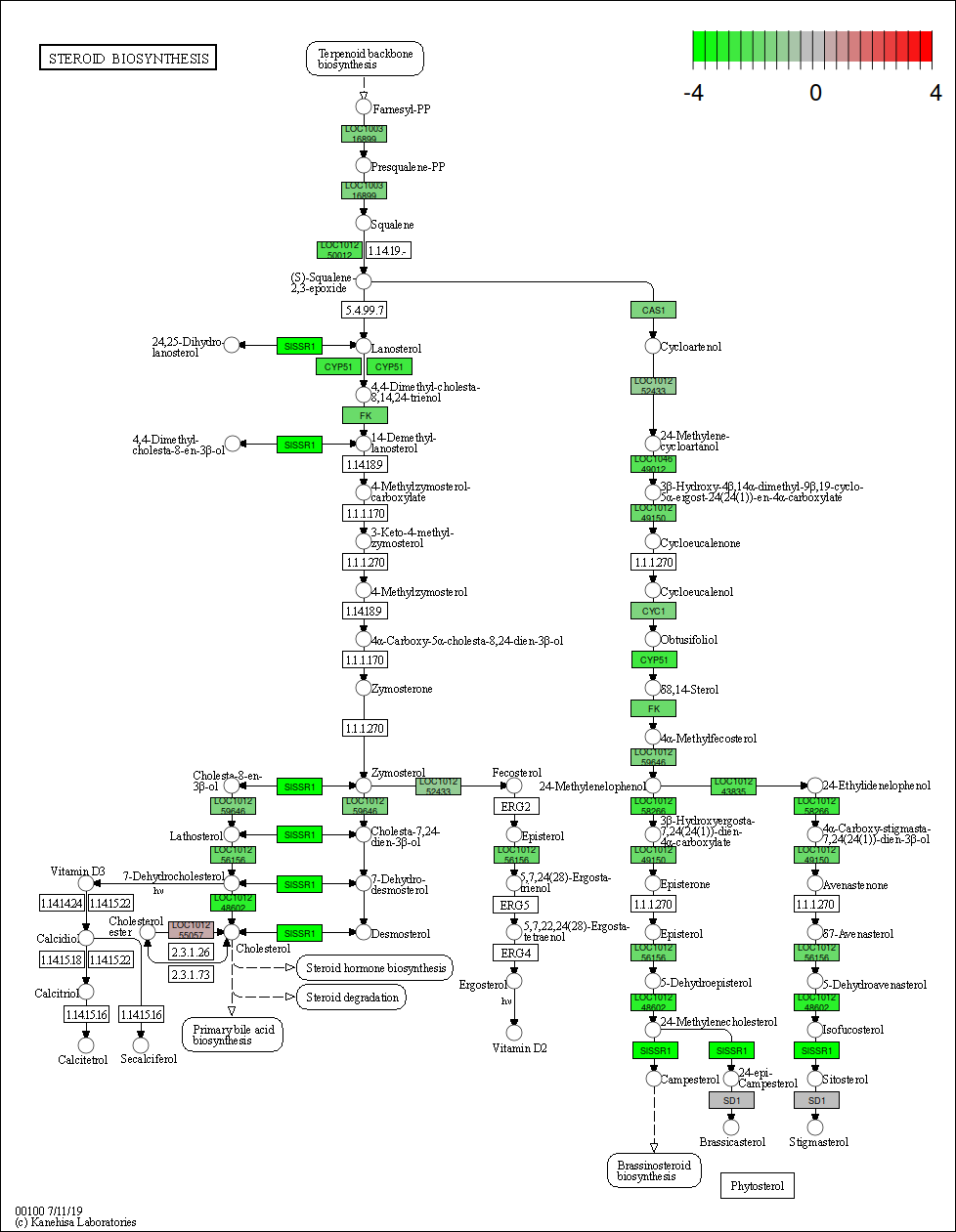


Supplementary Figure 9 Enriched KEGG pathway: steroid biosynthesis (*C*Lso haplotype B vs. negative).


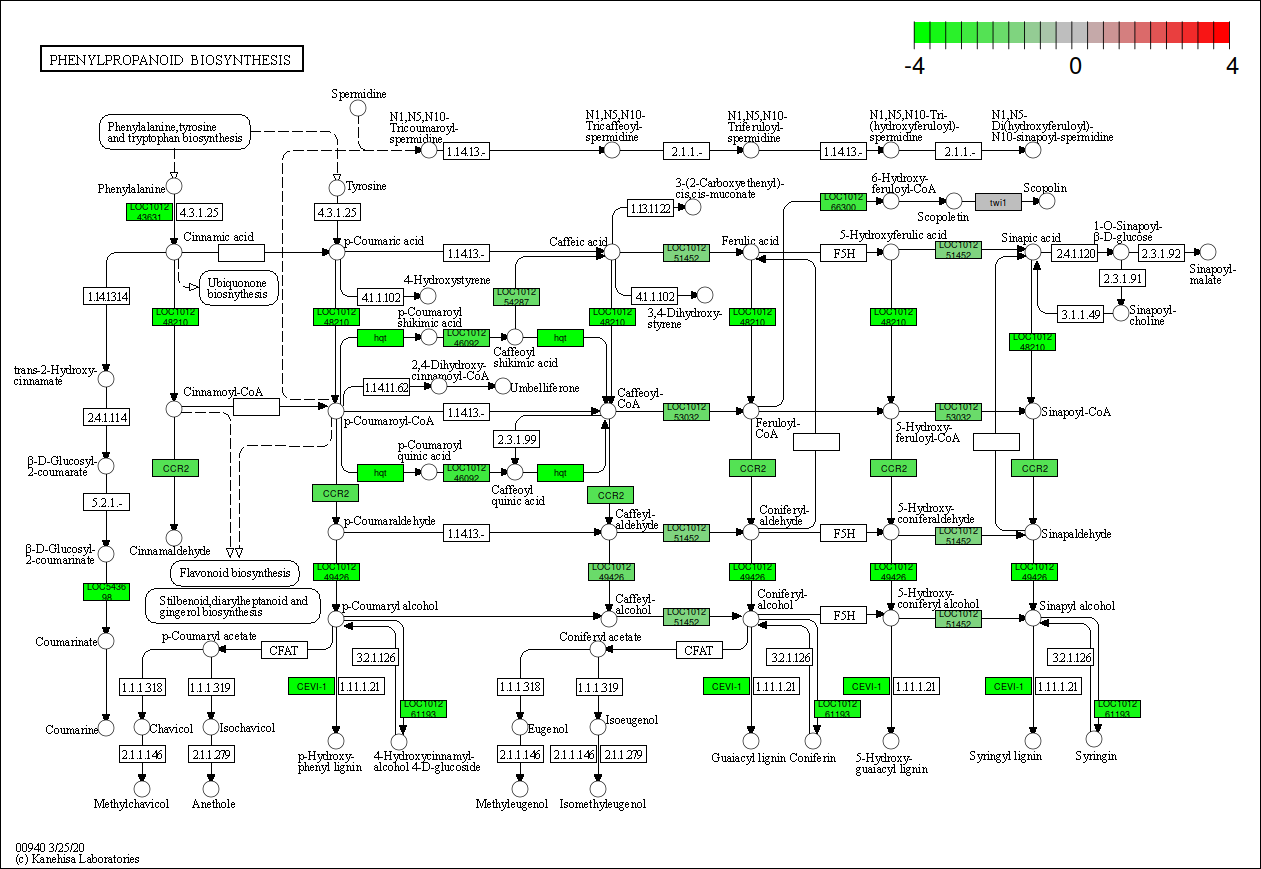


Supplementary Figure 10 Enriched KEGG pathway: phenylpropanoid biosynthesis (*C*Lso haplotype B vs. negative).


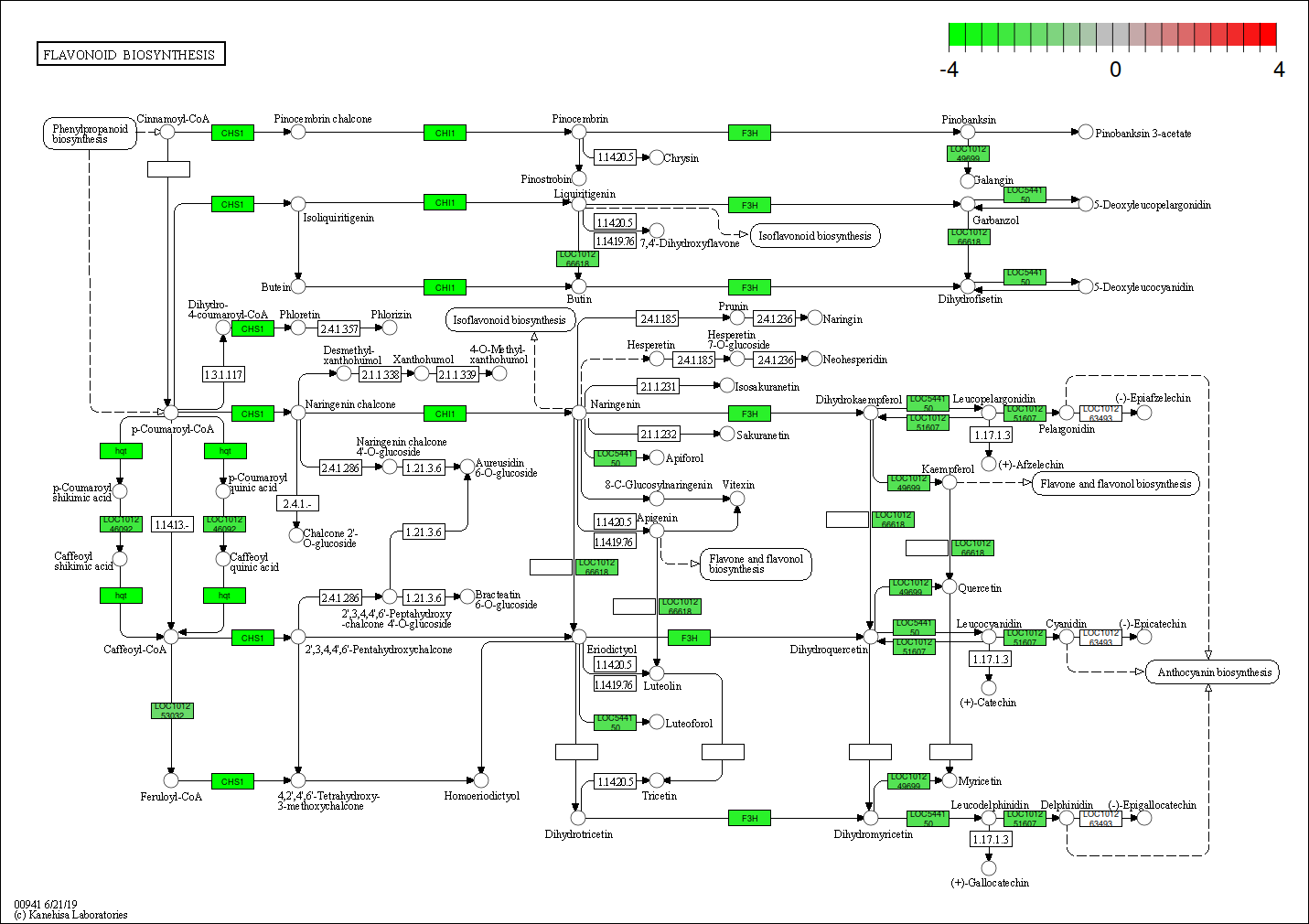


Supplementary Figure 11 Enriched KEGG pathway: flavonoid biosynthesis (*C*Lso haplotype B vs. negative).


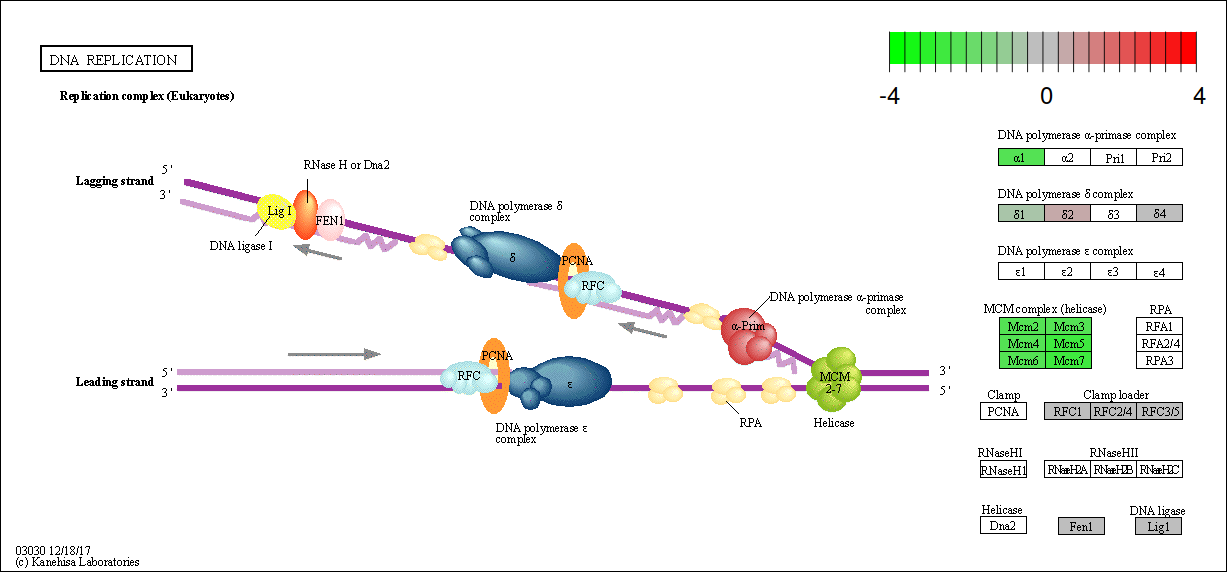


Supplementary Figure 12 Enriched KEGG pathway: DNA replication (*C*Lso haplotype B vs. negative).


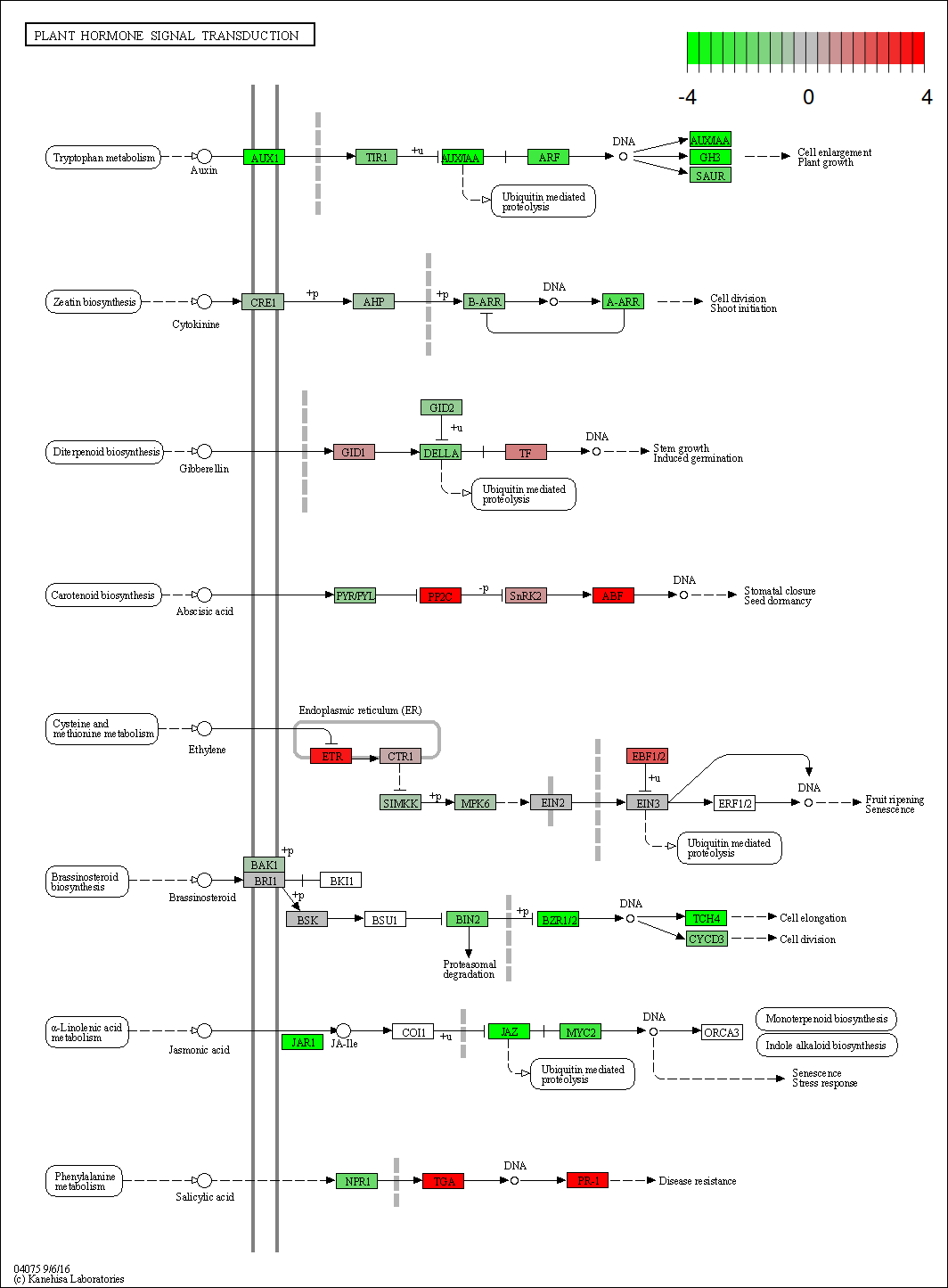


**Supplementary Figure 13 Enriched KEGG pathway: plant hormone signal transduction (*C*Lso haplotype B vs. negative).**

## Supplementary Tables

Supplementary Table 1 Gene expression analysis.

UID = unique identifier of the tomato ITAG4.1 reference transcriptome. FC = fold change. P Adj = adjusted P value.

| UID | Gene | B vs Negative | | B vs A | | A vs Negative | | Description |
| --- | --- | --- | --- | --- | --- | --- | --- | --- |
|  |  | **log2FC** | **P Adj** | **log2FC** | **P Adj** | **log2FC** | **P Adj** |  |
| Solyc10g055800.2.1 | LOC101267358 | 5.75 | 1.1E-27 | 4.35 | 2.5E-16 | 1.40 | 3.2E-02 | Chitinase |
| Solyc03g096670.3.1 | PP2C-2 | 4.34 | 4.4E-18 | 4.45 | 9.7E-20 | -0.12 | 9.1E-01 | Protein phosphatase 2C |
| Solyc02g082930.3.1 | CHI17 | 4.26 | 3.4E-11 | 4.99 | 7.6E-15 | -0.73 | 5.0E-01 | acidic extracellular 27 kD chitinase |
| Solyc04g071900.5.1 | LOC101253684 | 4.01 | 5.9E-15 | 2.34 | 2.4E-06 | 1.67 | 1.0E-02 | Peroxidase |
| Solyc07g006500.3.1 | TPS1 | 3.55 | 4.7E-08 | 4.53 | 5.1E-13 | -0.98 | 2.3E-01 | trehalose-6-phosphate synthase 1 |
| Solyc07g042550.3.1 | sus3 | 3.50 | 3.0E-15 | 2.76 | 1.8E-10 | 0.75 | 2.6E-01 | sucrose synthase |
| Solyc12g056860.2.1 | LOC101253982 | 3.16 | 3.5E-09 | 3.01 | 3.8E-09 | 0.14 | 9.7E-01 | Transcription factor TGA7 |
| Solyc03g083440.4.1 | LOC101254281 | 3.14 | 3.6E-11 | 2.91 | 1.8E-10 | 0.22 | 8.9E-01 | Glutamate synthase |
| Solyc07g043310.3.1 | GABA-TP1 | 3.12 | 1.1E-08 | 3.43 | 6.7E-11 | -0.31 | 6.7E-01 | Gamma aminobutyrate transaminase isoform 1 |
| Solyc10g055810.2.1 | CHI9 | 2.81 | 1.2E-07 | 2.32 | 1.9E-05 | 0.49 | 5.1E-01 | chitinase Z15140 |
| Solyc06g063090.4.1 | LOC101260239 | 2.65 | 6.7E-08 | 1.86 | 2.2E-04 | 0.79 | 2.0E-01 | Alanine aminotransferase 2 |
| Solyc06g071000.3.1 | SlADH12A1 | 2.53 | 6.1E-11 | 1.87 | 1.9E-06 | 0.66 | 1.8E-01 | Aldehyde dehydrogenase |
| Solyc06g059740.4.1 | ADH2 | 2.41 | 8.5E-05 | 1.68 | 9.7E-03 | 0.73 | 3.6E-01 | Alcohol dehydrogenase |
| Solyc07g040990.4.1 | LOC101261835 | 2.38 | 1.5E-13 | 2.57 | 3.3E-16 | -0.19 | 7.4E-01 | Protein phosphatase 2C 77 |
| Solyc10g076250.2.1 | LOC101268729 | 2.35 | 2.7E-06 | 2.04 | 4.6E-05 | 0.30 | 7.1E-01 | Alanine--glyoxylate aminotransferase 2 |
| Solyc01g108030.5.1 | LOC101262668 | 2.35 | 4.0E-11 | 1.94 | 5.1E-08 | 0.40 | 4.1E-01 | Methylcrotonoyl-CoA carboxylase beta chain, mitochondrial |
| Solyc11g069050.2.1 | LOC101249706 | 2.26 | 7.3E-09 | 2.68 | 2.1E-12 | -0.42 | 4.7E-01 | 4-coumarate--CoA ligase-like 7 |
| Solyc06g019170.3.1 | LOC101244293 | 2.26 | 4.5E-07 | 1.81 | 5.3E-05 | 0.45 | 5.2E-01 | Delta-1-pyrroline-5-carboxylate synthase |
| Solyc05g041200.5.1 | LOC101257377 | 2.25 | 6.5E-05 | 3.18 | 7.4E-09 | -0.94 | 2.1E-01 | 4-hydroxyphenylpyruvate dioxygenase |
| Solyc09g065540.3.1 | LOC543993 | 2.24 | 1.4E-10 | 2.14 | 4.0E-10 | 0.10 | 8.9E-01 | biotin-binding protein |
| Solyc06g053710.3.1 | ETR4 | 2.19 | 1.4E-05 | 2.06 | 4.4E-05 | 0.13 | 9.0E-01 | ethylene receptor homolog (ETR4) |
| Solyc05g007070.2.1 | LOC101257661 | 2.17 | 5.0E-10 | 2.36 | 6.5E-12 | -0.18 | 7.8E-01 | Alpha-amylase |
| Solyc11g069180.2.1 | LOC101252846 | 2.16 | 3.6E-09 | 2.20 | 9.7E-10 | -0.03 | 9.8E-01 | Isovaleryl-CoA dehydrogenase |
| Solyc05g054370.2.1 | LOC101267526 | 2.14 | 2.0E-11 | 2.08 | 5.2E-11 | 0.06 | 9.1E-01 | Acyl-CoA dehydrogenase family member 10 |
| Solyc12g014100.2.1 | LOC543605 | 2.13 | 8.0E-09 | 2.20 | 9.7E-10 | -0.07 | 9.3E-01 | homogentisate 1,2-dioxygenase |
| Solyc09g098590.4.1 | SUS4 | 2.13 | 7.7E-07 | 2.29 | 8.1E-08 | -0.16 | 8.7E-01 | Sucrose synthase |
| Solyc01g102300.3.1 | LOC101267355 | 2.07 | 2.0E-10 | 2.69 | 5.3E-17 | -0.62 | 1.8E-01 | bHLH transcription factor 006 |
| Solyc03g122310.4.1 | LOC101252968 | 2.01 | 2.1E-13 | 1.70 | 4.8E-10 | 0.32 | 4.3E-01 | Aldehyde dehydrogenase |
| Solyc08g082440.3.1 | LOC101254509 | 1.92 | 6.2E-10 | 2.13 | 2.1E-11 | -0.21 | 8.0E-01 | UDP-glucose 4-epimerase |
| Solyc12g099440.3.1 | LOC101263592 | 1.90 | 8.3E-14 | 1.66 | 5.7E-11 | 0.24 | 5.4E-01 | Glyoxysomal fatty acid beta-oxidation multifunctional protein MFP-a |
| Solyc07g008250.3.1 | LOC101248690 | 1.89 | 5.3E-05 | 1.90 | 8.1E-05 | -0.01 | 9.6E-01 | F-box protein |
| Solyc09g091470.3.1 | LOC101255924 | 1.88 | 4.1E-08 | 1.71 | 6.5E-07 | 0.18 | 7.6E-01 | 3-ketoacyl-CoA thiolase peroxisomal-like |
| Solyc07g006650.4.1 | LOC101266649 | 1.83 | 6.4E-12 | 1.44 | 4.4E-08 | 0.39 | 3.0E-01 | Xylose isomerase |
| Solyc02g079500.4.1 | TMP1 | 1.81 | 8.0E-05 | 1.70 | 1.2E-04 | 0.11 | 9.6E-01 | Peroxidase |
| Solyc08g068330.4.1 | LOC101260072 | 1.77 | 1.9E-05 | 1.92 | 3.5E-06 | -0.15 | 8.9E-01 | Aspartate aminotransferase |
| Solyc01g108080.4.1 | LOC101263766 | 1.67 | 4.0E-07 | 1.36 | 2.0E-05 | 0.31 | 5.8E-01 | ABSCISIC ACID-INSENSITIVE 5-like protein 7 |
| Solyc04g025940.3.1 | LOC101262611 | 1.64 | 1.1E-14 | 1.94 | 1.7E-20 | -0.30 | 3.7E-01 | Trehalose-6-phosphate synthase |
| Solyc01g102660.4.1 | LOC101248052 | 1.63 | 2.7E-06 | 1.64 | 1.9E-06 | -0.01 | 1.0E+00 | Glutathione S-transferase |
| Solyc10g076600.2.1 | LOC101248057 | 1.62 | 5.9E-07 | 0.85 | 1.1E-02 | 0.77 | 4.3E-02 | Acyl-CoA oxidase/dehydrogenase |
| Solyc08g069231.1.1 | LOC101245341 | 1.61 | 8.2E-06 | 1.87 | 1.6E-07 | -0.26 | 6.9E-01 | L-arabinokinase |
| Solyc01g090693.1.1 | LOC101265771 | 1.59 | 2.1E-05 | 1.40 | 2.5E-04 | 0.20 | 7.2E-01 | 3-hydroxyisobutyryl-CoA hydrolase-like protein 1, mitochondrial |
| Solyc00g500054.1.1 |  | -0.52 | 1.1E-01 | 0.07 | 8.9E-01 | -0.59 | 1.5E-01 | Photosystem II CP43 reaction center protein |
| Solyc05g025600.1.1 | LOC101259747 | -0.54 | 3.8E-01 | -1.03 | 4.4E-02 | 0.49 | 4.6E-01 | Photosystem II reaction center X protein |
| Solyc09g064400.3.1 | psaJ | -0.55 | 2.4E-01 | -0.55 | 2.0E-01 | 0.00 | 9.8E-01 | Photosystem I reaction center subunit IX |
| Solyc02g071030.2.1 | CAB1B | -0.56 | 1.7E-01 | -0.16 | 6.4E-01 | -0.40 | 5.5E-01 | Chlorophyll a-b binding protein, chloroplastic |
| Solyc09g064580.2.1 |  | -0.57 | 4.4E-01 | -0.60 | 3.5E-01 | 0.03 | 9.5E-01 | Photosystem II reaction center protein M |
| Solyc01g087520.3.1 | LOC101251324 | -0.59 | 8.5E-02 | -0.32 | 3.7E-01 | -0.28 | 5.4E-01 | Ferredoxin-thioredoxin reductase, variable chain |
| Solyc10g077120.3.1 | LOC101259494 | -0.61 | 1.1E-01 | -0.11 | 7.2E-01 | -0.50 | 3.6E-01 | Photosystem II core complex proteins psbY, chloroplastic |
| Solyc00g500057.1.1 | psaA | -0.63 | 1.0E-01 | 0.08 | 8.7E-01 | -0.70 | 1.3E-01 | Photosystem I P700 chlorophyll a apoprotein .1 |
| Solyc12g005630.2.1 | LOC101243864 | -0.63 | 2.0E-01 | -0.59 | 2.0E-01 | -0.04 | 9.9E-01 | Cytochrome b6-f complex iron-sulfur subunit |
| Solyc00g500024.1.1 | psbB | -0.65 | 1.7E-01 | 0.53 | 2.9E-01 | -1.17 | 2.7E-02 | Photosystem II CP47 reaction center protein |
| Solyc04g074580.1.1 | LOC101263309 | -0.69 | 5.7E-03 | -0.64 | 8.2E-03 | -0.05 | 9.4E-01 | Histone H3 |
| Solyc06g066000.3.1 | LOC109120519 | -0.69 | 8.8E-02 | -0.13 | 7.3E-01 | -0.56 | 2.9E-01 | ATP synthase subunit b', chloroplastic |
| Solyc00g500056.1.1 | psaB | -0.70 | 9.4E-02 | -0.03 | 9.4E-01 | -0.66 | 2.0E-01 | DNA-directed RNA polymerase subunit alpha |
| Solyc06g074110.3.1 | LOC101264638 | -0.71 | 7.7E-02 | -0.50 | 2.2E-01 | -0.21 | 7.1E-01 | Transcription factor |
| Solyc07g032620.3.1 |  | -0.71 | 1.2E-01 | -0.39 | 4.0E-01 | -0.32 | 6.0E-01 | Oxygen-evolving enhancer protein 1, chloroplastic |
| Solyc12g100140.3.1 | LOC101246034 | -0.72 | 6.3E-02 | -0.44 | 2.5E-01 | -0.28 | 5.9E-01 | Transcription factor bHLH80 |
| Solyc07g064040.3.1 | LOC101259956 | -0.72 | 2.2E-02 | -0.83 | 5.6E-03 | 0.11 | 8.5E-01 | bHLH transcription factor143 |
| Solyc01g059870.4.1 | PHYB1 | -0.72 | 1.1E-02 | -0.66 | 1.9E-02 | -0.06 | 8.9E-01 | phytochrome B1 |
| Solyc03g120530.3.1 | LOC101267690 | -0.74 | 8.7E-02 | -0.39 | 3.8E-01 | -0.35 | 5.0E-01 | Basic helix-loop-helix (BHLH) DNA-binding superfamily protein |
| Solyc09g063130.3.1 | LOC101266666 | -0.74 | 5.9E-02 | -0.51 | 2.0E-01 | -0.23 | 7.1E-01 | Photosystem I reaction center subunit IV |
| Solyc03g080180.5.1 | LOC101251452 | -0.75 | 4.8E-02 | -1.04 | 4.4E-03 | 0.29 | 6.2E-01 | O-methyltransferase, putative |
| Solyc06g066620.4.1 | LOC101249276 | -0.75 | 5.3E-02 | -0.51 | 2.1E-01 | -0.25 | 6.3E-01 | Protein CURVATURE THYLAKOID 1C, chloroplastic |
| Solyc02g069460.3.1 | LOC101245121 | -0.76 | 7.8E-02 | -0.91 | 2.6E-02 | 0.15 | 8.4E-01 | Photosystem I reaction center subunit III, chloroplastic |
| Solyc05g050500.1.1 | LOC101249084 | -0.77 | 1.1E-01 | 0.47 | 5.5E-01 | -1.24 | 4.4E-02 | ATP synthase delta chain chloroplastic-like |
| Solyc12g006140.2.1 | CAB5 | -0.78 | 6.4E-02 | -0.48 | 2.1E-01 | -0.30 | 7.1E-01 | Cab-5 gene encoding chlorophyll a/b-binding protein |
| Solyc09g057710.5.1 | LOC101265273 | -0.82 | 2.9E-02 | -0.73 | 4.6E-02 | -0.09 | 8.7E-01 | Basic helix-loop-helix (BHLH) DNA-binding superfamily protein |
| Solyc06g066640.3.1 | LOC101248983 | -0.82 | 9.3E-02 | -0.47 | 2.9E-01 | -0.35 | 7.0E-01 | Photosystem I reaction center subunit VI, chloroplastic |
| Solyc12g096290.3.1 | LOC101259136 | -0.83 | 4.1E-02 | -0.83 | 3.4E-02 | 0.00 | 9.9E-01 | Exocyst complex component |
| Solyc10g005050.3.1 | LOC101262260 | -0.83 | 1.4E-01 | -1.08 | 3.7E-02 | 0.25 | 7.4E-01 | Protein CURVATURE THYLAKOID 1B, chloroplastic |
| Solyc07g043580.4.1 | LOC101252303 | -0.83 | 5.3E-03 | -0.08 | 8.0E-01 | -0.75 | 2.0E-02 | bHLH transcription factor 052 |
| Solyc06g074780.1.1 |  | -0.84 | 4.1E-02 | -1.44 | 1.7E-04 | 0.60 | 1.9E-01 | Histone H2B |
| Solyc02g084630.3.1 | TDR6 | -0.84 | 3.4E-02 | -0.53 | 2.0E-01 | -0.32 | 5.6E-01 | TDR6 transcription factor |
| Solyc03g006510.5.1 | LOC101261300 | -0.85 | 2.2E-03 | -0.74 | 6.9E-03 | -0.12 | 7.7E-01 | Clathrin light chain |
| Solyc05g051500.4.1 | LOC544102 | -0.85 | 2.5E-02 | -1.01 | 4.1E-03 | 0.15 | 6.8E-01 | Histone H3 |
| Solyc07g066150.1.1 | LOC101263732 | -0.86 | 5.4E-02 | -0.87 | 5.1E-02 | 0.01 | 9.9E-01 | Photosystem I reaction center subunit V, chloroplastic |
| Solyc08g069100.1.1 | LOC101248224 | -0.86 | 3.9E-02 | -1.05 | 7.8E-03 | 0.19 | NA | Exocyst subunit Exo70 family protein |
| Solyc06g075610.1.1 | LOC101256188 | -0.87 | 5.2E-03 | -1.29 | 1.4E-05 | 0.42 | 2.9E-01 | Exocyst subunit Exo70 family protein |
| Solyc00g500322.1.1 | atpF | -0.89 | 2.1E-02 | -0.79 | 3.7E-02 | -0.10 | 9.1E-01 | ATP synthase subunit b, chloroplastic |
| Solyc00g500064.1.1 | atpB | -0.90 | 3.6E-02 | -0.33 | 4.3E-01 | -0.56 | 3.6E-01 | Ycf15 |
| Solyc06g084020.4.1 | H1 | -0.90 | 2.3E-02 | -1.13 | 3.3E-03 | 0.23 | 7.4E-01 | histone H1 |
| Solyc02g079970.4.1 | LOC101265602 | -0.92 | 6.0E-03 | -0.99 | 2.5E-03 | 0.08 | 9.3E-01 | bHLH transcription factor 014 |
| Solyc00g500333.1.1 | atpF | -0.92 | 6.2E-02 | 0.29 | 7.3E-01 | -1.21 | 5.1E-02 | ATP synthase subunit b, chloroplastic |
| Solyc01g105030.3.1 | CAB-10A | -0.92 | 3.3E-02 | -0.79 | 6.6E-02 | -0.13 | 8.7E-01 | Chlorophyll a-b binding protein, chloroplastic |
| Solyc00g500140.1.1 | atpE | -0.92 | 3.9E-02 | -0.30 | 4.7E-01 | -0.61 | 3.4E-01 | ATP synthase epsilon chain, chloroplastic |
| Solyc01g109040.4.1 | LOC101256427 | -0.93 | 1.0E-01 | -1.04 | 5.5E-02 | 0.11 | 9.1E-01 | Cytochrome b6-f complex subunit 7 |
| Solyc11g031950.1.1 | LOC101252840 | -0.93 | 1.3E-01 | -1.76 | 4.9E-03 | 0.83 | 3.7E-01 | Glucuronoxylan 4-O-methyltransferase 2 |
| Solyc06g071160.3.1 | LOC101247727 | -0.93 | 4.6E-04 | -0.23 | 4.1E-01 | -0.70 | 8.1E-03 | Galactosyltransferase family protein |
| Solyc04g078690.3.1 | LOC101259435 | -0.93 | 1.7E-01 | -1.09 | 6.4E-02 | 0.16 | 8.0E-01 | bHLH transcription factor 035 |
| Solyc01g079170.3.1 | LOC101261450 | -0.93 | 6.2E-02 | -0.89 | 9.1E-02 | -0.05 | 9.2E-01 | Hexosyltransferase |
| Solyc12g042360.3.1 | LOC101252560 | -0.94 | 1.8E-02 | -1.02 | 8.1E-03 | 0.07 | 9.2E-01 | Exostosin-like |
| Solyc01g067930.5.1 | LOC101262652 | -0.94 | 2.7E-07 | -0.70 | 9.7E-05 | -0.24 | 2.9E-01 | Xyloglucan 6-xylosyltransferase 1 |
| Solyc01g068460.4.1 | LOC101253931 | -0.94 | 2.1E-02 | -0.52 | 2.3E-01 | -0.43 | 4.4E-01 | Plant calmodulin-binding-like protein |
| Solyc09g063010.5.1 | LOC101264668 | -0.95 | 7.0E-03 | -0.50 | 1.6E-01 | -0.45 | 3.1E-01 | Basic helix-loop-helix (BHLH) DNA-binding superfamily protein |
| Solyc06g054260.1.1 | LOC543978 | -0.95 | 1.4E-02 | -0.56 | 1.7E-01 | -0.39 | 4.8E-01 | photosystem.1 |
| Solyc08g006930.3.1 | LOC101255222 | -0.95 | 3.4E-02 | -0.93 | 4.2E-02 | -0.03 | 9.6E-01 | photosystem I reaction center subunit psaK, chloroplastic |
| Solyc00g500050.1.1 | atpA | -0.95 | 1.7E-02 | -0.01 | 8.6E-01 | -0.94 | 6.8E-02 | ATP synthase subunit alpha, chloroplastic |
| Solyc03g114710.4.1 | LOC101263892 | -0.96 | 1.3E-02 | -0.72 | 7.1E-02 | -0.24 | 6.1E-01 | Glycosyltransferase |
| Solyc12g042600.2.1 | LOC101257246 | -0.97 | 4.1E-02 | -0.90 | 5.5E-02 | -0.07 | 9.2E-01 | Glycosyltransferase |
| Solyc09g064480.1.1 | LOC101245582 | -0.97 | 8.9E-03 | -0.86 | 1.9E-02 | -0.11 | 8.6E-01 | Xyloglucan galactosyltransferase KATAMAR.1 |
| Solyc01g073750.4.1 | LOC101254331 | -0.98 | 3.0E-04 | -0.63 | 2.3E-02 | -0.35 | 3.5E-01 | Callose synthase |
| Solyc08g079840.3.1 | P69A | -0.98 | 9.2E-02 | -0.59 | 3.0E-01 | -0.39 | 6.5E-01 | Subtilisin-like protease |
| Solyc03g113920.4.1 | LOC101256576 | -0.99 | 1.6E-02 | -1.14 | 4.3E-03 | 0.15 | 8.6E-01 | Calmodulin binding protein |
| Solyc04g010290.5.1 | LOC101265913 | -1.00 | 1.4E-02 | -0.42 | 3.1E-01 | -0.59 | 2.0E-01 | Core-2/I-branching beta-16-N-acetylglucosaminyltransferase family protein |
| Solyc08g083310.3.1 | LOC101260664 | -1.01 | 1.0E-01 | -1.11 | 6.0E-02 | 0.10 | 9.3E-01 | Glucan endo-1,3-beta-glucosidase .1 |
| Solyc01g096660.3.1 | LOC543930 | -1.01 | 1.8E-02 | -0.74 | 8.0E-02 | -0.27 | 6.9E-01 | proline-rich family protein |
| Solyc08g005780.4.1 | LOC101251494 | -1.01 | 8.4E-03 | -0.59 | 1.2E-01 | -0.42 | 3.4E-01 | Beta-amylase |
| Solyc03g019890.3.1 | TBG7 | -1.02 | 4.7E-02 | -1.41 | 1.3E-02 | 0.40 | 8.2E-01 | beta-galactosidase 7 |
| Solyc02g072150.3.1 | LOC101245612 | -1.02 | 3.7E-03 | -0.48 | 1.9E-01 | -0.54 | 2.2E-01 | Trehalose-6-phosphate synthase |
| Solyc08g074760.3.1 | LOC101257290 | -1.02 | 4.4E-04 | -1.02 | 3.4E-04 | 0.00 | 1.0E+00 | Receptor-like protein kinase |
| Solyc04g079030.2.1 |  | -1.02 | 3.4E-02 | -0.32 | 4.7E-01 | -0.70 | 2.4E-01 | Glycosyltransferase |
| Solyc08g079040.1.1 | LOC101248226 | -1.03 | 6.7E-03 | -0.52 | 1.7E-01 | -0.51 | 2.7E-01 | Xyloglucan galactosyltransferase KATAMAR.1 |
| Solyc00g500049.1.1 |  | -1.03 | 1.2E-01 | -1.06 | 7.2E-02 | 0.03 | 9.3E-01 | Photosystem II reaction center protein K |
| Solyc06g084480.3.1 | LOC101258381 | -1.03 | 1.0E-02 | -0.47 | 2.5E-01 | -0.56 | 2.2E-01 | protease Do-like 2, chloroplastic |
| Solyc04g054740.3.1 | IPS | -1.03 | 4.5E-03 | -0.39 | 8.1E-02 | -0.65 | 4.8E-01 | myo-inositol-1-phosphate synthase |
| Solyc02g080660.3.1 | LOC101250269 | -1.04 | 5.1E-02 | -0.70 | 2.3E-01 | -0.35 | 6.0E-01 | Glucan endo-1,3-beta-glucosidase.1 |
| Solyc02g089440.3.1 | LOC101246582 | -1.05 | 1.7E-04 | -0.93 | 6.7E-04 | -0.11 | 8.1E-01 | Hexosyltransferase |
| Solyc02g063523.1.1 | LOC112940012 | -1.05 | 9.2E-08 | -0.88 | 4.2E-06 | -0.16 | 5.5E-01 | Squalene epoxidase |
| Solyc10g008300.3.1 | LOC101264677 | -1.05 | 2.1E-03 | -0.91 | 6.3E-03 | -0.14 | 8.1E-01 | Mannan endo-1,4-beta-mannosidase |
| Solyc04g015270.3.1 | LOC101252577 | -1.05 | 5.5E-04 | -0.65 | 3.5E-02 | -0.40 | 3.2E-01 | Hexosyltransferase |
| Solyc06g082950.5.1 | LOC101265555 | -1.05 | 4.6E-02 | -0.91 | 7.9E-02 | -0.14 | 8.9E-01 | Photosystem I reaction center subunit XI protein |
| Solyc12g011450.2.1 | CAB13 | -1.05 | 2.7E-02 | -0.89 | 6.1E-02 | -0.16 | 8.6E-01 | Chlorophyll a-b binding protein, chloroplastic |
| Solyc02g084990.3.1 | LOC101265391 | -1.05 | 3.6E-03 | -0.35 | 2.2E-01 | -0.70 | 2.1E-01 | Mannan endo-1,4-beta-mannosidase |
| Solyc07g008900.4.1 | LOC101262630 | -1.05 | 2.7E-02 | -1.06 | 2.6E-02 | 0.00 | 9.9E-01 | Subtilisin-like protease-like protein |
| Solyc05g055440.1.1 | LOC101267024 | -1.05 | 1.9E-03 | -1.28 | 6.4E-05 | 0.23 | 5.9E-01 | Histone H2B |
| Solyc11g020610.3.1 | LOC101252138 | -1.07 | 1.6E-02 | -1.40 | 8.9E-04 | 0.34 | 5.9E-01 | Neutral/alkaline invertase |
| Solyc01g105050.3.1 | LHCP | -1.07 | 5.4E-02 | -1.44 | 6.9E-03 | 0.38 | 6.7E-01 | Chlorophyll a-b binding protein, chloroplastic |
| Solyc05g055280.2.1 | LOC101245166 | -1.07 | 5.7E-02 | -1.79 | 6.6E-04 | 0.73 | 2.3E-01 | Ferredoxin |
| Solyc09g014520.3.1 | LOC101249002 | -1.07 | 2.0E-02 | -1.09 | 1.5E-02 | 0.02 | 9.8E-01 | Chlorophyll a-b binding protein, chloroplastic |
| Solyc03g005760.1.1 | LOC101267774 | -1.07 | 3.4E-02 | -1.01 | 4.2E-02 | -0.06 | 9.6E-01 | Chlorophyll a-b binding protein, chloroplastic |
| Solyc08g080500.3.1 | LOC101268211 | -1.08 | 6.1E-03 | -1.33 | 3.4E-04 | 0.25 | 6.2E-01 | Rhomboid-like protein |
| Solyc09g009190.5.1 | LOC101268420 | -1.08 | 2.9E-06 | -0.97 | 1.6E-05 | -0.11 | 7.5E-01 | 1,4-alpha-glucan-branching enzyme 1, chloroplastic/amyloplastic |
| Solyc04g082010.1.1 | PETE | -1.08 | 7.9E-02 | -1.56 | 5.5E-03 | 0.48 | 5.5E-01 | pre-plastocyanin |
| Solyc07g055990.3.1 | LOC101255416 | -1.08 | 1.4E-01 | -1.85 | 8.5E-03 | 0.77 | 4.4E-01 | Xyloglucan endotransglucosylase/hydrolase |
| Solyc03g078100.3.1 | LOC101268168 | -1.08 | 2.7E-03 | -0.29 | 4.6E-01 | -0.79 | 2.4E-02 | Pectinesterase |
| Solyc02g070980.1.1 | Cab-1A | -1.08 | 1.3E-01 | -1.59 | 3.4E-02 | 0.51 | 7.6E-01 | Chlorophyll a-b binding protein, chloroplastic |
| Solyc10g044520.3.1 | SEND33 | -1.08 | 2.1E-02 | -1.15 | 1.3E-02 | 0.06 | 9.6E-01 | Ferredoxin |
| Solyc01g087560.3.1 | LOC101252433 | -1.09 | 2.3E-02 | -1.70 | 1.6E-04 | 0.62 | 2.5E-01 | Methyltransferase |
| Solyc07g042160.3.1 | LOC101248218 | -1.09 | 1.3E-02 | -1.05 | 1.4E-02 | -0.04 | 9.4E-01 | Pectin lyase-like superfamily protein |
| Solyc01g065530.3.1 | LOC101267739 | -1.09 | 1.1E-02 | -0.85 | 4.8E-02 | -0.24 | 7.4E-01 | COBRA-like protein |
| Solyc02g093230.4.1 | LOC101265977 | -1.10 | 3.0E-02 | -1.90 | 3.6E-05 | 0.80 | 5.7E-02 | Caffeoyl-CoA O-methyltransferase |
| Solyc06g063370.3.1 | CAB9 | -1.10 | 3.8E-02 | -1.32 | 1.2E-02 | 0.22 | 8.6E-01 | Chlorophyll a-b binding protein, chloroplastic |
| Solyc07g051820.3.1 | LOC101259456 | -1.11 | 3.1E-02 | -0.42 | 4.3E-01 | -0.68 | 3.1E-01 | Cellulose synthase |
| Solyc10g077040.2.1 | LOC101257518 | -1.11 | 4.6E-03 | -0.85 | 3.3E-02 | -0.26 | 6.6E-01 | Magnesium-protoporphyrin IX monomethyl ester [oxidative] cyclase, chloroplastic |
| Solyc11g069010.2.1 | LOC101249430 | -1.11 | 1.9E-04 | -1.27 | 8.4E-06 | 0.16 | 7.4E-01 | Alpha-L-fucosidase.1 |
| Solyc07g044960.1.1 | LOC101247341 | -1.12 | 1.1E-02 | -1.38 | 2.1E-03 | 0.26 | 8.3E-01 | Xyloglucan galactosyltransferase KATAMAR.1 |
| Solyc04g008720.4.1 | LOC101249258 | -1.13 | 4.0E-03 | -1.12 | 3.4E-03 | -0.01 | 9.7E-01 | Katanin p80 WD40 repeat-containing subunit .1 |
| Solyc11g066590.2.1 | LOC101266188 | -1.13 | 1.3E-01 | -0.96 | 4.5E-01 | -0.18 | 6.2E-01 | Serine carboxypeptidase S28 family protein |
| Solyc02g088000.3.1 | LOC101252941 | -1.14 | 5.1E-04 | -0.39 | 2.5E-01 | -0.74 | 4.6E-02 | Starch synthase, chloroplastic/amyloplastic |
| Solyc03g115900.5.1 | CAB11 | -1.14 | 1.8E-02 | -1.11 | 2.1E-02 | -0.03 | 9.7E-01 | Chlorophyll a-b binding protein, chloroplastic |
| Solyc06g083310.3.1 | LOC101250314 | -1.15 | 4.0E-04 | -1.06 | 9.0E-04 | -0.08 | 9.0E-01 | Hexosyltransferase |
| Solyc10g079490.2.1 | LOC101244432 | -1.15 | 3.2E-03 | -1.07 | 4.5E-03 | -0.07 | 8.9E-01 | Core-2/I-branching beta-16-N-acetylglucosaminyltransferase family protein |
| Solyc03g005780.3.1 | Cab-3C | -1.15 | 3.7E-02 | -1.49 | 1.3E-02 | 0.34 | 8.7E-01 | Chlorophyll a-b binding protein, chloroplastic |
| Solyc09g066280.4.1 | LOC101250441 | -1.16 | 4.6E-02 | -1.20 | 2.6E-02 | 0.05 | 9.4E-01 | Transcription factor bHLH155-like protein |
| Solyc07g005330.3.1 | LOC100191128 | -1.16 | 4.7E-03 | -1.25 | 1.5E-03 | 0.09 | 8.9E-01 | Glucan endo-1,3-beta-glucosidase |
| Solyc03g120640.3.1 | LOC101264609 | -1.16 | 3.8E-03 | -1.00 | 1.1E-02 | -0.16 | 8.4E-01 | Photosystem I reaction center subunit VI, chloroplastic |
| Solyc04g008730.3.1 | LOC101249542 | -1.16 | 5.0E-02 | -1.58 | 4.8E-03 | 0.41 | 6.2E-01 | Alpha-galactosidase |
| Solyc11g066680.1.1 | LOC101268547 | -1.17 | 6.1E-03 | -1.25 | 2.2E-03 | 0.08 | 9.2E-01 | Glycosyltransferase |
| Solyc11g062370.1.1 |  | -1.17 | 2.8E-03 | -1.01 | 9.6E-03 | -0.16 | 7.8E-01 | Stomatal closure-related actin-binding protein.1 |
| Solyc09g064500.3.1 | LOC101245880 | -1.17 | 1.8E-02 | -1.42 | 2.9E-03 | 0.25 | 7.6E-01 | Photosystem II reaction center Psb28 protein |
| Solyc04g077670.3.1 | LOC101252078 | -1.17 | 1.2E-02 | -1.34 | 3.0E-03 | 0.17 | 8.4E-01 | Serine carboxypeptidase-like 18 |
| Solyc03g115500.3.1 | LOC101258638 | -1.18 | 2.3E-03 | -1.29 | 5.2E-04 | 0.11 | 8.7E-01 | Heparanase-like protein.1 |
| Solyc01g102770.1.1 | psbZ | -1.18 | 5.3E-03 | 0.00 | 9.1E-01 | -1.18 | 2.3E-02 | Photosystem II reaction center protein Z |
| Solyc11g007920.1.1 | H2B-3 | -1.18 | 8.8E-03 | -1.83 | 1.5E-05 | 0.65 | 1.9E-01 | Histone H2B |
| Solyc09g064470.3.1 | LOC101245582 | -1.18 | 3.1E-04 | -0.88 | 6.6E-03 | -0.29 | 5.4E-01 | Xyloglucan galactosyltransferase KATAMAR.1 |
| Solyc03g113520.3.1 | LOC101264614 | -1.18 | 9.9E-04 | -1.11 | 1.5E-03 | -0.07 | 9.1E-01 | Galactosyltransferase family protein |
| Solyc07g044860.3.1 | PSBP | -1.18 | 7.2E-03 | -1.02 | 2.2E-02 | -0.17 | 8.4E-01 | psbXphotosystem II 23 kDa protein |
| Solyc02g068340.3.1 | LOC101267960 | -1.19 | 2.7E-03 | -1.52 | 9.5E-05 | 0.33 | 6.2E-01 | Kinesin-like protein |
| Solyc02g079040.3.1 | LOC101255950 | -1.19 | 8.3E-08 | -1.18 | 5.5E-08 | -0.01 | 9.9E-01 | Calmodulin binding protein-like |
| Solyc09g082710.3.1 | LOC101256806 | -1.20 | 9.9E-03 | -1.40 | 1.7E-03 | 0.20 | 8.0E-01 | Histone H2A |
| Solyc05g009880.3.1 | LOC101266819 | -1.20 | 1.4E-02 | -0.89 | 6.3E-02 | -0.31 | 6.6E-01 | bHLH transcription factor 038 |
| Solyc07g066310.3.1 | PSBR | -1.20 | 5.0E-03 | -0.58 | 1.7E-01 | -0.61 | 3.1E-01 | Photosystem II 10 kDa polypeptide, chloroplastic |
| Solyc03g114930.3.1 | LOC101259227 | -1.20 | 3.8E-02 | -1.25 | 2.6E-02 | 0.05 | 9.7E-01 | PsbP-like protein 1, chloroplastic |
| Solyc02g079950.3.1 | PsbQ | -1.20 | 2.3E-02 | -1.30 | 1.2E-02 | 0.10 | 9.4E-01 | photosystem II oxygen-evolving complex protein 3 |
| Solyc03g119250.5.1 | LOC101248759 | -1.21 | 2.6E-03 | -1.54 | 4.1E-05 | 0.34 | 5.1E-01 | Calmodulin binding protein-like |
| Solyc01g110340.5.1 | LOC101245995 | -1.21 | 3.2E-02 | -1.36 | 2.3E-02 | 0.15 | 9.8E-01 | Endoglucanase |
| Solyc04g077480.3.1 | LOC101251469 | -1.21 | 1.2E-03 | -1.22 | 8.0E-04 | 0.01 | 9.9E-01 | bHLH transcription factor 034 |
| Solyc03g005770.4.1 | LOC101245729 | -1.21 | 5.8E-03 | -1.45 | 2.3E-03 | 0.24 | 9.2E-01 | Chlorophyll a-b binding protein, chloroplastic |
| Solyc04g007910.3.1 | LOC101267699 | -1.21 | 1.0E-02 | -0.90 | 5.4E-02 | -0.32 | 5.9E-01 | Glucan endo-1,3-beta-glucosidase 3, putative, expressed |
| Solyc10g074920.2.1 | LOC101261972 | -1.22 | 3.7E-02 | -1.63 | 3.4E-03 | 0.41 | 6.3E-01 | Mannan endo-1,4-beta-mannosidase |
| Solyc01g110000.3.1 | LOC104645427 | -1.22 | 3.2E-02 | -1.49 | 1.1E-02 | 0.27 | 8.7E-01 | Beta-galactosidase |
| Solyc06g083680.3.1 | LOC101266426 | -1.22 | 2.8E-03 | -0.73 | 7.1E-02 | -0.49 | 3.9E-01 | Photosystem I reaction center subunit IV |
| Solyc12g099540.2.1 | LOC101260706 | -1.22 | 1.0E-02 | -1.22 | 7.8E-03 | 0.00 | 9.8E-01 | Kinesin-like protein |
| Solyc12g044280.2.1 | LOC101244751 | -1.22 | 1.8E-02 | -1.17 | 2.4E-02 | -0.05 | 9.5E-01 | Photosystem I reaction center subunit VI, chloroplastic |
| Solyc01g005080.3.1 | LOC101261948 | -1.22 | 3.0E-06 | -1.01 | 8.8E-05 | -0.21 | 5.4E-01 | 65-kDa microtubule-associated protein 6,Pfam:PF03999 |
| Solyc04g009740.4.1 | LOC101253679 | -1.22 | 4.2E-04 | -0.99 | 3.9E-03 | -0.23 | 6.3E-01 | Exocyst subunit Exo70 family protein |
| Solyc02g079250.4.1 | LOC101251056 | -1.22 | 2.3E-03 | -0.33 | 4.3E-01 | -0.89 | 3.8E-02 | Inositol monophosphatase family protein (3',5'-bisphosphate nucleotidase) |
| Solyc07g064970.4.1 | LOC101055514 | -1.23 | 1.2E-02 | -1.29 | 8.6E-03 | 0.06 | 9.8E-01 | 65-kDa microtubule-associated protein 1-like,Pfam:PF03999 |
| Solyc05g010685.1.1 | LOC101255991 | -1.23 | 1.2E-02 | -1.22 | 1.6E-02 | -0.01 | 9.0E-01 | Glycosyltransferase |
| Solyc04g070980.4.1 | CAS1 | -1.23 | 2.0E-02 | -0.61 | 2.4E-01 | -0.62 | 4.4E-01 | cycloartenol synthase 1 |
| Solyc10g011770.4.1 | LOC101260274 | -1.23 | 2.5E-03 | -1.13 | 5.1E-03 | -0.10 | 8.7E-01 | Protein CURVATURE THYLAKOID 1A, chloroplastic |
| Solyc12g017240.2.1 | tXET-B1 | -1.24 | 3.8E-03 | -1.14 | 7.2E-03 | -0.10 | 9.1E-01 | xyloglucan endo-transglycosylase B1 |
| Solyc01g087850.2.1 | sbt3 | -1.24 | 2.9E-02 | -1.87 | 5.9E-04 | 0.64 | 4.2E-01 | serine protease SBT3 |
| Solyc01g091920.2.1 | LOC101267161 | -1.25 | 4.2E-03 | -1.35 | 1.5E-03 | 0.11 | 9.6E-01 | Subtilisin-like protease-like protein |
| Solyc10g007690.3.1 | CAB-8 | -1.25 | 1.6E-03 | -0.69 | 6.9E-02 | -0.56 | 3.5E-01 | Chlorophyll a-b binding protein, chloroplastic |
| Solyc08g013670.3.1 | LOC101268297 | -1.25 | 1.3E-02 | -1.37 | 6.7E-03 | 0.12 | 9.4E-01 | Photosystem I reaction center subunit N, chloroplastic |
| Solyc03g071620.2.1 |  | -1.25 | 1.1E-02 | -1.70 | 2.9E-04 | 0.44 | 4.9E-01 | Histone H2B |
| Solyc10g054420.2.1 | LOC101256723 | -1.25 | 5.7E-03 | -0.37 | 4.0E-01 | -0.88 | 1.0E-01 | PsbP-like protein 2 |
| Solyc08g005050.4.1 | MYC1 | -1.26 | 2.3E-03 | -0.84 | 4.7E-02 | -0.41 | 4.4E-01 | transcription factor MYC2 |
| Solyc01g096670.5.1 | LOC101246092 | -1.26 | 5.0E-03 | -1.85 | 2.3E-05 | 0.59 | 3.2E-01 | Cytochrome P450 |
| Solyc05g009470.4.1 | LOC101256971 | -1.26 | 1.1E-02 | -1.58 | 1.3E-03 | 0.32 | 7.3E-01 | Glycoside hydrolase family .1 |
| Solyc02g071010.1.1 | CAB1B | -1.27 | 3.2E-02 | -1.62 | 9.2E-03 | 0.35 | 8.4E-01 | Chlorophyll a-b binding protein, chloroplastic |
| Solyc08g080050.4.1 | LOC101251312 | -1.27 | 2.3E-03 | -0.23 | 5.1E-01 | -1.04 | 4.3E-02 | PGR5-like protein 1A, chloroplastic |
| Solyc11g006020.2.1 | LOC101250560 | -1.27 | 1.2E-02 | -0.05 | 7.1E-01 | -1.22 | 5.8E-02 | NADH dehydrogenase-like complex O |
| Solyc12g056830.1.1 | LOC101254882 | -1.27 | 1.4E-03 | -0.74 | 6.5E-02 | -0.53 | 3.5E-01 | ATP synthase subunit delta, chloroplastic-like |
| Solyc04g007150.3.1 | LOC101248093 | -1.28 | 1.1E-03 | -1.84 | 7.8E-07 | 0.57 | 1.6E-01 | Glycoside hydrolase family .1 |
| Solyc01g088310.3.1 | LOC101266160 | -1.29 | 3.8E-04 | -1.12 | 1.4E-03 | -0.16 | 7.3E-01 | Geranylgeranyl diphosphate reductase, chloroplastic |
| Solyc04g007870.3.1 | LOC101243980 | -1.29 | 7.2E-09 | -0.99 | 6.9E-06 | -0.30 | 2.7E-01 | Myosin-binding protein.1 |
| Solyc09g075560.1.1 | LOC101262558 | -1.29 | 1.9E-02 | -1.29 | 1.3E-02 | 0.00 | 9.8E-01 | Hexosyltransferase |
| Solyc08g076930.1.1 | MYC2 | -1.29 | 2.5E-06 | -1.07 | 9.1E-05 | -0.23 | 6.0E-01 | jasmonic acid 3 |
| Solyc03g117970.4.1 | LOC101247901 | -1.29 | 6.2E-05 | -1.37 | 1.3E-05 | 0.07 | 9.2E-01 | Poly [ADP-ribose] polymerase |
| Solyc01g087780.2.1 | sbt4a | -1.29 | 6.9E-03 | -1.22 | 8.3E-03 | -0.08 | 9.6E-01 | serine protease SBT4A |
| Solyc09g010860.5.1 | EXPA4 | -1.30 | 2.3E-02 | -1.57 | 1.3E-02 | 0.27 | 9.6E-01 | Expansin |
| Solyc10g079070.2.1 | LOC101258908 | -1.30 | 3.6E-03 | -1.19 | 6.8E-03 | -0.12 | 8.8E-01 | bHLH transcription factor 065 |
| Solyc10g009210.4.1 | LOC101268806 | -1.31 | 1.0E-04 | -0.86 | 1.0E-02 | -0.45 | 2.8E-01 | Calmodulin binding protein-like |
| Solyc04g077440.4.1 | LOC101250012 | -1.32 | 8.7E-03 | -0.70 | 1.7E-01 | -0.62 | 3.9E-01 | Squalene epoxidase |
| Solyc07g063610.5.1 | LOC101267542 | -1.33 | 4.4E-03 | -0.98 | 3.1E-02 | -0.35 | 5.9E-01 | Dynein light chain |
| Solyc03g025730.3.1 | LOC101251552 | -1.33 | 2.0E-04 | -1.68 | 1.5E-06 | 0.35 | 5.0E-01 | Tubulin beta chain |
| Solyc04g007890.3.1 | LOC101268754 | -1.34 | 4.5E-04 | -1.33 | 3.0E-04 | -0.01 | 9.9E-01 | Hmg-y-related protein a |
| Solyc08g061100.3.1 | LOC101261539 | -1.35 | 2.2E-07 | -0.86 | 1.0E-03 | -0.49 | 1.5E-01 | Cellulose synthase |
| Solyc07g042580.3.1 | LOC101256016 | -1.35 | 2.3E-02 | -1.67 | 4.1E-03 | 0.32 | 7.6E-01 | microtubule-associated protein TORTIFOLI.1 |
| Solyc10g086760.2.1 | LOC101252240 | -1.36 | 1.2E-04 | -1.61 | 2.0E-06 | 0.26 | 6.0E-01 | Tubulin beta chain |
| Solyc03g120810.4.1 | LOC101260217 | -1.37 | 1.3E-03 | -1.32 | 1.7E-03 | -0.05 | 9.3E-01 | Kinesin-like protein |
| Solyc02g083810.4.1 | LOC101261284 | -1.37 | 9.4E-04 | -0.74 | 7.5E-02 | -0.63 | 2.7E-01 | Ferredoxin--NADP reductase, chloroplastic |
| Solyc11g073250.2.1 | LOC101265996 | -1.37 | 2.0E-03 | -2.27 | 5.8E-08 | 0.90 | 4.3E-02 | Histone H2A |
| Solyc10g054080.3.1 | LOC101259004 | -1.38 | 3.6E-03 | -1.24 | 8.7E-03 | -0.13 | 8.6E-01 | Kinesin-related protein .1 |
| Solyc03g111170.3.1 | LOC101266208 | -1.38 | 5.8E-05 | -1.36 | 4.2E-05 | -0.02 | 9.8E-01 | 4-coumarate-CoA ligase |
| Solyc05g006550.4.1 | LOC101247217 | -1.38 | 1.8E-02 | -1.20 | 3.5E-02 | -0.19 | 8.4E-01 | Microtubule-associated protein TORTIFOLI.1 |
| Solyc04g005050.1.1 | Sl3-MMP | -1.38 | 3.1E-04 | -1.66 | 4.9E-06 | 0.28 | 5.4E-01 | Metalloendoproteinase.1 |
| Solyc08g075120.3.1 | LOC101256509 | -1.39 | 1.3E-03 | -1.44 | 7.1E-04 | 0.05 | 1.0E+00 | Rop guanine nucleotide exchange factor 14 |
| Solyc02g080310.2.1 | LOC101257526 | -1.39 | 3.8E-03 | -0.89 | 6.0E-02 | -0.51 | 4.2E-01 | Beta-glucosidase 46 |
| Solyc07g061920.4.1 | LOC101252007 | -1.39 | 3.6E-03 | -1.48 | 1.6E-03 | 0.08 | 9.4E-01 | Callose synthase.1 |
| Solyc03g114720.3.1 | LOC101263596 | -1.40 | 4.0E-07 | -1.10 | 4.8E-05 | -0.30 | 3.6E-01 | bHLH transcription factor 023 |
| Solyc11g066270.3.1 | XTH6 | -1.40 | 3.3E-03 | -1.12 | 4.0E-02 | -0.28 | 5.0E-01 | xyloglucan endotransglucosylase-hydrolase 6 |
| Solyc06g075800.1.1 | LOC101253792 | -1.40 | 5.2E-03 | -2.26 | 1.7E-06 | 0.86 | 1.1E-01 | Histone H2B |
| Solyc09g090570.2.1 | LOC101262255 | -1.40 | 7.9E-04 | -0.42 | 3.4E-01 | -0.98 | 5.2E-02 | Proton gradient regulation 5 |
| Solyc03g098780.2.1 | LOC101262903 | -1.41 | 5.5E-02 | -0.12 | 7.1E-01 | -1.29 | 2.3E-01 | Aspartic protease inhibitor.1 |
| Solyc11g068440.2.1 | LOC101259605 | -1.41 | 8.2E-03 | -1.32 | 1.1E-02 | -0.10 | 9.5E-01 | Glucan endo-1,3-beta-glucosidase .1 |
| Solyc02g087350.3.1 | LOC101265190 | -1.42 | 2.0E-07 | -0.98 | 3.2E-04 | -0.43 | 2.1E-01 | Hexosyltransferase |
| Solyc06g082980.3.1 | LOC101259646 | -1.42 | 1.1E-02 | -1.21 | 2.9E-02 | -0.21 | 8.4E-01 | Emopamil-binding |
| Solyc06g060340.3.1 | psbS | -1.42 | 4.9E-03 | -1.16 | 2.1E-02 | -0.27 | 7.7E-01 | Photosystem II 22 kDa protein, chloroplastic |
| Solyc02g078950.4.1 | LOC101258612 | -1.42 | 2.3E-03 | -1.46 | 1.4E-03 | 0.03 | 1.0E+00 | Beta-galactosidase |
| Solyc04g015340.3.1 | LOC101250880 | -1.43 | 2.6E-04 | -1.38 | 2.8E-04 | -0.05 | 9.4E-01 | Carboxypeptidase |
| Solyc08g076650.3.1 | LOC101250326 | -1.43 | 1.7E-05 | -1.42 | 1.6E-05 | -0.02 | 9.6E-01 | Trehalose-6-phosphate synthase |
| Solyc07g055160.4.1 | LOC101245561 | -1.43 | 8.9E-04 | -0.95 | 2.6E-02 | -0.49 | 3.7E-01 | phospholipase A1-IIdelta-like |
| Solyc01g010270.3.1 | LOC101258583 | -1.44 | 6.4E-04 | -1.28 | 2.2E-03 | -0.16 | 8.2E-01 | Protein SPIRA.1 |
| Solyc08g082170.4.1 | LOC101258581 | -1.44 | 1.5E-03 | -1.38 | 1.8E-03 | -0.06 | 9.2E-01 | Pectin lyase-like superfamily protein |
| Solyc01g110290.3.1 | LOC100316899 | -1.45 | 1.3E-04 | -1.04 | 5.7E-03 | -0.41 | 4.5E-01 | Squalene synthase,Pfam:PF00494 |
| Solyc02g086180.4.1 | LOC101264777 | -1.45 | 3.6E-02 | -0.83 | 1.8E-01 | -0.62 | 6.3E-01 | Delta(7)-sterol-C5(6)-desaturase |
| Solyc02g065400.3.1 | PSBO | -1.46 | 2.5E-03 | -1.44 | 2.8E-03 | -0.02 | 9.7E-01 | 33kDa precursor protein of oxygen-evolving complex |
| Solyc07g022900.4.1 | LOC101257186 | -1.46 | 2.2E-02 | -1.29 | 3.8E-02 | -0.17 | 8.9E-01 | Chlorophyll a-b binding protein, chloroplastic |
| Solyc12g008580.2.1 | LOC101261689 | -1.47 | 2.2E-02 | -1.63 | 8.0E-03 | 0.16 | 8.8E-01 | Glucan endo-1,3-beta-glucosidase 12 |
| Solyc11g007770.2.1 | LOC101257919 | -1.47 | 2.4E-03 | -1.34 | 6.8E-03 | -0.13 | 8.2E-01 | Glycosyltransferase family protein 64 protein C5 |
| Solyc06g069580.3.1 | LOC101257181 | -1.47 | 4.0E-04 | -1.49 | 2.3E-04 | 0.02 | 9.9E-01 | Heparanase-like protein.1 |
| Solyc07g043160.1.1 | LOC101258278 | -1.48 | 3.2E-03 | 0.03 | 9.6E-01 | -1.51 | 5.3E-03 | Glycosyltransferase |
| Solyc12g098640.3.1 | CYC1 | -1.48 | 2.8E-03 | -1.60 | 7.5E-04 | 0.12 | 8.8E-01 | Cycloeucalenol cycloisomerase |
| Solyc07g062140.3.1 | LOC100134910 | -1.48 | 3.2E-03 | -1.57 | 1.3E-03 | 0.10 | 9.4E-01 | trehalose-phosphate synthase 1 |
| Solyc10g085020.3.1 | LOC101253952 | -1.48 | 7.4E-06 | -1.77 | 5.1E-08 | 0.29 | 5.8E-01 | Tubulin beta chain |
| Solyc10g078240.2.1 | LOC101262367 | -1.50 | 4.4E-05 | -1.12 | 1.9E-03 | -0.38 | 4.7E-01 | Cytochrome P450 |
| Solyc02g065530.5.1 | LOC101257137 | -1.50 | 1.8E-03 | -1.10 | 1.5E-02 | -0.41 | 5.6E-01 | Hexosyltransferase |
| Solyc05g056050.3.1 | LOC101253380 | -1.53 | 1.3E-03 | -1.13 | 1.7E-02 | -0.40 | 6.0E-01 | Chlorophyll a-b binding protein, chloroplastic |
| Solyc02g030170.4.1 | SlSSR1 | -1.53 | 2.3E-05 | -1.49 | 3.7E-05 | -0.04 | 9.5E-01 | DWARF1/DIMINUTO |
| Solyc06g069730.3.1 | CAB12 | -1.53 | 2.1E-02 | -1.62 | 1.5E-02 | 0.09 | 9.8E-01 | Chlorophyll a-b binding protein, chloroplastic |
| Solyc01g005330.3.1 | LOC101255723 | -1.54 | 1.3E-05 | -1.02 | 4.0E-03 | -0.52 | 2.5E-01 | Microtubule-associated protein 70 |
| Solyc07g047850.3.1 | CAB4 | -1.54 | 7.9E-04 | -1.50 | 1.1E-03 | -0.05 | 9.6E-01 | Chlorophyll a-b binding protein, chloroplastic |
| Solyc10g085220.3.1 |  | -1.54 | 1.7E-03 | -1.57 | 1.1E-03 | 0.02 | 9.7E-01 | Heparanase-like protein 3 |
| Solyc09g010400.3.1 | LOC101265669 | -1.55 | 7.7E-04 | -2.03 | 4.5E-06 | 0.48 | 3.6E-01 | Histone H2A |
| Solyc08g079870.3.1 | P69B | -1.55 | 4.5E-02 | -1.37 | 1.2E-01 | -0.19 | 8.0E-01 | subtilisin |
| Solyc01g102810.3.1 | LOC101254741 | -1.56 | 3.4E-04 | -1.43 | 8.0E-04 | -0.12 | 8.6E-01 | DNA polymerase III subunit gamma/tau |
| Solyc06g051260.4.1 | LOC101248685 | -1.56 | 8.4E-04 | -1.87 | 5.2E-05 | 0.31 | 7.2E-01 | bHLH transcription factor 043 |
| Solyc01g111930.4.1 | LOC101249771 | -1.57 | 5.1E-04 | -1.42 | 1.1E-03 | -0.14 | 8.6E-01 | Rop guanine nucleotide exchange factor.1 |
| Solyc04g054690.3.1 | AO | -1.57 | 7.5E-05 | -2.48 | 6.6E-11 | 0.91 | 4.2E-02 | ascorbate oxidase |
| Solyc05g012510.3.1 | LOC101248295 | -1.58 | 1.7E-04 | -1.22 | 3.4E-03 | -0.36 | 5.7E-01 | Alpha-1,4 glucan phosphorylase |
| Solyc09g082510.3.1 | LOC101252434 | -1.58 | 3.0E-04 | -1.06 | 1.6E-02 | -0.52 | 4.1E-01 | Kinase interacting (KIP1-like) family protein |
| Solyc07g052320.3.1 | LOC101254610 | -1.58 | 6.7E-04 | -1.47 | 1.2E-03 | -0.11 | 8.7E-01 | Hexosyltransferase |
| Solyc02g080540.1.1 | LOC101253342 | -1.58 | 2.4E-04 | -0.92 | 2.8E-02 | -0.66 | 2.8E-01 | ATP synthase gamma chain, chloroplastic |
| Solyc02g086830.3.1 | LOC101250650 | -1.58 | 4.6E-04 | -1.27 | 4.2E-03 | -0.32 | 6.3E-01 | Protease Do-like chloroplastic-like |
| Solyc03g093130.3.1 | XTH3 | -1.59 | 1.6E-03 | -1.71 | 3.9E-04 | 0.12 | 8.7E-01 | xyloglucan endotransglucosylase-hydrolase 3 |
| Solyc12g011280.2.1 | LOC101265617 | -1.59 | 1.0E-02 | -1.57 | 1.0E-02 | -0.02 | 9.8E-01 | Chlorophyll a-b binding protein, chloroplastic |
| Solyc03g114760.3.1 | LOC101055604 | -1.60 | 2.7E-03 | -1.97 | 1.4E-04 | 0.37 | 6.4E-01 | Protein MICROTUBULE BINDING PROTEIN 2C |
| Solyc09g075460.3.1 | LOC101264974 | -1.60 | 7.3E-04 | -1.52 | 1.5E-03 | -0.07 | 9.0E-01 | Pectin lyase-like superfamily protein |
| Solyc10g008120.5.1 | LOC101268615 | -1.60 | 1.2E-02 | -2.33 | 5.1E-04 | 0.74 | 5.7E-01 | O-methyltransferase |
| Solyc01g073970.3.1 | LOC101252717 | -1.60 | 8.9E-03 | -2.67 | 5.8E-06 | 1.08 | 1.1E-01 | Histone H3 |
| Solyc02g080220.3.1 | LOC101260787 | -1.61 | 1.7E-03 | -0.42 | 4.2E-01 | -1.19 | 3.9E-02 | Pectinesterase |
| Solyc02g070990.1.1 | CAB1B | -1.62 | 4.2E-02 | -1.76 | 2.5E-02 | 0.14 | 9.4E-01 | Chlorophyll a-b binding protein, chloroplastic |
| Solyc09g010980.1.1 |  | -1.62 | 1.1E-03 | -1.83 | 2.1E-04 | 0.21 | 8.6E-01 | cyclin-dependent kinase inhibitor |
| Solyc03g098290.4.1 | LOC101255377 | -1.62 | 7.5E-03 | -2.06 | 3.5E-04 | 0.45 | 5.6E-01 | Sucrose synthase |
| Solyc07g006680.1.1 | LOC101266953 | -1.62 | 3.7E-03 | -0.32 | 6.6E-01 | -1.30 | 2.6E-02 | HXXXD-type acyl-transferase family protein |
| Solyc01g006370.3.1 | LOC101263837 | -1.62 | 4.7E-04 | -1.54 | 8.2E-04 | -0.08 | 9.3E-01 | Callose synthase |
| Solyc06g074200.4.1 | LOC101254806 | -1.62 | 2.6E-03 | -1.59 | 3.1E-03 | -0.03 | 9.7E-01 | Photosystem I subunit O |
| Solyc10g009290.1.1 | LOC101267164 | -1.63 | 2.5E-03 | -1.95 | 1.8E-04 | 0.32 | 6.6E-01 | bHLH transcription factor155 |
| Solyc07g064670.3.1 | LOC101245676 | -1.63 | 4.7E-05 | -1.40 | 4.4E-04 | -0.23 | 7.1E-01 | Hexosyltransferase |
| Solyc12g056580.2.1 | LOC101260024 | -1.63 | 1.9E-06 | -1.12 | 1.1E-03 | -0.51 | 2.7E-01 | Cellulose synthase |
| Solyc04g072920.5.1 | LOC101249633 | -1.64 | 4.0E-03 | -2.27 | 3.9E-05 | 0.63 | 3.9E-01 | Trehalose 6-phosphate phosphatase |
| Solyc01g005830.4.1 | LOC101255913 | -1.64 | 1.4E-03 | -1.99 | 5.7E-05 | 0.35 | 5.9E-01 | Glucan endo-1,3-beta-glucosidase 4 |
| Solyc10g078340.2.1 | LOC101264685 | -1.64 | 1.0E-03 | -2.17 | 3.2E-05 | 0.53 | 6.3E-01 | Stomatal closure-related actin-binding protein 2 |
| Solyc06g083040.3.1 | LOC544223 | -1.64 | 1.1E-02 | -1.26 | 4.4E-02 | -0.39 | 7.1E-01 | wound-inducible carboxypeptidase |
| Solyc06g068880.3.1 | LOC101247625 | -1.64 | 3.5E-03 | -2.05 | 2.3E-04 | 0.41 | 6.7E-01 | Carboxypeptidase |
| Solyc04g008290.4.1 | LOC101259830 | -1.65 | 5.2E-06 | -0.95 | 9.0E-03 | -0.70 | 9.4E-02 | 65-kDa microtubule-associated protein 6,Pfam:PF03999 |
| Solyc02g070950.1.1 | Cab-1A | -1.65 | 3.7E-02 | -1.86 | 1.8E-02 | 0.21 | 9.2E-01 | Chlorophyll a-b binding protein, chloroplastic |
| Solyc04g080010.5.1 | LOC101251579 | -1.65 | 3.2E-04 | -0.93 | 3.6E-02 | -0.72 | 2.1E-01 | Glycosyltransferase |
| Solyc09g007920.4.1 | PAL3 | -1.65 | 1.3E-05 | -0.91 | 1.8E-02 | -0.74 | 1.3E-01 | Phenylalanine ammonia-lyase |
| Solyc08g006640.3.1 | LOC101246362 | -1.65 | 2.1E-04 | -1.66 | 1.5E-04 | 0.01 | 9.9E-01 | monogalactosyldiacylglycerol synthase, chloroplastic |
| Solyc09g097870.4.1 | LOC101268319 | -1.65 | 3.0E-04 | -1.54 | 6.4E-04 | -0.11 | 8.6E-01 | bHLH transcription factor 062 |
| Solyc01g010710.5.1 | LOC101251209 | -1.66 | 3.9E-03 | -0.88 | 1.2E-01 | -0.78 | NA | Carboxypeptidase |
| Solyc07g014640.3.1 | LOC101250610 | -1.66 | 2.3E-03 | -1.58 | 3.1E-03 | -0.08 | 9.1E-01 | Galactokinase |
| Solyc07g052230.3.1 | LOC101256306 | -1.66 | 1.0E-03 | -1.21 | 1.4E-02 | -0.44 | 5.5E-01 | L-ascorbate oxidase-like protein |
| Solyc02g091690.3.1 | LOC101251240 | -1.66 | 2.1E-03 | -1.68 | 1.2E-03 | 0.02 | 9.8E-01 | bHLH transcription factor 081 |
| Solyc11g069270.2.1 | TBG5 | -1.66 | 3.0E-04 | -1.30 | 4.3E-03 | -0.36 | 6.2E-01 | beta-galactosidase 5 |
| Solyc06g076640.3.1 | LOC101262728 | -1.67 | 1.2E-04 | -2.34 | 4.2E-08 | 0.67 | 2.5E-01 | Tubulin beta chain |
| Solyc03g116910.3.1 | CCR2 | -1.67 | 8.0E-03 | -1.80 | 5.8E-03 | 0.13 | 1.0E+00 | cinnamoyl-CoA reductase 2 |
| Solyc09g010060.3.1 | LOC101247848 | -1.67 | 1.7E-03 | -2.12 | 3.6E-05 | 0.45 | 5.1E-01 | Kinesin-related protein |
| Solyc08g083320.4.1 | GBSS1 | -1.67 | 3.2E-04 | -1.22 | 8.4E-03 | -0.45 | 5.3E-01 | Starch synthase, chloroplastic/amyloplastic |
| Solyc03g115070.1.1 | LOC101255283 | -1.67 | 3.0E-05 | -1.30 | 8.6E-04 | -0.37 | 5.4E-01 | Exocyst subunit Exo70 family protein |
| Solyc04g005560.3.1 | LOC101249543 | -1.68 | 5.3E-03 | -1.66 | 4.7E-03 | -0.02 | 9.8E-01 | ARF guanine-nucleotide exchange factor GNOM |
| Solyc04g016260.3.1 | LOC101262099 | -1.68 | 1.9E-06 | -1.58 | 4.1E-06 | -0.10 | 9.1E-01 | Calcium-transporting ATPase |
| Solyc06g074370.3.1 | LOC101250703 | -1.68 | 1.0E-03 | -1.56 | 1.6E-03 | -0.11 | 8.4E-01 | Sialyltransferase-like protein |
| Solyc08g060970.4.1 | PGcat | -1.68 | 6.7E-04 | -1.05 | 3.1E-02 | -0.63 | 2.8E-01 | polygalacturonase AF118567 |
| Solyc03g119980.3.1 | LOC101254287 | -1.68 | 4.5E-04 | -1.95 | 4.0E-05 | 0.27 | 7.8E-01 | Alpha/beta-Hydrolases superfamily protein |
| Solyc10g086340.1.1 | LOC101268334 | -1.68 | 4.3E-03 | -1.98 | 5.0E-04 | 0.29 | 7.3E-01 | Kinesin |
| Solyc03g044150.4.1 | LOC101245035 | -1.68 | 3.1E-04 | -1.90 | 3.7E-05 | 0.22 | 8.1E-01 | Subtilisin-like protease |
| Solyc07g053920.3.1 | LOC101245376 | -1.69 | 7.9E-05 | -1.70 | 4.6E-05 | 0.02 | 1.0E+00 | Mannan endo-1,4-beta-mannosidase |
| Solyc02g092450.3.1 | LOC101260388 | -1.69 | 4.0E-09 | -1.42 | 5.5E-07 | -0.28 | 5.6E-01 | Calcium-transporting ATPase |
| Solyc08g077530.4.1 | LOC101253815 | -1.70 | 8.1E-03 | -0.47 | 5.1E-01 | -1.23 | 1.0E-01 | Beta-amylase |
| Solyc07g041970.5.1 | LOC101251304 | -1.71 | 2.9E-04 | -2.28 | 1.1E-06 | 0.57 | 4.2E-01 | Subtilisin-like protease |
| Solyc02g081300.3.1 | LOC101266782 | -1.71 | 9.1E-03 | -2.14 | 7.5E-04 | 0.43 | 6.6E-01 | Sucrose synthase |
| Solyc02g070940.1.1 | LOC101263969 | -1.71 | 8.1E-04 | -1.74 | 8.3E-04 | 0.03 | 9.9E-01 | Chlorophyll a-b binding protein, chloroplastic |
| Solyc12g005050.2.1 | LOC101257536 | -1.71 | 5.9E-04 | -2.02 | 2.9E-05 | 0.31 | 6.6E-01 | AUGMIN subunit 4 |
| Solyc09g055930.3.1 | LOC101255529 | -1.72 | 1.5E-04 | -1.47 | 8.4E-04 | -0.24 | 7.0E-01 | monocopper oxidase-like protein SKU5 |
| Solyc12g014490.3.1 | LOC101263791 | -1.72 | 1.9E-07 | -1.68 | 1.4E-07 | -0.04 | 9.4E-01 | 65-kDa microtubule-associated protein 1-like,Pfam:PF03999 |
| Solyc07g049610.1.1 | LOC101263331 | -1.72 | 4.2E-06 | -1.50 | 5.9E-05 | -0.22 | 6.9E-01 | Xyloglucan galactosyltransferase KATAMAR.1 |
| Solyc01g109790.3.1 | AgpL1 | -1.72 | 2.5E-04 | -1.29 | 7.0E-03 | -0.43 | 5.3E-01 | ADP-glucose pyrophosphorylase large subunit 1 |
| Solyc10g006230.4.1 | CAB7 | -1.72 | 4.0E-05 | -1.14 | 5.7E-03 | -0.58 | 3.5E-01 | Chlorophyll a-b binding protein, chloroplastic |
| Solyc10g075160.1.1 | LOC101265784 | -1.73 | 1.8E-03 | -1.17 | 3.6E-02 | -0.56 | 4.7E-01 | Ferredoxin |
| Solyc12g098630.2.1 | LOC101262096 | -1.73 | 1.9E-03 | -2.16 | 6.3E-05 | 0.43 | 5.7E-01 | Kinesin-like protein KIN12B |
| Solyc09g007900.5.1 | LOC101243631 | -1.73 | 2.6E-05 | -1.33 | 1.2E-03 | -0.40 | 5.4E-01 | Phenylalanine ammonia-lyase |
| Solyc06g082940.3.1 | LOC101265249 | -1.73 | 2.6E-04 | -1.61 | 7.5E-04 | -0.12 | 8.7E-01 | Photosystem I reaction center subunit XI protein |
| Solyc03g093120.5.1.1 | LOC101258926 | -1.74 | 4.3E-03 | -2.09 | 2.6E-04 | 0.35 | 6.5E-01 | Xyloglucan endotransglucosylase/hydrolase |
| Solyc04g079050.3.1 | LOC101256480 | -1.74 | 1.9E-05 | -0.82 | 3.6E-02 | -0.92 | 6.0E-02 | UDP-glycosyltransferase 79B2 |
| Solyc03g111380.3.1 | LOC101260712 | -1.74 | 9.3E-05 | -2.14 | 9.2E-07 | 0.40 | 5.5E-01 | Tubulin gamma chain |
| Solyc06g073320.3.1 | GGP | -1.74 | 1.1E-04 | -0.77 | 8.4E-02 | -0.97 | 9.8E-02 | GDP-L-galactose phosphorylase.1 |
| Solyc01g091480.5.1 | LOC101255832 | -1.74 | 5.8E-06 | -1.69 | 6.3E-06 | -0.05 | 9.4E-01 | Kinesin-like protein |
| Solyc06g083070.3.1 | LOC101257576 | -1.75 | 1.7E-05 | -1.29 | 6.5E-04 | -0.46 | 5.5E-01 | Fimbrin-2 |
| Solyc12g094520.2.1 | LOC101251665 | -1.75 | 7.1E-08 | -1.78 | 2.3E-08 | 0.03 | 9.6E-01 | 4-coumarate--CoA ligase-like 4 |
| Solyc07g008390.4.1 | LOC104648161 | -1.76 | 5.0E-02 | -0.50 | 4.2E-01 | -1.26 | 4.3E-01 | HXXXD-type acyl-transferase family protein |
| Solyc12g099160.2.1 | LOC101244564 | -1.77 | 8.5E-04 | -2.66 | 2.5E-07 | 0.89 | 2.7E-02 | Carboxypeptidase |
| Solyc01g006300.3.1 | CEVI-1 | -1.78 | 1.0E-03 | -0.55 | 2.5E-01 | -1.23 | 9.4E-02 | LECEVI1A |
| Solyc06g076630.3.1 | LOC101263035 | -1.78 | 5.2E-03 | -2.23 | 4.1E-04 | 0.45 | 6.9E-01 | Peroxidase |
| Solyc02g088690.5.1 | LOC101263958 | -1.79 | 1.3E-05 | -1.82 | 6.2E-06 | 0.03 | 9.6E-01 | UDP-glucose 6-dehydrogenase |
| Solyc02g092840.1.1 | LOC101251138 | -1.79 | 1.2E-08 | -1.06 | 6.0E-04 | -0.73 | 3.2E-02 | Xyloglucan galactosyltransferase KATAMAR.1 |
| Solyc02g081730.3.1 | LOC101256443 | -1.80 | 2.8E-03 | -1.53 | 9.1E-03 | -0.26 | 8.1E-01 | 3beta-hydroxysteroid-dehydrogenase/decarboxylase isoform.1 |
| Solyc10g008520.3.1 | LOC101258495 | -1.80 | 4.9E-05 | -1.06 | 1.2E-02 | -0.74 | 2.2E-01 | Auxin-responsive GH3 family protein |
| Solyc03g116500.4.1 | XOPG1 | -1.80 | 3.9E-04 | -1.99 | 9.0E-05 | 0.18 | 9.1E-01 | polygalacturonase (XOPG1) |
| Solyc01g109570.4.1 | LOC101246571 | -1.81 | 1.1E-06 | -1.78 | 1.2E-06 | -0.03 | 9.6E-01 | Glucan endo-1,3-beta-glucosidase |
| Solyc01g086820.5.1 |  | -1.81 | 2.3E-02 | -2.74 | 2.1E-04 | 0.93 | 2.7E-01 | Histone H3 |
| Solyc02g077480.1.1 | LOC101267861 | -1.81 | 1.4E-02 | -2.70 | 8.1E-05 | 0.89 | 2.8E-01 | Histone H3 |
| Solyc03g081260.4.1 | LOC101249996 | -1.81 | 2.3E-06 | -2.23 | 2.4E-09 | 0.42 | 4.6E-01 | Subtilisin-like protease SBT3.5 |
| Solyc04g081490.3.1 | TUB | -1.81 | 2.8E-06 | -1.90 | 6.4E-07 | 0.09 | 9.1E-01 | beta-tubulin |
| Solyc06g083030.4.1 | LOC101258166 | -1.82 | 2.5E-04 | -1.78 | 2.1E-04 | -0.04 | 9.6E-01 | Carboxypeptidase |
| Solyc01g087970.3.1 | LOC101260269 | -1.82 | 5.4E-03 | -1.19 | 7.5E-02 | -0.63 | NA | Carboxypeptidase |
| Solyc02g065765.1.1 | COBRA-like | -1.83 | 4.9E-05 | -1.49 | 7.4E-04 | -0.33 | 6.7E-01 | COBRA-like protein |
| Solyc04g016200.1.1 | LOC101263304 | -1.83 | 2.0E-03 | -0.80 | 1.9E-01 | -1.03 | 1.0E-01 | Glycosyltransferase |
| Solyc08g079710.3.1 | LOC101258682 | -1.83 | 1.9E-07 | -1.66 | 1.4E-06 | -0.17 | 7.3E-01 | Kinesin-like protein |
| Solyc04g008330.1.1 | LOC101258941 | -1.84 | 1.9E-03 | -1.88 | 1.4E-03 | 0.04 | 9.9E-01 | Glycosyltransferase |
| Solyc12g007230.2.1 | SlIAA8 | -1.84 | 8.8E-05 | -2.04 | 1.1E-05 | 0.20 | 8.5E-01 | auxin-regulated IAA8 |
| Solyc09g014380.3.1 | SlLAX1 | -1.84 | 4.3E-05 | -1.76 | 8.3E-05 | -0.09 | 9.0E-01 | SlLAX1 |
| Solyc02g093430.3.1 | LOC101260885 | -1.85 | 2.3E-08 | -1.32 | 5.4E-05 | -0.53 | 2.0E-01 | Beta-1,4-N-acetylglucosaminyltransferase family protein |
| Solyc04g077190.4.1 |  | -1.85 | 6.7E-03 | -2.00 | 2.8E-03 | 0.15 | 9.3E-01 | Endo-1,4-beta-xylanase.1 |
| Solyc12g036170.2.1 | LOC101254767 | -1.86 | 8.6E-04 | -0.74 | 1.5E-01 | -1.12 | 1.0E-01 | Photosynthetic NDH subcomplex B 4 |
| Solyc03g114970.3.1 | LOC101257849 | -1.87 | 2.2E-03 | -2.46 | 3.1E-05 | 0.59 | 4.2E-01 | Protein SPIRA.1 |
| Solyc05g006650.3.1 | LOC101248972 | -1.87 | 1.7E-04 | -1.40 | 4.0E-03 | -0.47 | 5.0E-01 | bHLH transcription factor 036 |
| Solyc06g065990.1.1 | LOC101263124 | -1.88 | 1.4E-04 | -0.92 | 6.0E-02 | -0.95 | 6.7E-02 | ATP synthase subunit b', chloroplastic |
| Solyc09g061860.4.1 | LOC101263151 | -1.88 | 1.5E-03 | -1.44 | 1.4E-02 | -0.45 | 6.3E-01 | Sterol 3-beta-glucosyltransferase |
| Solyc01g079790.5.1 | agpL3 | -1.89 | 3.1E-05 | -1.37 | 2.1E-03 | -0.51 | 4.1E-01 | Glucose-1-phosphate adenylyltransferase |
| Solyc11g066720.3.1 | LOC101244155 | -1.89 | 1.2E-06 | -1.14 | 2.7E-03 | -0.75 | 1.4E-01 | UDP-apiose/UDP-xylose synthase |
| Solyc12g011290.2.1 | LOC101255070 | -1.89 | 6.9E-04 | -2.00 | 2.7E-04 | 0.11 | 9.5E-01 | Kinesin-4 |
| Solyc04g072850.5.1 | SlArf/Xyl4 | -1.90 | 2.9E-05 | -1.84 | 3.9E-05 | -0.06 | 9.4E-01 | Beta-D-xylosidase |
| Solyc05g053400.3.1 | LOC101248301 | -1.90 | 1.6E-04 | -1.54 | 1.8E-03 | -0.36 | 6.3E-01 | Glycosyltransferase |
| Solyc12g009200.2.1 | LOC101252151 | -1.91 | 5.2E-03 | -0.78 | 2.4E-01 | -1.13 | 1.7E-01 | Chlorophyll a-b binding protein, chloroplastic |
| Solyc02g089065.1.1 | LOC101256145 | -1.91 | 3.5E-03 | -2.07 | 1.2E-03 | 0.17 | 9.1E-01 | Protein SPIRA.1 |
| Solyc06g075930.1.1 | LOC101252889 | -1.92 | 4.4E-03 | -2.21 | 6.3E-04 | 0.29 | 7.4E-01 | Histone H4 |
| Solyc07g063600.3.1 | LOC101268123 | -1.92 | 1.2E-03 | -1.89 | 1.2E-03 | -0.03 | 9.8E-01 | Chlorophyll a-b binding protein, chloroplastic |
| Solyc03g020040.3.1 | LOC109119847 | -1.92 | 7.6E-03 | -1.39 | 4.2E-02 | -0.53 | 6.6E-01 | Proteinase inhibitor type-2 |
| Solyc07g007620.3.1 | LOC101259856 | -1.92 | 1.6E-04 | -1.48 | 2.8E-03 | -0.45 | 4.8E-01 | Monogalactosyldiacylglycerol synthase 3, chloroplastic |
| Solyc02g065170.3.1 | LOC101263571 | -1.93 | 1.2E-03 | -2.30 | 2.2E-04 | 0.37 | 8.5E-01 | L-ascorbate oxidase-like protein |
| Solyc03g083960.3.1 | LOC101246803 | -1.94 | 2.6E-05 | -2.02 | 9.4E-06 | 0.09 | 9.7E-01 | Trehalose 6-phosphate phosphatase |
| Solyc11g066670.1.1 | LOC101268254 | -1.94 | 2.6E-04 | -1.45 | 5.0E-03 | -0.48 | 5.8E-01 | Glycosyltransferase |
| Solyc06g072220.1.1 | LOC101248784 | -1.94 | 4.2E-03 | -1.09 | 9.5E-02 | -0.85 | 3.8E-01 | miraculin-like |
| Solyc04g078610.4.1 | LOC101247910 | -1.94 | 2.1E-06 | -1.53 | 1.8E-04 | -0.41 | 4.7E-01 | Kinesin |
| Solyc01g080280.3.1 | GS2 | -1.94 | 1.1E-05 | -1.36 | 1.9E-03 | -0.58 | 3.6E-01 | chloroplast glutamine synthetase |
| Solyc01g009610.3.1 | LOC101243734 | -1.95 | 2.0E-04 | -1.66 | 1.1E-03 | -0.29 | 6.5E-01 | Protein phosphatase inhibitor 2-like |
| Solyc09g009040.3.1 | FK | -1.95 | 1.0E-03 | -1.35 | 2.0E-02 | -0.60 | 4.9E-01 | Delta(14)-sterol reductase |
| Solyc02g087190.1.1 | LOC101244246 | -1.95 | 5.0E-06 | -1.84 | 1.3E-05 | -0.11 | 8.8E-01 | Peroxidase |
| Solyc06g075830.2.1 | LOC101252889 | -1.96 | 1.1E-03 | -2.57 | 8.7E-06 | 0.61 | 3.0E-01 | Histone H4 |
| Solyc03g115980.1.1 | LOC101262299 | -1.97 | 1.2E-05 | -1.69 | 2.0E-04 | -0.28 | 6.8E-01 | Geranylgeranyl diphosphate reductase, chloroplastic |
| Solyc01g087210.3.1 | LOC101246863 | -1.97 | 1.8E-07 | -1.54 | 4.4E-05 | -0.43 | 4.4E-01 | Cellulose synthase |
| Solyc10g084320.3.1 | LOC101252447 | -1.97 | 2.7E-04 | -2.45 | 1.3E-05 | 0.48 | 7.1E-01 | Subtilisin-like protease |
| Solyc11g071730.3.1 | LOC101260697 | -1.97 | 2.8E-03 | -2.43 | 1.7E-04 | 0.46 | 6.3E-01 | Phragmoplast orienting kinesin.1 |
| Solyc02g069450.3.1 | LOC101245421 | -1.98 | 6.1E-04 | -2.09 | 2.6E-04 | 0.11 | 9.5E-01 | Photosystem I reaction center subunit III, chloroplastic |
| Solyc06g084050.4.1 | LOC101257676 | -2.00 | 4.8E-04 | -1.81 | 1.2E-03 | -0.18 | 8.5E-01 | Photosystem II reaction center W protein, chloroplastic |
| Solyc11g040340.3.1 | cel7 | -2.00 | 2.1E-04 | -2.47 | 2.2E-06 | 0.48 | 5.2E-01 | LEE14BDGL L.esculentum endo-1,4-beta-D-glucanase |
| Solyc01g091320.3.1 | LOC104649012 | -2.00 | 2.5E-03 | -1.74 | 6.3E-03 | -0.26 | 8.8E-01 | Methylsterol monooxygenase 1-2 |
| Solyc07g055930.3.1 | LOC101256305 | -2.01 | 8.0E-08 | -1.40 | 1.5E-04 | -0.61 | 1.8E-01 | Hexosyltransferase |
| Solyc09g065850.4.1 | IAA3 | -2.01 | 5.2E-07 | -1.67 | 3.3E-05 | -0.34 | 5.6E-01 | auxin-regulated IAA3 |
| Solyc07g052510.4.1 | LOC101250523 | -2.01 | 6.3E-03 | -2.79 | 4.2E-04 | 0.78 | 6.6E-01 | peroxidase (TPX1) |
| Solyc09g057630.3.1 | LOC101263456 | -2.02 | 5.3E-05 | -1.95 | 5.7E-05 | -0.07 | 9.4E-01 | Glucan endo-1,3-beta-glucosidase 3-like protein |
| Solyc03g093080.3.1 | LOC101258345 | -2.02 | 2.8E-05 | -2.47 | 1.2E-07 | 0.45 | 5.0E-01 | Xyloglucan endotransglucosylase/hydrolase |
| Solyc09g066100.3.1 | LOC101248710 | -2.02 | 4.4E-04 | -2.46 | 1.2E-05 | 0.44 | 6.1E-01 | Histone .1 |
| Solyc03g097050.3.1 | LOC101247596 | -2.03 | 2.2E-08 | -1.92 | 7.5E-08 | -0.11 | 9.1E-01 | Cellulose synthase |
| Solyc02g089730.1.1 | LOC101265693 | -2.03 | 9.8E-06 | -2.25 | 5.3E-07 | 0.21 | 6.9E-01 | Endo-1,3(4)-beta-glucanase.1 |
| Solyc03g019790.3.1 | AGAL | -2.03 | 1.9E-05 | -1.42 | 1.1E-03 | -0.61 | 4.4E-01 | alpha-galactosidase |
| Solyc03g123630.4.1 | PMEU1 | -2.04 | 1.5E-08 | -1.95 | 3.5E-08 | -0.09 | 9.1E-01 | pectin methylesterase pmeu1 |
| Solyc01g103960.3.1 | LOC101260976 | -2.04 | 6.9E-05 | -2.29 | 4.5E-06 | 0.25 | 7.3E-01 | RNA helicase DEAH-box15 |
| Solyc09g082660.3.1 | AnthOMT | -2.04 | 1.1E-04 | -2.37 | 1.9E-05 | 0.33 | 8.8E-01 | Caffeoyl-CoA O-methyltransferase |
| Solyc02g087880.3.1 | LOC101255154 | -2.05 | 3.1E-03 | -2.09 | 1.5E-03 | 0.03 | 9.5E-01 | Tubulin alpha chain |
| Solyc02g078130.3.1 | LOC101247399 | -2.06 | 4.1E-04 | -3.33 | 1.9E-09 | 1.27 | 8.6E-03 | bHLH transcription factor 079 |
| Solyc01g096370.4.1 | LOC101264068 | -2.06 | 8.1E-06 | -1.37 | 2.6E-03 | -0.69 | 2.0E-01 | transcription factor MYC2 |
| Solyc02g069630.3.1 | LOC101266183 | -2.06 | 1.6E-04 | -2.28 | 2.5E-05 | 0.22 | 8.7E-01 | Subtilisin-like protease |
| Solyc07g043500.1.1 | LOC101254109 | -2.06 | 3.4E-06 | -1.14 | 9.7E-03 | -0.93 | 9.8E-02 | Glycosyltransferase |
| Solyc07g056000.2.1 | tXET-B2 | -2.07 | 6.9E-07 | -1.76 | 2.0E-05 | -0.31 | 6.6E-01 | Xyloglucan endotransglucosylase/hydrolase |
| Solyc06g074090.3.1 | LOC101256596 | -2.07 | 2.4E-04 | -1.54 | 5.7E-03 | -0.53 | 5.6E-01 | 7-dehydrocholesterol reductase |
| Solyc02g082180.3.1 | LOC101244828 | -2.08 | 8.2E-05 | -2.30 | 7.7E-06 | 0.23 | 8.0E-01 | DNA helicase |
| Solyc05g053120.1.1 | LOC101248301 | -2.08 | 1.9E-04 | -1.37 | 7.8E-03 | -0.71 | 3.4E-01 | Glycosyltransferase |
| Solyc02g093300.3.1 | DNApolalpha | -2.08 | 9.2E-04 | -2.44 | 8.5E-05 | 0.36 | 7.5E-01 | DNA polymerase |
| Solyc04g082110.5.1 | LOC101251179 | -2.09 | 1.2E-04 | -2.02 | 1.3E-04 | -0.06 | 9.5E-01 | Rop guanine nucleotide exchange factor 1 |
| Solyc11g066430.3.1 | H2B-2 | -2.09 | 3.5E-04 | -3.13 | 4.6E-08 | 1.04 | 1.1E-01 | Histone H2B |
| Solyc03g115220.5.1 | LOC101266618 | -2.09 | 2.6E-04 | -0.50 | 2.6E-01 | -1.59 | 3.7E-02 | Flavonoid 3'-monooxygenase |
| Solyc10g045240.2.1 | LOC101254849 | -2.09 | 9.1E-04 | -2.12 | 5.3E-05 | 0.03 | 7.1E-01 | Beta-glucosidase |
| Solyc12g009270.1.1 | LOC101254166 | -2.10 | 7.4E-04 | -1.86 | 1.7E-03 | -0.24 | 8.7E-01 | Plant invertase/pectin methylesterase inhibitor superfamily protein |
| Solyc03g123490.1.1 | LOC101249160 | -2.10 | 2.2E-07 | -1.94 | 1.5E-06 | -0.16 | 8.0E-01 | Subtilisin-like protease-like protein |
| Solyc02g083490.3.1 | LOC101267473 | -2.11 | 1.8E-03 | -2.99 | 3.9E-05 | 0.88 | 5.5E-01 | Peroxidase |
| Solyc02g091920.3.1 | XTH7 | -2.12 | 4.6E-05 | -1.69 | 2.8E-04 | -0.43 | 7.7E-01 | xyloglucan endotransglucosylase-hydrolase 7 |
| Solyc05g007830.3.1 | LOC543795 | -2.12 | 5.6E-04 | -2.14 | 3.2E-04 | 0.01 | 9.9E-01 | expansin12 |
| Solyc11g013110.2.1 | LOC101249699 | -2.13 | 2.1E-05 | -0.37 | 2.9E-01 | -1.76 | 7.2E-03 | 2-oxoglutarate (2OG) and Fe(II)-dependent oxygenase superfamily protein |
| Solyc02g088820.4.1 | LOC101260592 | -2.13 | 4.6E-05 | -1.66 | 1.3E-03 | -0.48 | 4.9E-01 | Carboxypeptidase |
| Solyc04g011390.1.1 | LOC544081 | -2.14 | 1.4E-03 | -2.60 | 8.5E-05 | 0.46 | 6.8E-01 | Histone H4 |
| Solyc02g080290.3.1 | LOC104645804 | -2.14 | 8.6E-06 | -1.31 | 5.3E-03 | -0.83 | 7.4E-02 | Beta-glucosidase 46 |
| Solyc03g093110.3.1 | LOC101258632 | -2.14 | 4.2E-06 | -2.15 | 3.3E-06 | 0.01 | 1.0E+00 | Xyloglucan endotransglucosylase/hydrolase |
| Solyc07g042170.3.1 | LOC101247936 | -2.15 | 3.9E-09 | -2.29 | 1.0E-10 | 0.14 | 7.9E-01 | Jasmonate ZIM-domain protein 3 |
| Solyc09g010210.3.1 | Cel2 | -2.16 | 3.3E-07 | -2.09 | 3.6E-07 | -0.07 | 8.8E-01 | endo-1,4-beta-glucanase precursor (Cel2) |
| Solyc06g062580.3.1 | LOC101258163 | -2.16 | 3.9E-04 | -2.28 | 1.8E-04 | 0.12 | 9.6E-01 | Beta-galactosidase |
| Solyc02g063010.3.1 | LOC101260897 | -2.16 | 9.0E-13 | -2.00 | 1.2E-11 | -0.16 | 6.7E-01 | BES1/BZR1-like protein |
| Solyc02g080670.3.1 | LOC101249981 | -2.17 | 4.3E-04 | -2.49 | 7.1E-05 | 0.32 | 8.6E-01 | L-ascorbate oxidase-like protein |
| Solyc07g008380.2.1 | LOC104648251 | -2.17 | 3.3E-02 | -1.00 | 2.8E-01 | -1.16 | 4.8E-01 | HXXXD-type acyl-transferase family protein |
| Solyc07g056140.3.1 | LOC543968 | -2.17 | 4.1E-09 | -1.94 | 1.4E-07 | -0.23 | 6.8E-01 | ADP-glucose pyrophosphorylase small subunit |
| Solyc09g065100.3.1 | LOC101254527 | -2.18 | 3.4E-04 | -1.69 | 5.1E-03 | -0.48 | 4.9E-01 | bHLH transcription factor 150 |
| Solyc11g062100.1.1 | LOC101255059 | -2.18 | 4.9E-05 | -2.54 | 1.3E-06 | 0.36 | 4.8E-01 | E1-E2_ATPase domain-containing protein/HMA domain-containing protein/Hydrolase domain-containing protein |
| Solyc02g090360.3.1 | LOC101252344 | -2.18 | 3.9E-06 | -2.29 | 6.7E-07 | 0.11 | 8.9E-01 | L-ascorbate oxidase-like protein |
| Solyc06g053830.3.1 | IAA7 | -2.18 | 5.3E-05 | -1.52 | 3.5E-03 | -0.66 | 3.1E-01 | auxin-regulated IAA7 |
| Solyc03g111690.4.1 | SlPL | -2.18 | 1.9E-05 | -1.59 | 1.9E-03 | -0.59 | 3.3E-01 | Pectate lyase |
| Solyc05g005080.3.1 | LOC101244370 | -2.19 | 1.0E-06 | -1.80 | 4.7E-05 | -0.39 | 6.0E-01 | Endoglucanase |
| Solyc02g070780.5.1 | LOC101268250 | -2.19 | 1.9E-04 | -2.67 | 3.8E-06 | 0.48 | 5.9E-01 | DNA helicase |
| Solyc04g081300.4.1 | LOC101259149 | -2.19 | 3.3E-05 | -1.64 | 1.0E-03 | -0.55 | 5.6E-01 | Endoglucanase |
| Solyc09g007890.1.1 | LOC101243631 | -2.20 | 7.6E-05 | -1.85 | 5.7E-04 | -0.35 | 7.5E-01 | Phenylalanine ammonia-lyase |
| Solyc11g071640.3.1 | LOC101256554 | -2.20 | 4.1E-07 | -2.52 | 3.0E-09 | 0.32 | 6.5E-01 | Glycosyl hydrolase family protein |
| Solyc12g006470.2.1 | GABA-TP2 | -2.20 | 1.1E-05 | -1.54 | 8.9E-04 | -0.66 | 4.8E-01 | viroid RNA-binding protein |
| Solyc11g040120.3.1 | LOC101260396 | -2.20 | 1.9E-06 | -2.35 | 3.5E-07 | 0.15 | 9.2E-01 | DNA helicase |
| Solyc07g043480.1.1 | LOC101254402 | -2.21 | 1.9E-04 | -1.96 | 6.4E-04 | -0.24 | 8.6E-01 | Glycosyltransferase |
| Solyc08g082250.3.1 | Cel8 | -2.21 | 9.7E-07 | -2.17 | 1.4E-06 | -0.05 | 9.4E-01 | endo-beta-1,4-D-glucanase (Cel8) |
| Solyc11g072110.2.1 | LOC101266599 | -2.22 | 5.3E-07 | -1.39 | 7.6E-04 | -0.83 | 2.1E-01 | 2-oxoglutarate (2OG) and Fe(II)-dependent oxygenase superfamily protein |
| Solyc03g119220.3.1 | LOC101263097 | -2.23 | 4.6E-04 | -2.51 | 5.8E-05 | 0.28 | 8.0E-01 | Kinesin-like protein |
| Solyc02g069690.1.1 | LOC101265606 | -2.23 | 1.2E-05 | -2.81 | 1.9E-08 | 0.58 | 3.6E-01 | FAD-binding Berberine family protein |
| Solyc03g113980.3.1 |  | -2.23 | 1.2E-07 | -1.72 | 4.0E-05 | -0.51 | 3.3E-01 | Calmodulin binding protein-like |
| Solyc12g008490.2.1 | LOC101259712 | -2.24 | 3.2E-04 | -1.97 | 1.1E-03 | -0.27 | 8.0E-01 | Nucleotide-diphospho-sugar transferases superfamily protein |
| Solyc05g013440.3.1 | LOC101266123 | -2.24 | 3.5E-07 | -1.46 | 7.7E-04 | -0.78 | 1.8E-01 | Amine oxidase |
| Solyc09g010810.3.1 | LOC101255427 | -2.24 | 1.9E-04 | -2.95 | 6.7E-07 | 0.70 | 2.2E-01 | Kinesin-like protein |
| Solyc04g005040.1.1 | SlMMP2 | -2.25 | 3.3E-07 | -2.01 | 4.5E-06 | -0.24 | 7.4E-01 | Metalloendoproteinase.1 |
| Solyc04g071650.4.1 | LOC101248382 | -2.25 | 3.8E-07 | -1.63 | 1.4E-04 | -0.61 | 3.8E-01 | Cellulose synthase |
| Solyc01g097000.3.1 | LOC101254239 | -2.25 | 3.4E-04 | -2.40 | 1.1E-04 | 0.15 | 9.3E-01 | Glycosyl hydrolase family protein |
| Solyc09g098540.3.1 | LOC101254426 | -2.26 | 2.3E-06 | -2.08 | 1.4E-05 | -0.18 | 8.4E-01 | Chitinase-like protein |
| Solyc02g069250.5.1 | LOC101249426 | -2.26 | 2.0E-05 | -2.61 | 7.4E-07 | 0.36 | 7.3E-01 | Alcohol dehydrogenase superfamily |
| Solyc03g122190.3.1 | SRG1 | -2.26 | 6.3E-08 | -1.80 | 2.7E-05 | -0.46 | 4.0E-01 | Protein TIFY 10A |
| Solyc01g081540.4.1 | LOC101264152 | -2.27 | 3.9E-08 | -1.88 | 4.4E-06 | -0.38 | 5.2E-01 | Myosin-17 |
| Solyc08g080040.4.1 | LOC101251607 | -2.27 | 7.0E-05 | -2.46 | 1.8E-05 | 0.19 | 9.1E-01 | 2-oxoglutarate (2OG) and Fe(II)-dependent oxygenase superfamily protein |
| Solyc06g076220.3.1 | exp18 | -2.27 | 8.0E-05 | -1.84 | 1.2E-03 | -0.43 | 5.5E-01 | expansin18 |
| Solyc12g049400.2.1 | LOC101263193 | -2.28 | 8.3E-06 | -2.56 | 2.8E-07 | 0.28 | 7.1E-01 | Tify |
| Solyc01g109700.3.1 | LOC101252729 | -2.28 | 4.2E-06 | -2.45 | 5.7E-07 | 0.17 | 9.0E-01 | bHLH transcription factor 010 |
| Solyc07g018010.3.1 | LOC101266736 | -2.30 | 2.7E-07 | -2.40 | 4.5E-08 | 0.10 | 9.2E-01 | bHLH transcription factor139 |
| Solyc07g065880.4.1 | LOC101261343 | -2.31 | 1.3E-05 | -2.31 | 9.5E-06 | 0.01 | 9.5E-01 | Kinesin K.1 |
| Solyc02g085020.4.1 | LOC544150 | -2.31 | 3.6E-06 | -2.33 | 5.3E-06 | 0.02 | 9.4E-01 | dihydroflavonol 4-reductase |
| Solyc04g008040.4.1 | LOC101264814 | -2.32 | 1.1E-04 | -2.20 | 1.9E-04 | -0.12 | 9.1E-01 | Microtubule-associated protein 70 |
| Solyc09g007640.4.1 | LOC101250043 | -2.33 | 1.6E-05 | -2.05 | 9.0E-05 | -0.28 | 6.5E-01 | Serine carboxypeptidase-like 50 |
| Solyc01g111830.3.1 | LOC101247376 | -2.33 | 1.1E-06 | -2.56 | 8.2E-08 | 0.23 | 8.5E-01 | Methyltransferase |
| Solyc02g084390.3.1 | LOC101252039 | -2.33 | 1.7E-06 | -2.03 | 2.7E-05 | -0.30 | 6.6E-01 | Kinesin-like protein |
| Solyc06g075580.4.1 | LOC101257270 | -2.33 | 1.5E-05 | -1.99 | 1.7E-04 | -0.35 | 6.9E-01 | Kinesin |
| Solyc07g042390.3.1 | LOC101244503 | -2.33 | 3.3E-04 | -2.43 | 2.1E-04 | 0.10 | 9.9E-01 | Plant invertase/pectin methylesterase inhibitor superfamily protein |
| Solyc01g110130.3.1 | LOC101264867 | -2.34 | 1.0E-05 | -2.83 | 8.1E-08 | 0.49 | 5.8E-01 | DNA helicase |
| Solyc04g078740.2.1 | LOC101260044 | -2.34 | 2.4E-06 | -2.59 | 8.8E-08 | 0.25 | 7.4E-01 | Subtilisin-like protease-like protein |
| Solyc12g009930.3.1 | LOC101265706 | -2.34 | 1.7E-06 | -1.92 | 5.4E-05 | -0.42 | 6.2E-01 | Glycosyltransferase |
| Solyc01g079110.5.1 | LOC101260571 | -2.34 | 7.6E-04 | -3.29 | 2.0E-06 | 0.95 | 2.6E-01 | Histone H3 |
| Solyc09g084460.3.1 | LOC101247257 | -2.35 | 7.0E-04 | -2.89 | 9.6E-05 | 0.54 | 8.2E-01 | Protease inhibitor I |
| Solyc11g021060.2.1 | ARPI | -2.35 | 1.6E-03 | -0.87 | 9.1E-02 | -1.48 | 3.0E-01 | TOMARPIX proteinase inhibitor |
| Solyc02g085730.3.1 | AOC | -2.35 | 3.9E-09 | -2.25 | 1.2E-08 | -0.10 | 8.8E-01 | allene oxide cyclase |
| Solyc03g006830.3.1 | FYFL | -2.36 | 6.4E-06 | -2.49 | 1.3E-06 | 0.14 | 9.6E-01 | MADS-box transcription factor |
| Solyc06g066650.4.1 | LOC101248400 | -2.36 | 6.7E-06 | -2.32 | 6.1E-06 | -0.04 | 9.5E-01 | Rop guanine nucleotide exchange factor 12 |
| Solyc03g006970.1.1 | SBT2 | -2.37 | 3.9E-04 | -2.02 | 2.1E-03 | -0.34 | 7.6E-01 | serine protease SBT2 |
| Solyc02g085120.3.1 | LOC101262977 | -2.37 | 2.7E-04 | -2.89 | 1.0E-05 | 0.52 | 6.7E-01 | Laccase |
| Solyc05g053890.3.1 | LOC101244489 | -2.37 | 3.5E-05 | -1.98 | 4.2E-04 | -0.39 | 5.9E-01 | Glycosyltransferase |
| Solyc11g010810.1.1 | LOC101250468 | -2.37 | 3.1E-06 | -1.46 | 3.2E-03 | -0.91 | 1.0E-01 | UDP-glycosyltransferase 91.1 |
| Solyc03g083770.1.1 | LOC101248367 | -2.37 | 4.9E-04 | -1.71 | 1.2E-02 | -0.66 | 4.7E-01 | Plant invertase/pectin methylesterase inhibitor superfamily protein, putative |
| Solyc01g097290.4.1 | SlIAA16 | -2.37 | 5.0E-06 | -2.06 | 5.9E-05 | -0.31 | 7.1E-01 | auxin-regulated IAA16 |
| Solyc02g069490.4.1 | LOC101244831 | -2.38 | 9.0E-06 | -1.88 | 2.8E-04 | -0.51 | 6.2E-01 | Delta(24)-sterol reductase |
| Solyc11g008810.2.1 | LOC101253853 | -2.38 | 8.5E-05 | -2.65 | 1.7E-05 | 0.27 | 9.1E-01 | Beta-hexosaminidase |
| Solyc08g068610.3.1 | AADC1B | -2.38 | 1.5E-05 | -3.23 | 4.3E-09 | 0.85 | 2.9E-01 | Serine decarboxylase |
| Solyc09g092330.3.1 | LOC101247369 | -2.38 | 1.0E-07 | -1.78 | 3.7E-05 | -0.61 | 3.7E-01 | UDP-glucuronate 4-epimerase 4 |
| Solyc07g064030.4.1 | LOC101260250 | -2.39 | 7.0E-10 | -2.65 | 1.7E-12 | 0.26 | 6.5E-01 | Kinesin-like protein |
| Solyc06g068770.3.1 | LOC101250897 | -2.41 | 2.1E-05 | -2.33 | 3.2E-05 | -0.08 | 8.7E-01 | DB279 |
| Solyc09g075550.3.1 | LOC101262856 | -2.41 | 3.9E-05 | -2.56 | 9.4E-06 | 0.15 | 9.3E-01 | Cellulose synthase |
| Solyc06g009780.4.1 | LOC101268475 | -2.41 | 1.7E-04 | -2.58 | 6.1E-05 | 0.17 | 9.5E-01 | Kinesin-4 |
| Solyc06g074850.3.1 | LOC101267900 | -2.42 | 1.4E-04 | -3.10 | 1.0E-06 | 0.68 | 4.8E-01 | Carboxypeptidase |
| Solyc06g005750.3.1 | LOC101258266 | -2.42 | 8.0E-07 | -1.35 | 1.2E-03 | -1.07 | 2.0E-01 | Methylsterol monooxygenase 2.1 |
| Solyc07g062130.3.1 | LOC101249663 | -2.42 | 1.9E-09 | -1.79 | 8.4E-06 | -0.64 | 2.5E-01 | trifunctional UDP-glucose 4,6-dehydratase/UDP-4-keto-6-deoxy-D-glucose 3,5-epimerase/UDP-4-keto-L-rhamnose-reductase RH.1 |
| Solyc01g008110.5.1 | CYP51 | -2.43 | 7.0E-10 | -2.00 | 4.0E-07 | -0.43 | 4.6E-01 | Cytochrome P450 |
| Solyc01g110190.4.1 | LOC101264563 | -2.44 | 9.3E-05 | -1.87 | 1.8E-03 | -0.56 | 5.1E-01 | Kinesin-related protein |
| Solyc08g068600.3.1 | LOC101264847 | -2.44 | 9.4E-08 | -2.33 | 3.1E-07 | -0.11 | 9.0E-01 | Serine decarboxylase |
| Solyc07g039310.1.1 | LOC101244015 | -2.44 | 6.0E-06 | -2.58 | 1.1E-06 | 0.14 | 9.3E-01 | lysine-specific histone demethylase.1 |
| Solyc03g031800.3.1 | LOC101256456 | -2.44 | 1.0E-05 | -2.39 | 8.6E-06 | -0.05 | 9.7E-01 | Xyloglucan endotransglucosylase/hydrolase |
| Solyc06g083580.4.1 | LOC101244298 | -2.45 | 1.6E-05 | -2.23 | 5.6E-05 | -0.22 | 8.6E-01 | Pectate lyase |
| Solyc06g074670.4.1 | LOC101245851 | -2.46 | 6.7E-13 | -2.32 | 6.3E-12 | -0.14 | 8.2E-01 | UDP-apiose/UDP-xylose synthase |
| Solyc12g098590.3.1 | LOC101257156 | -2.47 | 3.0E-05 | -2.19 | 1.9E-04 | -0.28 | 7.8E-01 | Glycosyltransferase |
| Solyc09g083290.3.1 | LOC101055547 | -2.47 | 3.8E-06 | -2.19 | 3.2E-05 | -0.28 | 7.8E-01 | auxin-regulated IAA14 |
| Solyc05g052240.3.1 | LOC101266223 | -2.48 | 3.0E-06 | -1.29 | 8.3E-03 | -1.18 | 1.0E-01 | Chalcone-flavonone isomerase family protein |
| Solyc12g055840.2.1 | LOC101249056 | -2.48 | 4.2E-05 | -2.59 | 1.8E-05 | 0.11 | 9.7E-01 | Glucan endo-1,3-beta-glucosidase 10 |
| Solyc06g009190.5.1 | LOC101260941 | -2.48 | 6.4E-04 | -2.00 | 4.3E-03 | -0.49 | 7.2E-01 | Pectinesterase |
| Solyc07g005020.3.1 | LOC101256302 | -2.49 | 1.3E-05 | -3.05 | 5.1E-08 | 0.56 | 4.3E-01 | DNA helicase |
| Solyc11g010920.3.1 | LOC101265093 | -2.50 | 3.8E-05 | -2.63 | 1.1E-05 | 0.13 | 9.4E-01 | Kinesin-4 |
| Solyc07g043490.1.1 | GAME1 | -2.52 | 1.5E-08 | -2.48 | 1.9E-08 | -0.04 | 9.6E-01 | Glycosyltransferase |
| Solyc04g076310.4.1 | LOC101246423 | -2.53 | 6.7E-05 | -3.03 | 1.4E-06 | 0.50 | 5.6E-01 | Kinesin-like protein FR.1 |
| Solyc02g077030.3.1 | LOC101249701 | -2.55 | 2.0E-05 | -3.10 | 1.6E-07 | 0.55 | 5.2E-01 | Phospholipase A1-II.1 |
| Solyc11g072820.3.1 | LOC101255463 | -2.56 | 1.3E-07 | -2.20 | 3.5E-06 | -0.35 | 5.9E-01 | Kinesin |
| Solyc02g084720.3.1 | TBG6 | -2.56 | 4.4E-10 | -1.96 | 1.4E-06 | -0.60 | 2.8E-01 | beta-galactosidase 6 |
| Solyc03g113450.4.1 | LOC101266408 | -2.57 | 2.5E-04 | -2.86 | 3.2E-05 | 0.29 | 7.9E-01 | Receptor-like protein kinase |
| Solyc09g092520.3.1 | LOC543637 | -2.57 | 1.3E-05 | -2.36 | 4.4E-05 | -0.21 | 8.7E-01 | xyloglucan endotransglycosylase |
| Solyc05g014000.5.1 | LOC101252082 | -2.57 | 3.6E-06 | -2.56 | 5.4E-06 | -0.01 | 9.3E-01 | Pectate lyase |
| Solyc04g012060.3.1 | LOC101249065 | -2.57 | 8.2E-07 | -2.88 | 1.9E-08 | 0.31 | 7.4E-01 | Ribonucleoside-diphosphate reductase |
| Solyc01g099410.3.1 | HTA6 | -2.57 | 1.6E-05 | -3.25 | 1.9E-08 | 0.68 | 2.9E-01 | Histone H2A |
| Solyc06g008590.3.1 | IAA10 | -2.58 | 6.4E-05 | -2.26 | 3.3E-04 | -0.32 | 7.7E-01 | auxin-regulated IAA17 |
| Solyc07g009380.5.1 | XET2 | -2.58 | 1.8E-06 | -1.95 | 4.8E-05 | -0.63 | 5.7E-01 | Xyloglucan endotransglucosylase/hydrolase |
| Solyc07g005960.3.1 | LOC101251103 | -2.58 | 9.3E-09 | -2.84 | 8.6E-11 | 0.25 | 6.6E-01 | Carboxypeptidase |
| Solyc06g082570.3.1 | LOC101244780 | -2.61 | 6.7E-05 | -1.90 | 2.9E-03 | -0.71 | 3.9E-01 | protein NETWORKED 4B |
| Solyc09g091510.3.1 | CHS1 | -2.62 | 1.4E-04 | -2.00 | 3.1E-03 | -0.62 | 5.9E-01 | chalcone synthase 1 |
| Solyc07g042520.4.1 | LOC101267720 | -2.65 | 2.0E-06 | -3.39 | 6.8E-08 | 0.73 | 7.7E-01 | Sucrose synthase |
| Solyc03g097030.3.1 | LOC101251363 | -2.65 | 1.6E-08 | -1.56 | 7.5E-04 | -1.10 | 4.3E-02 | 4-coumarate:CoA ligase |
| Solyc11g018772.1.1 | LOC112940337 | -2.66 | 2.1E-04 | -3.69 | 1.6E-07 | 1.03 | 4.3E-02 | Peroxidase |
| Solyc09g083380.5.1 | LOC101245495 | -2.67 | 4.5E-07 | -3.05 | 4.8E-09 | 0.38 | 6.6E-01 | Histone H1 |
| Solyc07g017600.3.1 | LOC101261426 | -2.67 | 1.1E-07 | -2.56 | 2.1E-07 | -0.11 | 9.2E-01 | Pectinesterase |
| Solyc03g083900.4.1 | LOC101247502 | -2.68 | 3.1E-09 | -3.04 | 3.5E-12 | 0.36 | 5.2E-01 | monocopper oxidase-like protein SKU5 |
| Solyc03g119080.4.1 | LOC543698 | -2.70 | 1.1E-06 | -3.02 | 2.4E-08 | 0.33 | 7.1E-01 | beta-mannosidase enzyme |
| Solyc03g123620.4.1 | LOC101245743 | -2.70 | 3.2E-10 | -2.55 | 1.3E-09 | -0.15 | 8.7E-01 | Pectinesterase |
| Solyc10g085870.1.1 | LOC101261675 | -2.71 | 1.2E-05 | -0.27 | 6.6E-01 | -2.44 | 7.3E-05 | Glycosyltransferase |
| Solyc01g105360.3.1 | LOC101254539 | -2.71 | 1.1E-05 | -2.22 | 3.6E-04 | -0.49 | 4.2E-01 | Glycosyltransferase |
| Solyc12g088170.2.1 | LOC100191129 | -2.71 | 1.7E-06 | -2.59 | 6.9E-06 | -0.12 | 8.5E-01 | HXXXD-type acyl-transferase family protein |
| Solyc01g079500.5.1 | LOC101243636 | -2.72 | 1.1E-06 | -3.27 | 2.1E-09 | 0.55 | 4.1E-01 | DNA replication licensing factor MCM7 |
| Solyc03g007130.4.1 | LOC101246993 | -2.72 | 3.2E-05 | -3.03 | 2.8E-06 | 0.31 | 8.1E-01 | 65-kDa microtubule-associated protein 3,Pfam:PF03999 |
| Solyc08g080170.3.1 | LOC101249394 | -2.72 | 7.2E-08 | -2.43 | 1.2E-06 | -0.29 | 7.5E-01 | 3-hydroxy-3-methylglutaryl coenzyme A synthase |
| Solyc07g065210.5.1 | TKR | -2.76 | 6.0E-05 | -3.09 | 5.0E-06 | 0.34 | 7.7E-01 | Kinesin-like protein KIN12B-like |
| Solyc08g079090.4.1 | LOC101247352 | -2.79 | 8.3E-08 | -2.64 | 3.8E-07 | -0.16 | 8.7E-01 | Monocopper oxidase-like protein sku5 |
| Solyc01g079570.3.1 | SlArf/Xyl2 | -2.80 | 5.5E-05 | -3.37 | 3.1E-06 | 0.57 | 7.8E-01 | Beta-D-xylosidase.1 |
| Solyc02g089620.3.1 | PDH | -2.80 | 5.8E-13 | -2.63 | 5.4E-12 | -0.17 | 8.3E-01 | proline dehydrogenase |
| Solyc06g051960.3.1 | LOC101258375 | -2.80 | 4.7E-08 | -2.18 | 1.2E-05 | -0.62 | 4.3E-01 | Pectinesterase |
| Solyc09g059170.3.1 | LOC101244316 | -2.82 | 1.4E-06 | -1.73 | 1.9E-03 | -1.09 | 1.4E-01 | Glycosyltransferase |
| Solyc07g052240.3.1 | LOC101256015 | -2.85 | 8.8E-06 | -2.95 | 3.0E-06 | 0.10 | 9.7E-01 | L-ascorbate oxidase-like protein |
| Solyc03g113960.4.1 | LOC101255381 | -2.86 | 9.6E-06 | -3.19 | 7.3E-07 | 0.33 | 7.5E-01 | Calmodulin binding protein-like |
| Solyc01g100240.3.1 | LOC101267354 | -2.86 | 1.3E-12 | -2.85 | 5.1E-13 | -0.01 | 9.5E-01 | Calmodulin binding protein-like |
| Solyc10g086240.2.1 | LOC101261977 | -2.86 | 2.1E-10 | -3.13 | 1.6E-12 | 0.27 | 7.8E-01 | Glycosyltransferase |
| Solyc01g107800.4.1 | LOC101257020 | -2.87 | 9.0E-13 | -2.57 | 4.9E-11 | -0.30 | 5.7E-01 | glucuronoxylan 4-O-methyltransferase 1-like |
| Solyc10g008910.1.1 | LOC101250929 | -2.89 | 8.0E-07 | -3.52 | 2.2E-09 | 0.64 | 5.5E-01 | Histone H3 |
| Solyc05g010320.4.1 | CHI1 | -2.91 | 2.2E-05 | -2.71 | 5.5E-05 | -0.20 | 9.0E-01 | Chalcone-flavonone isomerase family protein |
| Solyc06g074790.3.1 | H2B-1 | -2.91 | 8.3E-06 | -3.73 | 7.3E-09 | 0.82 | 2.7E-01 | Histone H2B |
| Solyc09g061890.3.1 | LOC101263458 | -2.93 | 6.9E-06 | -2.78 | 1.2E-05 | -0.15 | 9.1E-01 | Pectate lyase |
| Solyc09g008320.5.1 |  | -2.94 | 6.5E-06 | -2.91 | 4.9E-06 | -0.02 | 9.9E-01 | Xyloglucan endotransglucosylase/hydrolase |
| Solyc09g084450.3.1 | LOC101247557 | -2.94 | 3.7E-04 | -1.81 | 1.6E-02 | -1.14 | 3.8E-01 | Protease inhibitor I |
| Solyc01g079180.4.1 | LOC101261743 | -2.96 | 6.1E-05 | -3.06 | 3.8E-05 | 0.11 | 9.9E-01 | Pectinesterase |
| Solyc09g075480.3.1 | LOC101264370 | -2.99 | 4.5E-07 | -3.19 | 4.5E-08 | 0.20 | 8.9E-01 | Kinesin-4 |
| Solyc01g005000.3.1 | GAD3 | -3.00 | 3.0E-06 | -3.84 | 1.6E-09 | 0.84 | 2.7E-01 | Glutamate decarboxylase |
| Solyc02g083860.3.1 | F3H | -3.01 | 2.1E-09 | -2.46 | 7.2E-07 | -0.54 | 4.8E-01 | 2-oxoglutarate (2OG) and Fe(II)-dependent oxygenase superfamily protein |
| Solyc10g083440.3.1 | LOC101260093 | -3.01 | 3.8E-08 | -2.22 | 3.5E-05 | -0.79 | 3.1E-01 | Glycosyltransferase |
| Solyc07g064870.3.1 | LOC101267141 | -3.01 | 4.1E-05 | -2.82 | 8.1E-05 | -0.19 | 8.8E-01 | Endoglucanase |
| Solyc03g071570.3.1 | LOC101260911 | -3.02 | 5.2E-08 | -2.96 | 6.9E-08 | -0.07 | 9.1E-01 | Pectate lyase |
| Solyc05g009820.5.1 | LOC101265331 | -3.03 | 9.5E-08 | -2.61 | 2.7E-06 | -0.41 | 5.8E-01 | Hexosyltransferase |
| Solyc01g108670.4.1 | LOC101264472 | -3.04 | 2.4E-06 | -2.89 | 5.7E-06 | -0.15 | 8.6E-01 | Kinesin |
| Solyc03g115380.3.1 | LOC101261402 | -3.12 | 4.0E-05 | -2.93 | 3.9E-05 | -0.18 | 9.9E-01 | UDP-glucose 6-dehydrogenase |
| Solyc04g079860.1.1 | LOC101247911 | -3.14 | 7.8E-11 | -2.95 | 3.4E-10 | -0.19 | 8.0E-01 | Hexosyltransferase |
| Solyc07g005840.2.1 | LOC101248888 | -3.14 | 5.7E-09 | -3.18 | 2.3E-09 | 0.04 | 9.7E-01 | Cellulose synthase |
| Solyc04g082140.3.1 | LOC778302 | -3.17 | 4.4E-10 | -3.15 | 6.6E-10 | -0.01 | 9.5E-01 | pectinesterase |
| Solyc09g083360.3.1 | LOC101245202 | -3.17 | 5.0E-08 | -2.56 | 7.8E-06 | -0.60 | 4.8E-01 | Transcription factor bHLH92 |
| Solyc02g092670.1.1 | LOC101255349 | -3.18 | 2.3E-15 | -2.97 | 3.1E-14 | -0.21 | 7.5E-01 | Subtilisin-like protease |
| Solyc11g011030.2.1 | Prg1 | -3.19 | 5.1E-08 | -2.19 | 1.3E-04 | -1.01 | 1.5E-01 | Pto-responsive gene 1 |
| Solyc08g068390.3.1 | LOC101261255 | -3.21 | 1.4E-07 | -2.51 | 3.3E-05 | -0.70 | 4.2E-01 | Glyoxysomal fatty acid beta-oxidation multifunctional protein MFP-a |
| Solyc02g088100.3.1 | EXPA5 | -3.25 | 3.1E-09 | -2.93 | 6.8E-08 | -0.32 | 7.2E-01 | expansin precursor 5 |
| Solyc09g072820.5.1 | LOC101246281 | -3.26 | 1.2E-09 | -3.28 | 8.0E-10 | 0.03 | 9.3E-01 | Cellulose synthase |
| Solyc01g091170.3.1 | ARG2 | -3.27 | 2.0E-05 | -2.80 | 3.7E-04 | -0.48 | 5.8E-01 | ARGINASE 2 |
| Solyc07g049300.3.1 | LOC101244887 | -3.28 | 5.6E-09 | -2.78 | 3.6E-07 | -0.51 | 5.4E-01 | Endoglucanase |
| Solyc04g079730.1.1 | aos | -3.31 | 2.2E-10 | -3.56 | 1.7E-12 | 0.24 | 7.0E-01 | allene oxide synthase |
| Solyc12g057080.2.1 | LOC101248084 | -3.33 | 1.1E-05 | -3.14 | 2.7E-05 | -0.19 | 9.2E-01 | Glycosyltransferase |
| Solyc02g064830.4.1 | LOC101268544 | -3.33 | 2.1E-05 | -3.58 | 1.8E-06 | 0.25 | 7.1E-01 | Indole-3-acetic acid-amido synthetas 3-3 |
| Solyc04g078110.1.1 | SBT1 | -3.38 | 8.3E-09 | -3.07 | 6.1E-08 | -0.31 | 6.9E-01 | serine protease SBT1 |
| Solyc11g008630.3.1 | LOC101249696 | -3.38 | 2.7E-09 | -2.10 | 1.1E-04 | -1.29 | 5.6E-02 | HXXXD-type acyl-transferase family protein |
| Solyc02g072240.3.1 | LOC101267672 | -3.39 | 5.5E-09 | -3.67 | 1.4E-10 | 0.27 | 8.5E-01 | Cellulose synthase |
| Solyc11g022590.1.1 | LOC544001 | -3.40 | 1.9E-05 | -1.91 | 4.8E-03 | -1.48 | 2.7E-01 | trypsin inhibitor-like protein precursor |
| Solyc03g098300.1.1 | LOC101255673 | -3.41 | 1.3E-05 | -3.15 | 3.3E-05 | -0.26 | 8.7E-01 | Ornithine decarboxylase |
| Solyc02g063000.5.1 | LOC101261193 | -3.44 | 3.4E-10 | -1.71 | 9.3E-04 | -1.73 | 3.3E-03 | Glycosyltransferase |
| Solyc07g049370.2.1 | LOC101268410 | -3.46 | 1.4E-06 | -3.51 | 6.2E-07 | 0.05 | 9.6E-01 | Glucan endo-1,3-beta-glucosidase 3 |
| Solyc05g047590.5.1 | LOC101261415 | -3.47 | 3.9E-08 | -3.00 | 1.0E-06 | -0.47 | 5.3E-01 | Pectinesterase |
| Solyc11g062440.2.1 | LOC101258916 | -3.49 | 3.1E-13 | -3.79 | 9.1E-16 | 0.30 | 7.9E-01 | L-ascorbate oxidase |
| Solyc08g074683.1.1 | PPO | -3.50 | 3.8E-06 | -4.27 | 3.5E-08 | 0.77 | 5.5E-01 | Polyphenol oxidase |
| Solyc08g074620.3.1 | LOC101259357 | -3.52 | 3.4E-08 | -3.52 | 1.3E-08 | 0.00 | 9.7E-01 | Polyphenol oxidase |
| Solyc12g011030.3.1 | LOC101261784 | -3.57 | 2.5E-09 | -3.47 | 2.8E-09 | -0.09 | 9.5E-01 | xyloglucan endotransglucosylase-hydrolase 9 |
| Solyc12g010980.3.1 | LOC101260610 | -3.60 | 1.7E-07 | -3.56 | 1.3E-07 | -0.04 | 9.7E-01 | HXXXD-type acyl-transferase family protein |
| Solyc03g083360.3.1 | LOC101255775 | -3.63 | 8.2E-15 | -2.57 | 1.1E-08 | -1.06 | 5.6E-02 | Pectinesterase |
| Solyc01g111100.6.1 | LOC101253328 | -3.70 | 7.2E-07 | -3.96 | 8.5E-08 | 0.25 | 8.2E-01 | Neutral/alkaline invertase |
| Solyc08g074630.2.1 | LOC101259064 | -3.70 | 4.0E-07 | -3.93 | 4.8E-08 | 0.23 | 8.7E-01 | Polyphenol oxidase |
| Solyc12g011023.1.1 | LOC101261784 | -3.72 | 9.7E-07 | -4.00 | 1.7E-07 | 0.27 | 8.6E-01 | Xyloglucan endotransglucosylase/hydrolase |
| Solyc01g087590.3.1 | LOC101253022 | -3.76 | 6.4E-12 | -3.11 | 5.8E-09 | -0.64 | 4.5E-01 | Polyamine oxidase |
| Solyc01g091050.5.1 | LOC101247960 | -3.81 | 1.1E-10 | -2.92 | 3.2E-07 | -0.89 | 1.6E-01 | Pectinesterase |
| Solyc05g053550.3.1 | CHS2 | -3.82 | 1.7E-12 | -2.41 | 3.3E-06 | -1.41 | 5.6E-02 | chalcone synthase 2 |
| Solyc09g014240.4.1 | LOC101253311 | -3.87 | 5.9E-10 | -4.24 | 6.1E-12 | 0.36 | 8.2E-01 | Laccase |
| Solyc03g031730.3.1 | LOC101255272 | -4.00 | 8.3E-10 | -3.91 | 3.3E-10 | -0.09 | 9.6E-01 | Beta-glucosidase |
| Solyc10g079320.3.1 | LOC101253738 | -4.03 | 6.4E-07 | -3.13 | 3.9E-05 | -0.90 | 3.1E-01 | Glycosyltransferase |
| Solyc02g089630.3.1 | LOC101268445 | -4.05 | 1.5E-08 | -2.75 | 3.7E-05 | -1.30 | 7.9E-02 | Proline dehydrogenase |
| Solyc07g052980.3.1 | XTH16 | -4.23 | 6.2E-11 | -4.04 | 1.6E-10 | -0.20 | 9.0E-01 | xyloglucan endotransglycosylase/hydrolase 16 |
| Solyc11g020960.2.1 | LOC101255652 | -4.26 | 4.9E-08 | -4.69 | 8.5E-10 | 0.43 | 5.5E-01 | Proteinase inhibitor type-2 |
| Solyc04g078460.3.1 | LOC101245157 | -4.87 | 2.0E-18 | -4.81 | 6.1E-19 | -0.06 | 9.7E-01 | Isoaspartyl peptidase/L-asparaginase |

Supplementary Table 2 Enriched Gene Ontology (GO) terms and genes (*C*Lso haplotype B vs. negative).

| Cate-gory | GO ID | GO Term | P Value | Adjusted P Value | Core Gene |
| --- | --- | --- | --- | --- | --- |
| CC | GO:0005576 | extracellular region | 1.2E-03 | 4.6E-02 | tXET-B1/EXPA4/Sl3-MMP/AO/XTH3/LOC101258926/LOC101258345/tXET-B2/XTH7/LOC543795/LOC101258632/SlMMP2/exp18/LOC101262977/LOC101256456/LOC543637/XET2/Solyc09g008320.5.1/EXPA5/LOC101258916/LOC101261784/LOC101261784/LOC101253311/XTH16 |
| CC | GO:0048046 | apoplast | 1.3E-03 | 4.6E-02 | XTH3/LOC101258926/LOC101258345/tXET-B2/XTH7/LOC101258632/LOC101262977/LOC101256456/LOC543637/XET2/Solyc09g008320.5.1/LOC101261784/LOC101261784/LOC101253311/XTH16 |
| CC | GO:0005618 | cell wall | 1.3E-03 | 4.6E-02 | XTH3/LOC101258926/LOC101258345/tXET-B2/XTH7/LOC101258632/LOC101256456/LOC543637/XET2/Solyc09g008320.5.1/LOC101261784/LOC101261784/XTH16 |
| CC | GO:0030312 | external encapsulating structure | 1.3E-03 | 4.6E-02 | XTH3/LOC101258926/LOC101258345/tXET-B2/XTH7/LOC101258632/LOC101256456/LOC543637/XET2/Solyc09g008320.5.1/LOC101261784/LOC101261784/XTH16 |
| CC | GO:0044436 | thylakoid part | 2.2E-03 | 4.6E-02 | Solyc00g500054.1.1/LOC101259747/psaJ/Solyc09g064580.2.1/LOC101259494/LOC101243864/psbB/psaB/Solyc07g032620.3.1/LOC101266666/LOC101245121/LOC101248983/LOC101263732/LOC101256427/LOC543978/LOC101255222/LOC543930/Solyc00g500049.1.1/LOC101265555/LOC101264609/LOC101245880/psbZ/PSBP/PSBR/LOC101259227/PsbQ/LOC101266426/LOC101244751/LOC101268297/LOC101256723/LOC101251312/PSBO/LOC101265249/LOC101254767/LOC101245421/LOC101257676 |
| CC | GO:0000786 | nucleosome | 2.4E-03 | 4.6E-02 | Solyc06g074780.1.1/LOC544102/H1/LOC101267024/H2B-3/LOC101256806/Solyc03g071620.2.1/LOC101268754/LOC101265996/LOC101253792/LOC101265669/LOC101252717/Solyc01g086820.5.1/LOC101267861/LOC101248710/H2B-2/LOC101260571/HTA6/LOC101245495/LOC101250929/H2B-1 |
| CC | GO:0032993 | protein-DNA complex | 2.4E-03 | 4.6E-02 | Solyc06g074780.1.1/LOC544102/H1/LOC101267024/H2B-3/LOC101256806/Solyc03g071620.2.1/LOC101268754/LOC101265996/LOC101253792/LOC101265669/LOC101252717/Solyc01g086820.5.1/LOC101267861/LOC101248710/H2B-2/LOC101260571/HTA6/LOC101245495/LOC101250929/H2B-1 |
| CC | GO:0044815 | DNA packaging complex | 2.4E-03 | 4.6E-02 | Solyc06g074780.1.1/LOC544102/H1/LOC101267024/H2B-3/LOC101256806/Solyc03g071620.2.1/LOC101268754/LOC101265996/LOC101253792/LOC101265669/LOC101252717/Solyc01g086820.5.1/LOC101267861/LOC101248710/H2B-2/LOC101260571/HTA6/LOC101245495/LOC101250929/H2B-1 |
| CC | GO:0009579 | thylakoid | 3.2E-03 | 4.6E-02 | Solyc00g500054.1.1/LOC101259747/psaJ/Solyc09g064580.2.1/LOC101259494/psaA/LOC101243864/psbB/psaB/Solyc07g032620.3.1/LOC101266666/LOC101249276/LOC101245121/LOC101248983/LOC101262260/LOC101263732/LOC101256427/LOC543978/LOC101255222/LOC543930/Solyc00g500049.1.1/LOC101265555/LOC101264609/LOC101245880/psbZ/PSBP/PSBR/LOC101259227/PsbQ/LOC101266426/LOC101244751/LOC101260274/LOC101268297/LOC101256723/LOC101251312/PSBO/LOC101265249/LOC101254767/LOC101245421/LOC101257676 |
| CC | GO:0071944 | cell periphery | 3.2E-03 | 4.6E-02 | LOC101259136/LOC101261300/LOC101248224/LOC101256188/LOC101254331/LOC101255416/LOC101253679/tXET-B1/LOC101250560/LOC101252007/XTH3/LOC101263837/LOC101255283/LOC101258926/LOC101258345/tXET-B2/XTH7/LOC101258632/LOC101256456/LOC543637/XET2/Solyc09g008320.5.1/LOC101261784/LOC101261784/XTH16 |
| CC | GO:0034357 | photosynthetic membrane | 3.2E-03 | 4.6E-02 | Solyc00g500054.1.1/LOC101259747/psaJ/Solyc09g064580.2.1/LOC101259494/LOC101243864/psbB/psaB/Solyc07g032620.3.1/LOC101266666/LOC101245121/LOC101248983/LOC101263732/LOC543978/LOC101255222/LOC543930/Solyc00g500049.1.1/LOC101265555/LOC101264609/LOC101245880/psbZ/PSBP/PSBR/LOC101259227/PsbQ/LOC101266426/LOC101244751/LOC101268297/LOC101256723/LOC101251312/PSBO/LOC101265249/LOC101254767/LOC101245421/LOC101257676 |
| CC | GO:0000785 | chromatin | 3.4E-03 | 4.6E-02 | Solyc06g074780.1.1/LOC544102/H1/LOC101267024/H2B-3/LOC101256806/Solyc03g071620.2.1/LOC101268754/LOC101265996/LOC101253792/LOC101265669/LOC101252717/Solyc01g086820.5.1/LOC101267861/LOC101248710/H2B-2/LOC101260571/HTA6/LOC101245495/LOC101250929/H2B-1 |
| MF | GO:0016757 | transferase activity, transferring glycosyl groups | 1.0E-03 | 2.1E-02 | LOC101247727/LOC101261450/LOC101252560/LOC101262652/LOC101263892/LOC101257246/LOC101245582/LOC101254331/LOC101265913/Solyc04g079030.2.1/LOC101248226/LOC101246582/LOC101252577/LOC101268420/LOC101255416/LOC101259456/LOC101247341/LOC101252941/LOC101250314/LOC101244432/LOC101268547/LOC101245582/LOC101264614/LOC101255991/tXET-B1/LOC101262558/LOC101247901/LOC101261539/LOC101252007/LOC101265190/LOC101257919/LOC101258278/LOC100134910/LOC101257137/LOC101248295/LOC101254610/XTH3/LOC101255377/LOC101263837/LOC101245676/LOC101260024/LOC101251579/LOC101246362/GBSS1/LOC101250703/LOC101266782/LOC101263331/LOC101258926/LOC101256480/GGP/LOC101251138/LOC101263304/LOC101258941/LOC101260885/LOC101263151/LOC101248301/LOC101259856/LOC101268254/LOC101246863/LOC101256305/LOC101258345/LOC101247596/LOC101254109/tXET-B2/LOC101248301/XTH7/LOC101258632/LOC101254402/LOC101259712/LOC101248382/LOC101265706/LOC101244489/LOC101250468/LOC101250897/LOC101262856/LOC101256456/LOC101257156/GAME1/LOC543637/XET2/LOC101267720/LOC101261675/LOC101254539/LOC101244316/LOC101261977/Solyc09g008320.5.1/LOC101260093/LOC101265331/LOC101247911/LOC101248888/LOC101246281/LOC101248084/LOC101267672/LOC101261193/LOC101261784/LOC101261784/LOC101253738/XTH16 |
| MF | GO:0016758 | transferase activity, transferring hexosyl groups | 1.0E-03 | 2.1E-02 | LOC101247727/LOC101261450/LOC101263892/LOC101257246/LOC101254331/LOC101265913/Solyc04g079030.2.1/LOC101246582/LOC101252577/LOC101268420/LOC101255416/LOC101259456/LOC101252941/LOC101250314/LOC101244432/LOC101268547/LOC101264614/LOC101255991/tXET-B1/LOC101261539/LOC101252007/LOC101258278/LOC100134910/LOC101248295/LOC101254610/XTH3/LOC101255377/LOC101263837/LOC101245676/LOC101260024/LOC101251579/LOC101246362/GBSS1/LOC101266782/LOC101258926/LOC101256480/GGP/LOC101263304/LOC101258941/LOC101260885/LOC101263151/LOC101248301/LOC101259856/LOC101268254/LOC101246863/LOC101256305/LOC101258345/LOC101247596/LOC101254109/tXET-B2/LOC101248301/XTH7/LOC101258632/LOC101254402/LOC101248382/LOC101265706/LOC101244489/LOC101250468/LOC101262856/LOC101256456/LOC101257156/GAME1/LOC543637/XET2/LOC101267720/LOC101261675/LOC101254539/LOC101244316/LOC101261977/Solyc09g008320.5.1/LOC101260093/LOC101248888/LOC101246281/LOC101248084/LOC101267672/LOC101261193/LOC101261784/LOC101261784/LOC101253738/XTH16 |
| MF | GO:0016798 | hydrolase activity, acting on glycosyl bonds | 1.0E-03 | 2.1E-02 | LOC101260664/LOC101251494/TBG7/LOC101250269/LOC101264677/LOC101265391/LOC101252138/LOC101268420/LOC101255416/LOC101248218/LOC101249430/LOC100191128/LOC101249542/LOC101258638/LOC101245995/LOC101267699/LOC101261972/LOC104645427/tXET-B1/LOC101256971/LOC101248093/LOC101257526/XTH6/LOC101259605/LOC101258612/LOC101258581/LOC101261689/LOC101257181/Solyc10g085220.3.1/XTH3/LOC101264974/LOC101255913/TBG5/PGcat/LOC101245376/LOC101253815/LOC101258926/XOPG1/LOC101246571/Solyc04g077190.4.1/SlArf/Xyl4/cel7/LOC101263456/LOC101258345/LOC101265693/AGAL/tXET-B2/LOC101254849/XTH7/LOC104645804/LOC101258632/Cel2/LOC101258163/LOC101244370/LOC101259149/LOC101256554/Cel8/LOC101254239/LOC101254426/LOC101253853/LOC101256456/LOC101249056/TBG6/LOC543637/XET2/LOC543698/SlArf/Xyl2/Solyc09g008320.5.1/LOC101267141/LOC101244887/LOC101268410/LOC101261784/LOC101253328/LOC101261784/LOC101255272/XTH16 |
| MF | GO:0004553 | hydrolase activity, hydrolyzing O-glycosyl compounds | 1.0E-03 | 2.1E-02 | LOC101260664/LOC101251494/TBG7/LOC101250269/LOC101264677/LOC101265391/LOC101252138/LOC101268420/LOC101255416/LOC101248218/LOC101249430/LOC100191128/LOC101249542/LOC101245995/LOC101267699/LOC101261972/LOC104645427/tXET-B1/LOC101256971/LOC101248093/LOC101257526/XTH6/LOC101259605/LOC101258612/LOC101258581/LOC101261689/XTH3/LOC101264974/LOC101255913/TBG5/PGcat/LOC101245376/LOC101253815/LOC101258926/XOPG1/LOC101246571/Solyc04g077190.4.1/SlArf/Xyl4/cel7/LOC101263456/LOC101258345/LOC101265693/AGAL/tXET-B2/LOC101254849/XTH7/LOC104645804/LOC101258632/Cel2/LOC101258163/LOC101244370/LOC101259149/LOC101256554/Cel8/LOC101254239/LOC101254426/LOC101253853/LOC101256456/LOC101249056/TBG6/LOC543637/XET2/LOC543698/SlArf/Xyl2/Solyc09g008320.5.1/LOC101267141/LOC101244887/LOC101268410/LOC101261784/LOC101253328/LOC101261784/LOC101255272/XTH16 |
| MF | GO:0098772 | molecular function regulator | 1.0E-03 | 2.1E-02 | LOC101256509/LOC101262903/LOC101249771/LOC101260787/LOC101249543/LOC109119847/LOC101248784/LOC101243734/PMEU1/LOC101251179/LOC101254166/LOC101244503/LOC101247257/ARPI/LOC101248400/LOC101248367/LOC101260941/LOC101261426/LOC101245743/LOC101258375/LOC101247557/LOC544001/LOC101261415/LOC101255775/LOC101247960/LOC101255652 |
| MF | GO:0008092 | cytoskeletal protein binding | 1.0E-03 | 2.1E-02 | LOC101249258/Solyc11g062370.1.1/LOC101267960/LOC101260706/LOC101261948/LOC101055514/LOC101243980/LOC101256016/LOC101260217/LOC101259004/LOC101247217/LOC101255723/LOC101252434/LOC101055604/LOC101264685/LOC101259830/LOC101247848/LOC101268334/LOC101263791/LOC101262096/LOC101255832/LOC101257576/LOC101258682/LOC101255070/LOC101247910/LOC101260697/LOC101263097/LOC101255427/LOC101261343/LOC101264814/LOC101252039/LOC101257270/LOC101260250/LOC101268475/LOC101264563/LOC101265093/LOC101246423/LOC101255463/LOC101244780/LOC101246993/TKR/LOC101264370/LOC101264472 |
| MF | GO:0008236 | serine-type peptidase activity | 1.0E-03 | 2.1E-02 | P69A/LOC101258381/LOC101262630/LOC101268211/LOC101266188/LOC101252078/sbt3/LOC101267161/sbt4a/LOC101250880/P69B/LOC101250650/LOC544223/LOC101247625/LOC101251209/LOC101245035/LOC101251304/LOC101244564/LOC101249996/LOC101258166/LOC101260269/LOC101252447/LOC101266183/LOC101249160/LOC101260592/LOC101250043/LOC101260044/SBT2/LOC101267900/LOC101251103/LOC101255349/SBT1 |
| MF | GO:0017171 | serine hydrolase activity | 1.0E-03 | 2.1E-02 | P69A/LOC101258381/LOC101262630/LOC101268211/LOC101266188/LOC101252078/sbt3/LOC101267161/sbt4a/LOC101250880/P69B/LOC101250650/LOC544223/LOC101247625/LOC101251209/LOC101245035/LOC101251304/LOC101244564/LOC101249996/LOC101258166/LOC101260269/LOC101252447/LOC101266183/LOC101249160/LOC101260592/LOC101250043/LOC101260044/SBT2/LOC101267900/LOC101251103/LOC101255349/SBT1 |
| MF | GO:0030234 | enzyme regulator activity | 1.0E-03 | 2.1E-02 | LOC109119847/LOC101248784/LOC101243734/PMEU1/LOC101254166/LOC101244503/LOC101247257/ARPI/LOC101248367/LOC101260941/LOC101261426/LOC101245743/LOC101258375/LOC101247557/LOC544001/LOC101261415/LOC101255775/LOC101247960/LOC101255652 |
| MF | GO:0015631 | tubulin binding | 1.1E-03 | 2.1E-02 | LOC101249258/LOC101267960/LOC101260706/LOC101261948/LOC101055514/LOC101256016/LOC101260217/LOC101259004/LOC101247217/LOC101255723/LOC101055604/LOC101259830/LOC101247848/LOC101268334/LOC101263791/LOC101262096/LOC101255832/LOC101258682/LOC101255070/LOC101247910/LOC101260697/LOC101263097/LOC101255427/LOC101261343/LOC101264814/LOC101252039/LOC101257270/LOC101260250/LOC101268475/LOC101264563/LOC101265093/LOC101246423/LOC101255463/LOC101246993/TKR/LOC101264370/LOC101264472 |
| MF | GO:0008017 | microtubule binding | 1.1E-03 | 2.1E-02 | LOC101249258/LOC101267960/LOC101260706/LOC101261948/LOC101055514/LOC101256016/LOC101260217/LOC101259004/LOC101247217/LOC101255723/LOC101055604/LOC101259830/LOC101247848/LOC101268334/LOC101263791/LOC101262096/LOC101255832/LOC101258682/LOC101255070/LOC101247910/LOC101260697/LOC101263097/LOC101255427/LOC101261343/LOC101264814/LOC101252039/LOC101257270/LOC101260250/LOC101268475/LOC101264563/LOC101265093/LOC101246423/LOC101255463/LOC101246993/TKR/LOC101264370/LOC101264472 |
| MF | GO:0046527 | glucosyltransferase activity | 1.1E-03 | 2.1E-02 | LOC101255416/LOC101259456/LOC101252941/tXET-B1/LOC101261539/LOC101252007/LOC100134910/XTH3/LOC101255377/LOC101263837/LOC101260024/GBSS1/LOC101266782/LOC101258926/LOC101246863/LOC101258345/LOC101247596/tXET-B2/XTH7/LOC101258632/LOC101248382/LOC101262856/LOC101256456/LOC543637/XET2/LOC101267720/Solyc09g008320.5.1/LOC101248888/LOC101246281/LOC101267672/LOC101261784/LOC101261784/XTH16 |
| MF | GO:0003774 | motor activity | 1.1E-03 | 2.1E-02 | LOC101267960/LOC101260706/LOC101260217/LOC101259004/LOC101247848/LOC101268334/LOC101262096/LOC101255832/LOC101258682/LOC101255070/LOC101247910/LOC101260697/LOC101263097/LOC101255427/LOC101264152/LOC101261343/LOC101252039/LOC101257270/LOC101260250/LOC101268475/LOC101264563/LOC101265093/LOC101246423/LOC101255463/TKR/LOC101264370/LOC101264472 |
| MF | GO:0003777 | microtubule motor activity | 1.2E-03 | 2.1E-02 | LOC101267960/LOC101260706/LOC101260217/LOC101259004/LOC101247848/LOC101268334/LOC101262096/LOC101255832/LOC101258682/LOC101255070/LOC101247910/LOC101260697/LOC101263097/LOC101255427/LOC101261343/LOC101252039/LOC101257270/LOC101260250/LOC101268475/LOC101264563/LOC101265093/LOC101246423/LOC101255463/TKR/LOC101264370/LOC101264472 |
| MF | GO:0004857 | enzyme inhibitor activity | 1.2E-03 | 2.1E-02 | LOC109119847/LOC101248784/LOC101243734/PMEU1/LOC101254166/LOC101244503/LOC101247257/ARPI/LOC101248367/LOC101260941/LOC101261426/LOC101245743/LOC101258375/LOC101247557/LOC544001/LOC101261415/LOC101255775/LOC101247960/LOC101255652 |
| MF | GO:0005507 | copper ion binding | 1.2E-03 | 2.1E-02 | AO/LOC101256306/LOC101255529/LOC101263571/LOC101249981/LOC101255059/LOC101252344/LOC101266123/LOC101262977/LOC101247502/LOC101247352/LOC101256015/LOC778302/LOC101258916/LOC101253311 |
| MF | GO:0016762 | xyloglucan:xyloglucosyl transferase activity | 1.3E-03 | 2.1E-02 | XTH3/LOC101258926/LOC101258345/tXET-B2/XTH7/LOC101258632/LOC101256456/LOC543637/XET2/Solyc09g008320.5.1/LOC101261784/LOC101261784/XTH16 |
| MF | GO:0030599 | pectinesterase activity | 1.3E-03 | 2.1E-02 | LOC101260787/PMEU1/LOC101260941/LOC101261426/LOC101245743/LOC101258375/LOC101261743/LOC101261415/LOC101255775/LOC101247960 |
| MF | GO:0061134 | peptidase regulator activity | 1.3E-03 | 2.1E-02 | LOC101262903/LOC109119847/LOC101248784/LOC101247257/ARPI/LOC101247557/LOC544001/LOC101255652 |
| MF | GO:0046983 | protein dimerization activity | 2.1E-03 | 3.1E-02 | LOC101263309/LOC101264638/LOC101246034/LOC101259956/PHYB1/LOC101267690/LOC101251452/LOC101265273/LOC101252303/Solyc06g074780.1.1/TDR6/LOC544102/LOC101265602/LOC101259435/LOC101264668/LOC101267024/LOC101250441/H2B-3/LOC101256806/LOC101266819/LOC101251469/Solyc03g071620.2.1/MYC1/MYC2/LOC101258908/LOC101265996/LOC101263596/LOC101253792/LOC101265669/LOC101248685/LOC101268615/LOC101252717/LOC101267164/LOC101268319/LOC101251240/Solyc01g086820.5.1/LOC101267861/LOC101248972/LOC101252889/LOC101252889/LOC101247399/LOC101264068/H2B-2/LOC544081/LOC101254527/LOC101252729/LOC101266736/LOC101260571/FYFL/HTA6/LOC101250929/H2B-1/LOC101245202 |
| MF | GO:0046982 | protein heterodimerization activity | 2.3E-03 | 3.1E-02 | LOC101267024/H2B-3/LOC101256806/Solyc03g071620.2.1/LOC101265996/LOC101253792/LOC101265669/LOC101252717/Solyc01g086820.5.1/LOC101267861/LOC101252889/LOC101252889/H2B-2/LOC544081/LOC101260571/HTA6/LOC101250929/H2B-1 |
| MF | GO:0052689 | carboxylic ester hydrolase activity | 2.4E-03 | 3.1E-02 | LOC101245561/LOC101260787/PMEU1/LOC101260941/LOC101249701/LOC101261426/LOC101245743/LOC101258375/LOC101261743/LOC101261415/LOC101255775/LOC101247960 |
| MF | GO:0004185 | serine-type carboxypeptidase activity | 2.5E-03 | 3.1E-02 | LOC101252078/LOC101250880/LOC544223/LOC101247625/LOC101251209/LOC101244564/LOC101258166/LOC101260269/LOC101260592/LOC101250043/LOC101267900/LOC101251103 |
| MF | GO:0004866 | endopeptidase inhibitor activity | 2.7E-03 | 3.1E-02 | LOC101262903/LOC109119847/LOC101248784/LOC101247257/ARPI/LOC101247557/LOC544001/LOC101255652 |
| MF | GO:0030414 | peptidase inhibitor activity | 2.7E-03 | 3.1E-02 | LOC101262903/LOC109119847/LOC101248784/LOC101247257/ARPI/LOC101247557/LOC544001/LOC101255652 |
| MF | GO:0061135 | endopeptidase regulator activity | 2.7E-03 | 3.1E-02 | LOC101262903/LOC109119847/LOC101248784/LOC101247257/ARPI/LOC101247557/LOC544001/LOC101255652 |
| MF | GO:0070008 | serine-type exopeptidase activity | 3.7E-03 | 3.9E-02 | LOC101252078/LOC101250880/LOC544223/LOC101247625/LOC101251209/LOC101244564/LOC101258166/LOC101260269/LOC101260592/LOC101250043/LOC101267900/LOC101251103 |
| MF | GO:0004180 | carboxypeptidase activity | 3.7E-03 | 3.9E-02 | LOC101252078/LOC101250880/LOC544223/LOC101247625/LOC101251209/LOC101244564/LOC101258166/LOC101260269/LOC101260592/LOC101250043/LOC101267900/LOC101251103 |
| MF | GO:0005516 | calmodulin binding | 3.8E-03 | 4.0E-02 | LOC101253931/LOC101256576/LOC101255950/LOC101248759/LOC101268806/LOC101262099/LOC101260388/Solyc03g113980.3.1/LOC101255381/LOC101267354 |
| MF | GO:0003678 | DNA helicase activity | 4.0E-03 | 4.0E-02 | LOC101260976/LOC101244828/LOC101264867/LOC101243636 |
| MF | GO:0016759 | cellulose synthase activity | 5.2E-03 | 4.9E-02 | LOC101259456/LOC101261539/LOC101260024/LOC101246863/LOC101247596/LOC101248382/LOC101262856/LOC101248888/LOC101246281/LOC101267672 |
| MF | GO:0016760 | cellulose synthase (UDP-forming) activity | 5.2E-03 | 4.9E-02 | LOC101259456/LOC101261539/LOC101260024/LOC101246863/LOC101247596/LOC101248382/LOC101262856/LOC101248888/LOC101246281/LOC101267672 |
| BP | GO:0015979 | photosynthesis | 1.0E-03 | 3.3E-02 | Solyc00g500054.1.1/LOC101259747/psaJ/CAB1B/Solyc09g064580.2.1/LOC101251324/psaA/psbB/psaB/Solyc07g032620.3.1/LOC101266666/LOC101245121/CAB5/LOC101248983/LOC101263732/CAB-10A/LOC543978/LOC101255222/Solyc00g500049.1.1/LOC101265555/CAB13/LHCP/LOC101249002/LOC101267774/Cab-1A/CAB9/LOC101257518/CAB11/Cab-3C/LOC101264609/LOC101245880/psbZ/PSBP/PSBR/LOC101259227/PsbQ/LOC101245729/LOC101266426/LOC101244751/CAB-8/LOC101268297/LOC101256723/CAB1B/LOC101251312/LOC101266160/LOC101262255/PSBO/LOC101257186/LOC101253380/CAB12/CAB4/LOC101265617/CAB1B/Cab-1A/LOC101263969/CAB7/LOC101265249/LOC101254767/LOC101252151/LOC101268123/LOC101262299/LOC101245421/LOC101257676 |
| BP | GO:0044262 | cellular carbohydrate metabolic process | 1.1E-03 | 3.3E-02 | LOC101245612/LOC101257290/IPS/LOC101268420/LOC101255416/LOC101259456/LOC101251056/tXET-B1/LOC101261539/LOC101252007/LOC101250326/LOC100134910/XTH3/LOC101255377/LOC101263837/LOC101260024/LOC101249633/LOC101250610/LOC101266782/AgpL1/LOC101258926/agpL3/LOC101246803/LOC101246863/LOC101258345/LOC101247596/tXET-B2/XTH7/LOC101258632/LOC543968/LOC101248382/LOC101262856/LOC101256456/LOC101266408/LOC543637/XET2/LOC101267720/LOC101257020/Solyc09g008320.5.1/LOC101248888/LOC101246281/LOC101267672/LOC101261784/LOC101261784/XTH16 |
| BP | GO:0007017 | microtubule-based process | 1.1E-03 | 3.3E-02 | LOC101249258/LOC101267960/LOC101260706/LOC101261948/LOC101055514/LOC101267542/LOC101251552/LOC101252240/LOC101260217/LOC101259004/LOC101258583/LOC101253952/LOC101259830/LOC101262728/LOC101247848/LOC101268334/LOC101257536/LOC101263791/LOC101262096/LOC101260712/LOC101255832/TUB/LOC101258682/LOC101257849/LOC101255070/LOC101256145/LOC101247910/LOC101260697/LOC101255154/LOC101263097/LOC101255427/LOC101261343/LOC101252039/LOC101257270/LOC101260250/LOC101268475/LOC101264563/LOC101265093/LOC101246423/LOC101255463/LOC101246993/TKR/LOC101264370/LOC101264472 |
| BP | GO:0005976 | polysaccharide metabolic process | 1.1E-03 | 3.3E-02 | LOC101254331/LOC101251494/LOC101257290/LOC101268420/LOC101255416/LOC101259456/tXET-B1/LOC101261539/LOC101252007/XTH3/LOC101263837/LOC101260024/LOC101253815/AgpL1/LOC101258926/LOC101263958/agpL3/LOC101246863/LOC101258345/LOC101247596/tXET-B2/XTH7/LOC101258632/LOC543968/LOC101248382/LOC101262856/LOC101256456/LOC101266408/LOC543637/XET2/LOC101257020/Solyc09g008320.5.1/LOC101248888/LOC101246281/LOC101267672/LOC101261784/LOC101261784/XTH16 |
| BP | GO:0044264 | cellular polysaccharide metabolic process | 1.1E-03 | 3.3E-02 | LOC101252840/LOC101254331/LOC101257290/LOC101268420/LOC101255416/LOC101259456/tXET-B1/LOC101261539/LOC101252007/XTH3/LOC101263837/LOC101260024/AgpL1/LOC101258926/agpL3/LOC101246863/LOC101258345/LOC101247596/tXET-B2/XTH7/LOC101258632/LOC543968/LOC101248382/LOC101262856/LOC101256456/LOC101266408/LOC543637/XET2/LOC101257020/Solyc09g008320.5.1/LOC101248888/LOC101246281/LOC101267672/LOC101261784/LOC101261784/XTH16 |
| BP | GO:0006073 | cellular glucan metabolic process | 1.1E-03 | 3.3E-02 | LOC101254331/LOC101257290/LOC101268420/LOC101255416/LOC101259456/tXET-B1/LOC101261539/LOC101252007/XTH3/LOC101263837/LOC101260024/AgpL1/LOC101258926/agpL3/LOC101246863/LOC101258345/LOC101247596/tXET-B2/XTH7/LOC101258632/LOC543968/LOC101248382/LOC101262856/LOC101256456/LOC101266408/LOC543637/XET2/Solyc09g008320.5.1/LOC101248888/LOC101246281/LOC101267672/LOC101261784/LOC101261784/XTH16 |
| BP | GO:0044042 | glucan metabolic process | 1.1E-03 | 3.3E-02 | LOC101254331/LOC101257290/LOC101268420/LOC101255416/LOC101259456/tXET-B1/LOC101261539/LOC101252007/XTH3/LOC101263837/LOC101260024/AgpL1/LOC101258926/agpL3/LOC101246863/LOC101258345/LOC101247596/tXET-B2/XTH7/LOC101258632/LOC543968/LOC101248382/LOC101262856/LOC101256456/LOC101266408/LOC543637/XET2/Solyc09g008320.5.1/LOC101248888/LOC101246281/LOC101267672/LOC101261784/LOC101261784/XTH16 |
| BP | GO:0019684 | photosynthesis, light reaction | 1.1E-03 | 3.3E-02 | CAB13/LHCP/LOC101249002/LOC101267774/Cab-1A/CAB9/CAB11/Cab-3C/psbZ/LOC101245729/CAB-8/CAB1B/LOC101251312/LOC101262255/PSBO/LOC101257186/LOC101253380/CAB12/CAB4/LOC101265617/CAB1B/Cab-1A/LOC101263969/CAB7/LOC101254767/LOC101252151/LOC101268123 |
| BP | GO:0006928 | movement of cell or subcellular component | 1.2E-03 | 3.3E-02 | LOC101267960/LOC101260706/LOC101260217/LOC101259004/LOC101247848/LOC101268334/LOC101262096/LOC101255832/LOC101258682/LOC101255070/LOC101247910/LOC101260697/LOC101263097/LOC101255427/LOC101261343/LOC101252039/LOC101257270/LOC101260250/LOC101268475/LOC101264563/LOC101265093/LOC101246423/LOC101255463/TKR/LOC101264370/LOC101264472 |
| BP | GO:0007018 | microtubule-based movement | 1.2E-03 | 3.3E-02 | LOC101267960/LOC101260706/LOC101260217/LOC101259004/LOC101247848/LOC101268334/LOC101262096/LOC101255832/LOC101258682/LOC101255070/LOC101247910/LOC101260697/LOC101263097/LOC101255427/LOC101261343/LOC101252039/LOC101257270/LOC101260250/LOC101268475/LOC101264563/LOC101265093/LOC101246423/LOC101255463/TKR/LOC101264370/LOC101264472 |
| BP | GO:0000271 | polysaccharide biosynthetic process | 1.2E-03 | 3.3E-02 | LOC101252840/LOC101254331/LOC101257290/LOC101268420/LOC101259456/LOC101261539/LOC101252007/LOC101263837/LOC101260024/AgpL1/LOC101263958/agpL3/LOC101246863/LOC101247596/LOC543968/LOC101248382/LOC101262856/LOC101266408/LOC101257020/LOC101248888/LOC101246281/LOC101267672 |
| BP | GO:0033692 | cellular polysaccharide biosynthetic process | 1.2E-03 | 3.3E-02 | LOC101252840/LOC101254331/LOC101257290/LOC101268420/LOC101259456/LOC101261539/LOC101252007/LOC101263837/LOC101260024/AgpL1/agpL3/LOC101246863/LOC101247596/LOC543968/LOC101248382/LOC101262856/LOC101266408/LOC101257020/LOC101248888/LOC101246281/LOC101267672 |
| BP | GO:0009250 | glucan biosynthetic process | 1.2E-03 | 3.3E-02 | LOC101254331/LOC101257290/LOC101268420/LOC101259456/LOC101261539/LOC101252007/LOC101263837/LOC101260024/AgpL1/agpL3/LOC101246863/LOC101247596/LOC543968/LOC101248382/LOC101262856/LOC101266408/LOC101248888/LOC101246281/LOC101267672 |
| BP | GO:0006260 | DNA replication | 1.2E-03 | 3.3E-02 | LOC101254741/Solyc09g010980.1.1/LOC101260976/LOC101244828/DNApolalpha/LOC101268250/LOC101260396/LOC101264867/LOC101256302/LOC101249065/LOC101243636 |
| BP | GO:0009765 | photosynthesis, light harvesting | 1.2E-03 | 3.3E-02 | CAB-10A/CAB13/LHCP/LOC101249002/LOC101267774/Cab-1A/CAB9/CAB11/Cab-3C/LOC101245729/CAB-8/CAB1B/LOC101257186/LOC101253380/CAB12/CAB4/LOC101265617/CAB1B/Cab-1A/LOC101263969/CAB7/LOC101252151/LOC101268123 |
| BP | GO:0045229 | external encapsulating structure organization | 1.2E-03 | 3.3E-02 | LOC101268168/LOC101267739/EXPA4/LOC101260787/COBRA-like/PMEU1/LOC543795/exp18/LOC101260941/LOC101261426/LOC101245743/LOC101258375/LOC101261743/EXPA5/LOC101261415/LOC101255775/LOC101247960 |
| BP | GO:0071555 | cell wall organization | 1.2E-03 | 3.3E-02 | LOC101268168/LOC101267739/EXPA4/LOC101260787/COBRA-like/PMEU1/LOC543795/exp18/LOC101260941/LOC101261426/LOC101245743/LOC101258375/LOC101261743/EXPA5/LOC101261415/LOC101255775/LOC101247960 |
| BP | GO:0042546 | cell wall biogenesis | 1.2E-03 | 3.3E-02 | LOC101255416/LOC101267739/tXET-B1/XTH3/COBRA-like/LOC101258345/tXET-B2/XTH7/LOC101258632/LOC101256456/LOC543637/XET2/LOC101257020/Solyc09g008320.5.1/LOC101261784/LOC101261784/XTH16 |
| BP | GO:0010383 | cell wall polysaccharide metabolic process | 1.3E-03 | 3.3E-02 | XTH3/LOC101258345/tXET-B2/XTH7/LOC101258632/LOC101256456/LOC543637/XET2/LOC101257020/Solyc09g008320.5.1/LOC101261784/LOC101261784/XTH16 |
| BP | GO:0010410 | hemicellulose metabolic process | 1.3E-03 | 3.3E-02 | XTH3/LOC101258345/tXET-B2/XTH7/LOC101258632/LOC101256456/LOC543637/XET2/LOC101257020/Solyc09g008320.5.1/LOC101261784/LOC101261784/XTH16 |
| BP | GO:0010411 | xyloglucan metabolic process | 1.3E-03 | 3.3E-02 | XTH3/LOC101258345/tXET-B2/XTH7/LOC101258632/LOC101256456/LOC543637/XET2/Solyc09g008320.5.1/LOC101261784/LOC101261784/XTH16 |
| BP | GO:0042545 | cell wall modification | 1.3E-03 | 3.3E-02 | LOC101260787/PMEU1/LOC101260941/LOC101261426/LOC101245743/LOC101258375/LOC101261743/LOC101261415/LOC101255775/LOC101247960 |

Supplementary Table 3 Enriched KEGG pathways (*C*Lso haplotype B vs. negative).

| **Method** | **KEGG ID** | **Pathway** | **P Value** | **Adjusted P Value** | **Core Gene** |
| --- | --- | --- | --- | --- | --- |
| Over Representation | sly00040 | Pentose and glucuronate interconversions | 3.8E-06 | 1.4E-04 | LOC101261743/LOC101247960/LOC101263958/LOC101260911/SlPL/LOC101261402/PMEU1/LOC101252082/LOC101244298/LOC101266649/LOC101261426/LOC101263458 |
| Gene Set Enrichment | sly00040 | Pentose and glucuronate interconversions | 1.2E-03 | 1.5E-02 | LOC101260787/PGcat/LOC101263958/XOPG1/PMEU1/SlPL/LOC101244298/LOC101260941/LOC101252082/LOC101261426/LOC101263458/LOC101261743/LOC101260911/LOC101261402/LOC101247960 |
| Over Representation | sly00250 | Alanine, aspartate and glutamate metabolism | 6.4E-04 | 8.1E-03 | GAD3/GS2/LOC101254281/LOC101245157/LOC101260239/SlADH12A1/GABA-TP1/LOC101260072/LOC101268729/GABA-TP2 |
| Over Representation | sly00280 | Valine, leucine and isoleucine degradation | 4.1E-03 | 3.3E-02 | LOC101265771/LOC101262668/LOC101252968/LOC101267526/LOC101249394/LOC543993/LOC101255924/LOC101268729/LOC101252846 |
| Over Representation | sly00330 | Arginine and proline metabolism | 4.2E-03 | 3.3E-02 | LOC101253022/ARG2/PDH/LOC101268445/LOC101255673/LOC101252968/LOC101244293/SlADH12A1/LOC101244015/LOC101260072 |
| Over Representation | sly00350 | Tyrosine metabolism | 4.6E-04 | 7.0E-03 | LOC101248052/LOC101266123/LOC101257377/ADH2/LOC101260072/LOC101259357/LOC101259064/PPO/LOC543605 |
| Over Representation | sly00360 | Phenylalanine metabolism | 4.8E-03 | 3.3E-02 | LOC101266123/LOC101257377/LOC101260072/LOC101264847/AADC1B/LOC101243631/PAL3 |
| Over Representation | sly00500 | Starch and sucrose metabolism | 7.6E-03 | 4.5E-02 | agpL3/LOC104645804/LOC101255272/LOC101246803/LOC543698/LOC101262611/LOC101244370/LOC101257661/TPS1/LOC101267720/sus3/LOC543968/Cel8/Cel2/SUS4/LOC101256554 |
| Over Representation | sly00520 | Amino sugar and nucleotide sugar metabolism | 4.3E-03 | 3.3E-02 | agpL3/CHI17/LOC101263958/LOC101261402/LOC101245851/LOC543968/LOC101249663/LOC101245341/LOC101254509/LOC101262856/LOC101247369/LOC101267358/CHI9/LOC101253853/LOC101244155 |
| Over Representation | sly00592 | alpha-Linolenic acid metabolism | 1.3E-03 | 1.4E-02 | AOC/aos/ADH2/LOC101261255/LOC101255924/LOC101248057/LOC101251665/LOC101263592 |
| Over Representation | sly00940 | Phenylpropanoid biosynthesis | 2.6E-06 | 1.4E-04 | LOC101261193/LOC101249426/LOC101265606/TMP1/LOC104645804/LOC101244246/LOC101255272/LOC101251363/LOC543698/LOC101253684/LOC101243631/PAL3/LOC101249696/LOC101249706/LOC101256554/LOC101266599/LOC101260610/LOC100191129 |
| Gene Set Enrichment | sly00940 | Phenylpropanoid biosynthesis | 1.1E-03 | 1.5E-02 | LOC101266208/LOC101257526/LOC101262367/LOC101266953/PAL3/CCR2/LOC101254287/LOC104648161/CEVI-1/LOC101263035/LOC101244246/LOC101243631/LOC101250523/AnthOMT/LOC101267473/LOC104645804/LOC104648251/LOC101256554/LOC101266599/LOC101265606/LOC101254239/LOC101249426/LOC101251363/LOC112940337/LOC543698/LOC100191129/LOC101249696/LOC101261193/LOC101260610/LOC101255272 |
| Over Representation | sly00941 | Flavonoid biosynthesis | 1.0E-05 | 2.7E-04 | F3H/LOC544150/CHI1/LOC101266223/CHS2/LOC101251607/LOC101249696/LOC101249699/LOC101260610/LOC100191129 |
| Gene Set Enrichment | sly00941 | Flavonoid biosynthesis | 1.2E-03 | 1.5E-02 | LOC101262367/LOC101266953/LOC104648161/AnthOMT/LOC101266618/LOC101249699/LOC104648251/LOC101251607/LOC544150/LOC101266223/CHS1/LOC100191129/CHI1/F3H/LOC101249696/LOC101260610/CHS2 |
| Over Representation | sly00950 | Isoquinoline alkaloid biosynthesis | 7.7E-03 | 4.5E-02 | LOC101266123/LOC101260072/LOC101259357/LOC101259064/PPO |
| Over Representation | sly04075 | Plant hormone signal transduction | 1.7E-04 | 3.3E-03 | SlIAA16/LOC101267355/LOC101263766/LOC101260897/LOC101268544/LOC101258345/LOC101258632/PP2C-2/SRG1/IAA10/ETR4/IAA7/LOC101248690/LOC101261835/LOC101247936/tXET-B2/SlLAX1/IAA3/LOC101055547/LOC101258495/Prg1/SlIAA8/LOC101263193/LOC101253982 |
| Gene Set Enrichment | sly00100 | Steroid biosynthesis | 1.2E-03 | 1.5E-02 | LOC112940012/LOC101252433/CAS1/LOC101250012/LOC101259646/LOC100316899/LOC101264777/CYC1/SlSSR1/LOC101256443/FK/LOC104649012/LOC101256596/LOC101247376/LOC101244831/LOC101258266/CYP51 |
| Gene Set Enrichment | sly00195 | Photosynthesis | 1.1E-03 | 1.5E-02 | LOC101259494/psaA/LOC101243864/psbB/LOC109120519/psaB/LOC101266666/LOC101245121/LOC101249084/LOC101248983/LOC101263732/atpB/atpF/atpE/LOC543978/LOC101255222/atpA/LOC101265555/LOC101245166/PETE/SEND33/LOC101264609/LOC101245880/psbZ/PSBP/PSBR/LOC101259227/PsbQ/LOC101266426/LOC101244751/LOC101268297/LOC101254882/LOC101261284/psbS/PSBO/LOC101253342/LOC101254806/LOC101265784/LOC101265249/LOC101263124/LOC101245421/LOC101257676 |
| Gene Set Enrichment | sly00196 | Photosynthesis - antenna proteins | 1.2E-03 | 1.5E-02 | CAB-10A/CAB13/LHCP/LOC101249002/LOC101267774/CAB9/CAB11/CAB1B/Cab-3C/LOC101245729/CAB-8/Cab-1A/LOC101257186/LOC101253380/CAB12/CAB4/LOC101265617/LOC101263969/CAB7/LOC101252151/LOC101268123 |
| Gene Set Enrichment | sly00908 | Zeatin biosynthesis | 1.3E-03 | 1.5E-02 | LOC101263304/LOC101258941/LOC101268254/LOC101248301/LOC101254402/LOC101265706/LOC101261675/LOC101253738 |
| Gene Set Enrichment | sly00945 | Stilbenoid, diarylheptanoid and gingerol biosynthesis | 1.3E-03 | 1.5E-02 | LOC101265977/LOC101246092/LOC101262367/LOC101268615/LOC101266953/LOC104648161/AnthOMT/LOC104648251/LOC100191129/LOC101249696/LOC101260610 |
| Gene Set Enrichment | sly03030 | DNA replication | 1.3E-03 | 1.5E-02 | LOC101244828/DNApolalpha/LOC101268250/LOC101260396/LOC101264867/LOC101256302/LOC101243636 |

Supplementary Table 4 qPCR primer design for differentially expressed genes.

UID is unique identifier of gene in the tomato ITAG4.1 reference transcriptome. Name is unofficial abbreviation of UID, used only in primer design. Fwd is forward primer. Rev is reverse primer. Selected means whether the primer set is selected for qPCR. A selected primer set was the best among other primers of the same gene based on PCR tests. A gene can have no selected primer set when all primer sets failed. Melt. Temp. is the melting temperature in Celsius. *Internal control genes: no expression difference between plants with or without *C*Lso infection.

| UID | Name | Name Fwd | Name Rev | Sele-cted | Frag-ment length | Sequence Fwd | Melt. Temp. Fwd | Sequence Rev | Melt. Temp. Rev |
| --- | --- | --- | --- | --- | --- | --- | --- | --- | --- |
| Solyc05g007070.2.1 | AA3 | AA3-F1 | AA3-R1 |  | 143 | TAATTTCCATGCTGCTCCT | 54.5 | CATAACCACCCCAAAACC | 54.7 |
| Solyc05g007070.2.1 | AA3 | AA3-F2 | AA3-R1 |  | 127 | CCTAACATTGATCATTCCC | 53.6 | CATAACCACCCCAAAACC | 54.7 |
| Solyc05g007070.2.1 | AA3 | AA3-F3 | AA3-R1 | ✓ | 189 | CAGATGATCCACATTTCC | 51.9 | CATAACCACCCCAAAACC | 54.7 |
| Solyc08g068330.4.1 | AAC | AAC-F1 | AAC-R1 |  | 95 | ATCCTATTCTCGGCGTTA | 52.3 | TTTTCCTTCCTCTGTCCT | 53.3 |
| Solyc08g068330.4.1 | AAC | AAC-F2 | AAC-R1 |  | 130 | GTCTGTTTTCTCTCATATCG | 53.6 | TTTTCCTTCCTCTGTCCT | 53.3 |
| Solyc08g068330.4.1 | AAC | AAC-F3 | AAC-R1 | ✓ | 270 | ACACAACAATCACCGTCA | 54.2 | TTTTCCTTCCTCTGTCCT | 53.3 |
| Solyc11g005330.2.1* | ACT51 | ACT51-F1 | ACT51-R1 | ✓ | 208 | AAGGCCAACAGAGAGAAA | 54.9 | CAGCAAGATCCAAACGAA | 53.7 |
| Solyc11g005330.2.1* | ACT51 | ACT51-F2 | ACT51-R2 |  | 132 | GATATTCAGCCCCTTGTTT | 54.4 | CATTCCAACCATAACACCA | 53.4 |
| Solyc11g005330.2.1* | ACT51 | ACT51-F3 | ACT51-R3 |  | 163 | TAAGGCCAACAGAGAGAA | 52.4 | GTAAATAGGGACAGTGTGA | 49.7 |
| Solyc02g069250.5.1 | ADS | ADS-F1 | ADS-R1 | ✓ | 199 | GGATAAAGTAGGAGTTGG | 51.3 | GGGTAGGTTATTTGGTATAG | 52.2 |
| Solyc02g069250.5.1 | ADS | ADS-F2 | ADS-R2 |  | 138 | CTATACCAAATAACCTACCC | 51.2 | CTAATCCTCCCAAACCAA | 53.1 |
| Solyc02g069250.5.1 | ADS | ADS-F3 | ADS-R3 |  | 295 | CTGTTATGTGCAGGAGTA | 51.8 | GAGAGTGATTGGCTGAGA | 52.9 |
| Solyc09g009190.5.1 | AGBE22 | AGBE22-F1 | AGBE22-R1 | ✓ | 245 | GCTTCATCACTACAACTACA | 54.1 | TCCAAACCACCCTCATAC | 55.3 |
| Solyc09g009190.5.1 | AGBE22 | AGBE22-F2 | AGBE22-R2 |  | 175 | TCTTATTCACGGGCTTTTC | 53.4 | AACTCTCCAATCCTCATC | 52.8 |
| Solyc09g009190.5.1 | AGBE22 | AGBE22-F3 | AGBE22-R3 |  | 123 | CGTTTTGTATTCCCGTTC | 53.4 | CAATATCACCAACTCTCC | 51.2 |
| Solyc01g079790.5.1 | AGPL3 | AGPL3-F1 | AGPL3-R1 | ✓ | 204 | ATGGAGAATTGATGGGGAAGA | 57.7 | TGAGGGGGAAAAGACGAG | 57.9 |
| Solyc01g079790.5.1 | AGPL3 | AGPL3-F2 | AGPL3-R2 |  | 206 | GGAGAATTGATGGGGAAG | 54.7 | TTGGTGAGGGGGAAAAGA | 54.3 |
| Solyc01g079790.5.1 | AGPL3 | AGPL3-F3 | AGPL3-R1 |  | 111 | ATATTGCAGGTGAGGCTAAG | 57.7 | TGAGGGGGAAAAGACGAG | 57.9 |
| Solyc04g077970.5.1* | APRT | APRT-F1 | APRT-R1 | ✓ | 216 | AAAATCACCTACCACCAC | 54.0 | ATGACCCGAATAGCAGAA | 54.0 |
| Solyc04g077970.5.1* | APRT | APRT-F2 | APRT-R2 |  | 172 | GGGAGGTTATTTCAGAAG | 51.3 | ACTACCTCAACTCCAACA | 52.1 |
| Solyc04g077970.5.1* | APRT | APRT-F3 | APRT-R2 |  | 173 | GGGGAGGTTATTTCAGAA | 52.1 | ACTACCTCAACTCCAACA | 52.1 |
| Solyc02g080540.1.1 | ASGC | ASGC-F1 | ASGC-R1 |  | 217 | TCCTAACCACAACCTATC | 51.9 | TACCTCAACCAAAGTCTC | 51.9 |
| Solyc02g080540.1.1 | ASGC | ASGC-F2 | ASGC-R2 |  | 78 | CCATCTCTTTCTGAATCC | 51.2 | TGATAGGTTGTGGTTAGG | 51.6 |
| Solyc02g080540.1.1 | ASGC | ASGC-F3 | ASGC-R3 | ✓ | 231 | CCATCTCTTTCTGAATCCT | 53.2 | TTCTTGTGCTCTTCTGACT | 54.8 |
| Solyc06g065990.1.1 | ASSB | ASSB-F1 | ASSB-R1 | ✓ | 165 | TGGCCAACATGATTATGAG | 53.5 | GAGTTGATGGGATTGAGAG | 54.1 |
| Solyc06g065990.1.1 | ASSB | ASSB-F2 | ASSB-R2 |  | 163 | GGCCAACATGATTATGAG | 53.4 | AGTTGATGGGATTGAGAG | 53.1 |
| Solyc06g065990.1.1 | ASSB | ASSB-F3 | ASSB-R3 |  | 124 | ATGGCCAACATGATTATGAG | 55.3 | TGGGGAGGGGTAGTTTTG | 56.3 |
| Solyc09g091030.3.1 | BAM1 | BAM1-F1 | BAM1-R1 |  | 130 | TGTCTTTCCATCAATGCG | 53.5 | TCAAAATTCCTCCTTCCC | 53.0 |
| Solyc09g091030.3.1 | BAM1 | BAM1-F2 | BAM1-R2 |  | 242 | GTTGAGGAGATGGAGAAG | 53.7 | GGATAGGATGGATAACGGA | 53.2 |
| Solyc09g091030.3.1 | BAM1 | BAM1-F3 | BAM1-R3 | ✓ | 152 | GTGTGGAAGGGATTATGA | 52.7 | ACCGCATTGATGGAAAGA | 54.0 |
| Solyc02g069690.1.1 | BBE15 | BBE15-F1 | BBE15-R1 |  | 105 | GGATGTGGGAGAAGTTTTTA | 52.0 | TATGTGGGAATGGGATTG | 52.3 |
| Solyc02g069690.1.1 | BBE15 | BBE15-F2 | BBE15-R2 | ✓ | 152 | GTCATTATTCCCTGCAGTA | 52.8 | TCTTTCCCATCATTCCTC | 51.4 |
| Solyc02g069690.1.1 | BBE15 | BBE15-F3 | BBE15-R3 |  | 113 | TTTTCTCCGTGTACCAGT | 54.0 | TCTTTCCCATCATTCCTCC | 54.3 |
| Solyc09g065540.3.1 | BCC | BCC-F1 | BCC-R1 | ✓ | 129 | GCGACATCTTATCCTTCT | 53.2 | CTCCATCTTCATTGCTTCT | 53.9 |
| Solyc09g065540.3.1 | BCC | BCC-F1 | BCC-R2 |  | 82 | GCGACATCTTATCCTTCT | 53.2 | CTTTCTCCCCATCCTTCA | 52.2 |
| Solyc09g065540.3.1 | BCC | BCC-F1 | BCC-R3 |  | 142 | GCGACATCTTATCCTTCT | 53.2 | CTTTCACTACATGCTCCA | 53.4 |
| Solyc03g119080.4.1 | BMP | BMP-F1 | BMP-R1 |  | 294 | TCAAGAGAAACAGAAAGGG | 52.7 | GGTGTGGTATAATGAGGG | 53.1 |
| Solyc03g119080.4.1 | BMP | BMP-F2 | BMP-R1 |  | 293 | CAAGAGAAACAGAAAGGGA | 54.4 | GGTGTGGTATAATGAGGG | 53.1 |
| Solyc03g119080.4.1 | BMP | BMP-F3 | BMP-R2 | ✓ | 206 | TGCCCAAAGAGCTAGAGA | 53.1 | GTGTGGTATAATGAGGGT | 52.8 |
| Solyc07g047850.3.1 | CAB4 | CAB4-F1 | CAB4-R1 |  | 154 | CAAACACCATCCTACTTAAC | 53.0 | GGAAAACACAACCTAAAGCA | 53.5 |
| Solyc07g047850.3.1 | CAB4 | CAB4-F2 | CAB4-R1 |  | 68 | TAGAAACCGTGAGCTTGA | 52.9 | GGAAAACACAACCTAAAGCA | 53.5 |
| Solyc07g047850.3.1 | CAB4 | CAB4-F1 | CAB4-R2 | ✓ | 193 | CAAACACCATCCTACTTAAC | 53.0 | CTTCACCGAATTTAACACC | 52.8 |
| Solyc01g107590.3.1 | CAD1 | CAD1-F1 | CAD1-R1 |  | 217 | CTTTCACCTTATACCTACAC | 51.8 | CAACTCCAACTATATCCC | 50.6 |
| Solyc03g116910.3.1 | CCR2 | CCR2-F1 | CCR2-R1 |  | 282 | TCACTGATGATCCAGAAC | 52.1 | TCACAACCAAATCCACTC | 52.7 |
| Solyc03g116910.3.1 | CCR2 | CCR2-F2 | CCR2-R1 | ✓ | 216 | CAGCAGAAACCAAAGTTC | 52.8 | TCACAACCAAATCCACTC | 52.7 |
| Solyc03g116910.3.1 | CCR2 | CCR2-F3 | CCR2-R2 |  | 191 | GTTCGCAGAGTTGTGTTT | 55.5 | TCCACTCCCTTTTCCCTT | 55.7 |
| Solyc08g082250.3.1 | CEL8 | CEL8-F1 | CEL8-R1 |  | 299 | GTACCTTCCTCGTAACCA | 53.5 | TATTACCATCACCAACCTC | 52.4 |
| Solyc08g082250.3.1 | CEL8 | CEL8-F1 | CEL8-R2 |  | 86 | GTACCTTCCTCGTAACCA | 53.5 | CTCCAACCAAATCAATCC | 52.5 |
| Solyc08g082250.3.1 | CEL8 | CEL8-F1 | CEL8-R3 | ✓ | 90 | GTACCTTCCTCGTAACCA | 53.5 | TACCCTCCAACCAAATCA | 52.3 |
| Solyc01g006300.3.1 | CEVI1 | CEVI1-F1 | CEVI1-R1 | ✓ | 244 | TGGAGGATTTGATATTGTGG | 53.4 | CTTGTTGGTGAATTGTGGT | 55.9 |
| Solyc02g082930.3.1 | CHI17 | CHI17-F1 | CHI17-R1 | ✓ | 155 | TGATGATACTGCGCGTAA | 53.9 | CTCTGCCATAAAATCCACT | 54.4 |
| Solyc02g082930.3.1 | CHI17 | CHI17-F2 | CHI17-R1 |  | 123 | CCTTTTTCGGTCAAACTTC | 54.0 | CTCTGCCATAAAATCCACT | 54.4 |
| Solyc02g082930.3.1 | CHI17 | CHI17-F3 | CHI17-R2 |  | 159 | GCAGCCAATTCATTTCCA | 54.9 | GCCTTCCCTAACAAAACA | 54.1 |
| Solyc01g008110.5.1 | CPY51 | CPY51-F1 | CPY51-R1 | ✓ | 237 | GTTGAAGGGCTATGTAGA | 52.7 | ATTGGAAGGTAGGGAAAG | 53.4 |
| Solyc01g008110.5.1 | CPY51 | CPY51-F2 | CPY51-R1 |  | 221 | GATCAGATGGTTACAGAAG | 52.2 | ATTGGAAGGTAGGGAAAG | 53.4 |
| Solyc01g008110.5.1 | CPY51 | CPY51-F3 | CPY51-R1 |  | 291 | TACAATTAGGCAGGAACAG | 53.2 | ATTGGAAGGTAGGGAAAG | 53.4 |
| Solyc02g085020.4.1 | DDR | DDR-F1 | DDR-R1 |  | 179 | CCATCATGCTATCATCTAC | 51.1 | CCCCTTTATACATATCCTC | 50.1 |
| Solyc02g085020.4.1 | DDR | DDR-F2 | DDR-R1 |  | 193 | TTCATTTGCTCATCCCATC | 53.5 | CCCCTTTATACATATCCTC | 50.1 |
| Solyc02g085020.4.1 | DDR | DDR-F3 | DDR-R1 | ✓ | 291 | CTCACTACGGCATCATTA | 52.1 | CCCCTTTATACATATCCTC | 50.1 |
| Solyc02g069490.4.1 | DSR | DSR-F1 | DSR-R1 | ✓ | 262 | CTGATGTTGGTGTGTACT | 53.1 | TTCTCTGTCTTCCTTCCT | 52.9 |
| Solyc02g069490.4.1 | DSR | DSR-F2 | DSR-R2 |  | 148 | TCCTTCCATTCTCGTTCT | 54.1 | TCCTTTTCTGCATTCCTCT | 54.4 |
| Solyc02g069490.4.1 | DSR | DSR-F2 | DSR-R3 |  | 201 | TCCTTCCATTCTCGTTCT | 54.1 | CATTTCTCATTCCAACAACC | 54.0 |
| Solyc10g055800.2.1 | EC4 | EC4-F1 | EC4-R1 |  | 148 | GAACAATGTGGTAGACAG | 52.4 | TAGGAGGTTTCGGAGAAG | 53.5 |
| Solyc10g055800.2.1 | EC4 | EC4-F2 | EC4-R2 |  | 139 | TTGGGTGGTGTGGTAATA | 51.7 | GAATTTGAGATGACGCTG | 53.5 |
| Solyc10g055800.2.1 | EC4 | EC4-F3 | EC4-R2 | ✓ | 220 | TGTTGTTAACTGTCTCGG | 53.9 | GAATTTGAGATGACGCTG | 53.5 |
| Solyc05g005080.3.1 | EG25 | EG25-F1 | EG25-R1 | ✓ | 252 | CCTCCACCAGATAACTACAC | 57.4 | ATACTCAATCACACTCCAACTC | 57.3 |
| Solyc05g005080.3.1 | EG25 | EG25-F2 | EG25-R2 |  | 191 | ACCTAATTTCTACTCCACC | 52.5 | ATTTCCACCCTCCTTTAC | 53.4 |
| Solyc05g005080.3.1 | EG25 | EG25-F3 | EG25-R3 |  | 220 | GTTATGTTGTTGGCTTTGG | 54.8 | GTTGGCTCGGTATAATTGT | 55.0 |
| Solyc02g083810.4.1 | FNR | FNR-F1 | FNR-R1 | ✓ | 197 | GTTTCTCTTCCTTCATCCA | 53.0 | CCTTCCTCCTGTTTCTTT | 53.2 |
| Solyc02g083810.4.1 | FNR | FNR-F2 | FNR-R1 |  | 175 | CCACCTCCTTTTCCTCTA | 54.4 | CCTTCCTCCTGTTTCTTT | 53.2 |
| Solyc02g083810.4.1 | FNR | FNR-F3 | FNR-R2 |  | 209 | TCCTTTCCGTTCATTCCT | 54.2 | TCTTTTCACCCTTTTCGTTC | 53.9 |
| Solyc07g043310.3.1 | GAT1 | GAT1-F1 | GAT1-R1 | ✓ | 188 | TAATGATCCTTTCCCTCC | 51.0 | CTCTCTTTTCAGTTTCCC | 51.7 |
| Solyc07g043310.3.1 | GAT1 | GAT1-F2 | GAT1-R1 |  | 191 | TCCTAATGATCCTTTCCC | 51.2 | CTCTCTTTTCAGTTTCCC | 51.7 |
| Solyc07g043310.3.1 | GAT1 | GAT1-F3 | GAT1-R2 |  | 117 | GATTAGGGGACTTGGTTT | 53.3 | TTCTTCTGACATTGCGCT | 55.6 |
| Solyc08g083320.4.1 | GBSS1 | GBSS1-F1 | GBSS1-R1 |  | 126 | TCCCTCTTCTCAATCTTC | 51.7 | CCACCCTATGTGATTCTA | 50.7 |
| Solyc08g083320.4.1 | GBSS1 | GBSS1-F2 | GBSS1-R2 | ✓ | 132 | AACCATACTCTGACACAC | 53.7 | CATTCCCTTTCCACAAAC | 54.5 |
| Solyc08g083320.4.1 | GBSS1 | GBSS1-F3 | GBSS1-R3 |  | 110 | GGCTCCAATCAAGAACTAA | 53.9 | AACCTCAGTACCCACAAA | 53.9 |
| Solyc07g056140.3.1 | GPA | GPA-F1 | GPA-R1 |  | 110 | AGCTTTTACGACCGATCA | 54.8 | ATCACACAACCTTCACCA | 55.4 |
| Solyc07g056140.3.1 | GPA | GPA-F2 | GPA-R2 |  | 155 | ATTTGGGATGAGAGTGCAAG | 57.9 | GAGGTTGGGTGTAGATTGGG | 57.8 |
| Solyc07g056140.3.1 | GPA | GPA-T2 | GPA-R3 | ✓ | 128 | ATATGATGGGTACTGGGA | 52.6 | GAGGTTGGGTGTAGATTG | 52.7 |
| Solyc02g089440.3.1 | GTF | GTF-F1 | GTF-R1 |  | 201 | AAATACAGGGATGAGGAC | 52.9 | CCTCATCTTAAACCACAC | 51.9 |
| Solyc02g089440.3.1 | GTF | GTF-F2 | GTF-R2 |  | 142 | GGATGATGATGTTGTGGT | 54.9 | AACAAGAGGGTGAGAGAA | 53.8 |
| Solyc02g089440.3.1 | GTF | GTF-F3 | GTF-R3 | ✓ | 201 | TTACTGTTTCTCGCCACT | 55.6 | TTCCAACTTCAGCTTCCTC | 55.6 |
| Solyc04g015270.3.1 | GTF4 | GTF4-F1 | GTF4-R1 | ✓ | 258 | GGAAAGACGAAAGCATAG | 52.7 | CCGAGAGTGAAAGGTAGA | 51.8 |
| Solyc04g015270.3.1 | GTF4 | GTF4-F2 | GTF4-R2 |  | 121 | TGTGCAAAAGGATCTTGG | 54.8 | AGGATTTGAGAAGTTGAGG | 54.9 |
| Solyc04g015270.3.1 | GTF4 | GTF4-F3 | GTF4-R3 |  | 226 | CACGAACCACAACAAACA | 54.3 | CATTCCGCTCTTCATCTT | 54.3 |
| Solyc06g083310.3.1 | GTF8 | GTF8-F1 | GTF8-R1 |  | 190 | TTCTGGCTTATGCTTCCT | 54.3 | CTTTCCTTCACCTCCTTC | 53.9 |
| Solyc06g083310.3.1 | GTF8 | GTF8-F2 | GTF8-R2 | ✓ | 277 | GAAGGAGGTGAAGGAAAG | 53.7 | AAATTCTGGTGGAGTAGG | 53.5 |
| Solyc04g081400.3.1 | HXK4 | HXK4-F1 | HXK4-R1 |  | 75 | CACAAAAGGAAGGTGGTAA | 54.3 | CCGGAAAAGAAAACGTGA | 54.1 |
| Solyc04g081400.3.1 | HXK4 | HXK4-F2 | HXK4-R1 |  | 150 | CTATACCCCAAGAACTGA | 53.2 | CCGGAAAAGAAAACGTGA | 54.1 |
| Solyc04g081400.3.1 | HXK4 | HXK4-F3 | HXK4-R1 | ✓ | 201 | AGCTAGGTGGTAAAGAAGA | 53.3 | CCGGAAAAGAAAACGTGA | 54.1 |
| Solyc11g069180.2.1 | ICD | ICD-F1 | ICD-R1 | ✓ | 140 | CCCATGAGCAGAAACAAAA | 54.1 | AACATAACCACCCTCAAC | 54.3 |
| Solyc11g069180.2.1 | ICD | ICD-F1 | ICD-R1 |  | 140 | CCCATGAGCAGAAACAAAA | 54.1 | AACATAACCACCCTCAAC | 54.3 |
| Solyc11g069180.2.1 | ICD | ICD-F2 | ICD-R2 |  | 237 | GAGAAGGAAATGACAGGA | 51.8 | AACATAGGGGAGAACAAC | 53.5 |
| Solyc04g078460.3.1 | IPA2 | IPA2-F1 | IPA2-R1 |  | 136 | TGGAACAGTGGAAATGGA | 52.6 | GAGGTAAGAATGAGGAGA | 50.5 |
| Solyc04g078460.3.1 | IPA2 | IPA2-F2 | IPA2-R1 |  | 198 | GTGAGAGAACTTGAAAGTG | 53.6 | GAGGTAAGAATGAGGAGA | 50.5 |
| Solyc04g078460.3.1 | IPA2 | IPA2-F3 | IPA2-R2 | ✓ | 108 | TCTCCTCATTCTTACCTC | 49.6 | CATACCAACATTGTCCTC | 51.9 |
| Solyc12g006530.2.1 | ITTS1 | ITTS1-F1 | ITTS1-R1 |  | 69 | GGAGTTTTTGAATGGATAGG | 53.8 | GGGAAGAAAAGATGGAAGAA | 53.5 |
| Solyc12g006530.2.1 | ITTS1 | ITTS1-F2 | ITTS1-R1 | ✓ | 176 | CAGATGGCGGTGTAAATAA | 54.6 | GGGAAGAAAAGATGGAAGAA | 53.5 |
| Solyc12g006530.2.1 | ITTS1 | ITTS1-F3 | ITTS1-R1 |  | 168 | GGTGTAAATAATGCGTGTG | 53.4 | GGGAAGAAAAGATGGAAGAA | 53.5 |
| Solyc08g080040.4.1 | LDX2 | LDX2-F1 | LDX2-R1 |  | 183 | CTTTCATTGTGTTTTCCCC | 54.7 | CCTCCAACTTCCTTCTCT | 54.4 |
| Solyc08g080040.4.1 | LDX2 | LDX2-F2 | LDX2-R1 | ✓ | 136 | CTAAAACCCCTGCTGACT | 55.6 | CCTCCAACTTCCTTCTCT | 54.4 |
| Solyc08g080040.4.1 | LDX2 | LDX2-F3 | LDX2-R1 |  | 122 | GACTACATTCCAGCAACA | 53.7 | CCTCCAACTTCCTTCTCT | 54.4 |
| Solyc06g005750.3.1 | MM22 | MM22-F1 | MM22-R1 |  | 172 | AGGGTGGTTTGGCAAGTA | 55.7 | GGATGGCAATGGAAGAGT | 56.1 |
| Solyc06g005750.3.1 | MM22 | MM22-F2 | MM22-R2 |  | 93 | AGCGCTTTCTTCTTGTCTG | 58.0 | CGGGGTGTTGGTCTTTTT | 57.8 |
| Solyc06g005750.3.1 | MM22 | MM22-F3 | MM22-R1 | ✓ | 218 | GCGCTTTCTTCTTGTCTG | 55.9 | GGATGGCAATGGAAGAGT | 56.1 |
| Solyc09g007890.1.1 | PAL2 | PAL2-F1 | PAL2-R1 | ✓ | 299 | CACTTACCCTTTGATCCA | 51.8 | CCTTTTCAATATCCTCACC | 51.4 |
| Solyc09g007890.1.1 | PAL2 | PAL2-F2 | PAL2-R2 |  | 130 | GTGACTACTGGATTTGGTGA | 54.5 | GTGGCAAAGTATGAGATGA | 54.7 |
| Solyc06g084050.4.1 | PIIRCW | PIIRCW-F1 | PIIRCW-R1 | ✓ | 108 | TTGTACCACCACCTCTTT | 53.7 | CTTCCCACATTCTTCCTC | 53.9 |
| Solyc06g082940.3.1 | PIRC6 | PIRC6-F1 | PIRC6-R1 |  | 208 | GCTGTCCAAGCTGATAAA | 51.6 | CAAAAGGCCCAACTAGTAA | 52.8 |
| Solyc06g082940.3.1 | PIRC6 | PIRC6-F2 | PIRC6-R2 |  | 225 | GTCCATTAAGAAACACAGC | 53.5 | CACCAAAAAAGAATCCACC | 54.0 |
| Solyc06g082940.3.1 | PIRC6 | PIRC6-F3 | PIRC6-R1 | ✓ | 184 | TTCCAAGTGATTCAGCCA | 52.8 | CAAAAGGCCCAACTAGTAA | 52.8 |
| Solyc08g074683.1.1 | PPOB | PPOB-F1 | PPOB-R1 | ✓ | 193 | CTACTCCTATTCCATCCC | 51.7 | GCGATATACTCCTCATCT | 50.9 |
| Solyc08g074683.1.1 | PPOB | PPOB-F2 | PPOB-R2 |  | 94 | GTGTGCAATAGTAGTAGTAG | 52.7 | GAAGGAAAAGTTGAGAGG | 52.3 |
| Solyc08g074683.1.1 | PPOB | PPOB-F3 | PPOB-R3 |  | 195 | CTACTACTACAACGCTCA | 51.3 | CACCATAAAGACCTCCTAAA | 51.0 |
| Solyc08g074620.3.1 | PPOE | PPOE-F1 | PPOE-R1 |  | 176 | CAAATCCCTCTCTTCTTC | 51.0 | GACCTCCTAACCCTAAAA | 50.9 |
| Solyc08g074620.3.1 | PPOE | PPOE-F2 | PPOE-R2 | ✓ | 172 | CCTCTCTTCTTCCTTCAC | 53.1 | AAGACCTCCTAACCCTAA | 52.5 |
| Solyc08g074630.2.1 | PPOF | PPOF-F1 | PPOF-R1 |  | 205 | CTCTTCCTTTATTATCCACC | 51.7 | CCATAAAAACCTCCTAACC | 51.8 |
| Solyc08g074630.2.1 | PPOF | PPOF-F2 | PPOF-R1 | ✓ | 189 | CACCAACAAATCCCTCTC | 54.6 | CCATAAAAACCTCCTAACC | 51.8 |
| Solyc04g071900.5.1 | PSE12 | PSE12-F1 | PSE12-R1 | ✓ | 122 | CTAGGAAGAAGGGATGGA | 50.9 | GTGGGAGTGAAGTTTTTG | 53.5 |
| Solyc04g071900.5.1 | PSE12 | PSE12-F2 | PSE12-R1 |  | 196 | CTTGTGCTGATATTACGG | 54.0 | GTGGGAGTGAAGTTTTTG | 53.5 |
| Solyc07g052510.4.1 | PSE3 | PSE3-F1 | PSE3-R1 |  | 217 | CAGCAGCCATACTCAGAA | 55.9 | ACCAACGCGACAATATCA | 55.7 |
| Solyc07g052510.4.1 | PSE3 | PSE3-F2 | PSE3-R2 |  | 184 | CGGGAGTTGTTTCTTGTG | 55.8 | GCGAAGGATTGTTGTAGT | 53.7 |
| Solyc07g052510.4.1 | PSE3 | PSE3-F3 | PSE3-R3 | ✓ | 174 | GACATTTGATCTCAGCTAC | 52.7 | ACTCTTCCCATTTTCTCC | 53.6 |
| Solyc02g080530.3.1 | PSE42 | PSE42-F1 | PSE42-R1 | ✓ | 126 | AGAAAGAGACAGACAGGA | 52.8 | CTCTACCAGACAACACAA | 53.2 |
| Solyc02g080530.3.1 | PSE42 | PSE42-F2 | PSE42-R2 |  | 251 | CAAACGCCACAAGAATAC | 53.6 | CAACAATACCATCTCTACCA | 52.3 |
| Solyc02g080530.3.1 | PSE42 | PSE42-F3 | PSE42-R3 |  | 88 | TGGTGTTGTTTCTTGTGCTG | 57.7 | AGTTTTGAGAGGAATGTGTGG | 58.0 |
| Solyc06g076630.3.1 | PSE47 | PSE47-F1 | PSE47-R1 | ✓ | 250 | GGTAACACAGCAGAAAAG | 52.6 | TATTAAGTGTGGGAGGAG | 50.6 |
| Solyc06g076630.3.1 | PSE47 | PSE47-F1 | PSE47-R1 |  | 250 | GGTAACACAGCAGAAAAG | 52.6 | TATTAAGTGTGGGAGGAG | 50.6 |
| Solyc06g076630.3.1 | PSE47 | PSE47-F2 | PSE47-R2 |  | 227 | CGTCGGTGTTGATTGATT | 55.3 | TGTTCCATCCTTTCTTCCT | 55.2 |
| Solyc02g087190.1.1 | PSE63 | PSE63-F1 | PSE63-R1 |  | 110 | CAAACAGATCACTTCACC | 52.5 | TGAAAGGAGTGGAGGAGA | 53.0 |
| Solyc02g087190.1.1 | PSE63 | PSE63-F2 | PSE63-R2 |  | 91 | CCTCCGTCTCTTCTTCCA | 54.6 | GTCACGTTCAGCTTTGTT | 54.7 |
| Solyc02g087190.1.1 | PSE63 | PSE63-F3 | PSE63-R1 | ✓ | 182 | GCCGCTAAATACTGCTTA | 53.2 | TGAAAGGAGTGGAGGAGA | 53.0 |
| Solyc02g083490.3.1 | PSE64 | PSE64-F1 | PSE64-R1 |  | 236 | GGGTTGTGATGCTTCTAT | 53.8 | GTCCGTCTTTTCTTCCTT | 53.1 |
| Solyc02g083490.3.1 | PSE64 | PSE64-F2 | PSE64-R2 | ✓ | 150 | GAAGCACAGAAAGACAGA | 52.9 | GTGCAAAGAACCATTAGGA | 52.5 |
| Solyc02g083490.3.1 | PSE64 | PSE64-F3 | PSE64-R1 |  | 282 | TGCCCGCTACACTTCTAA | 55.6 | GTCCGTCTTTTCTTCCTT | 53.1 |
| Solyc05g052280.3.1 | PSEP7 | PSEP7-F1 | PSEP7-R1 |  | 85 | CTGTAGAAAAAGCATGCC | 53.1 | CCTCCAAGAATAACAACC | 51.3 |
| Solyc05g052280.3.1 | PSEP7 | PSEP7-F2 | PSEP7-R2 |  | 109 | GGTTGTTATTCTTGGAGG | 50.7 | GTTAAAAGTAGGAGCTGG | 51.7 |
| Solyc05g052280.3.1 | PSEP7 | PSEP7-F3 | PSEP7-R3 |  | 189 | GTGCTGTTGGCCTTTCTA | 54.4 | GTGGTGCTAAGTTGTTGT | 55.3 |
| Solyc10g044520.3.1 | SEND33 | SEND33-F1 | SEND33-R1 | ✓ | 105 | TAGCACTTCTTTCCTTCC | 52.9 | ATCCTCCCATTTCTACCA | 52.9 |
| Solyc10g044520.3.1 | SEND33 | SEND33-F2 | SEND33-R2 |  | 69 | AGCCTCAAAGCCATATCA | 54.8 | AATCCTCCCATTTCTACC | 52.9 |
| Solyc02g030170.4.1 | SISSR1 | SISSR1-F1 | SISSR1-R1 |  | 281 | ATGCTTGTTCCTCTCTAC | 53.3 | TCAATCAACCAACTCTCC | 52.5 |
| Solyc02g030170.4.1 | SISSR1 | SISSR1-F2 | SISSR1-R1 |  | 97 | CTGATGTTGGTGTCTACT | 52.5 | TCAATCAACCAACTCTCC | 52.5 |
| Solyc02g030170.4.1 | SISSR1 | SISSR1-F2 | SISSR1-R2 | ✓ | 263 | CTGATGTTGGTGTCTACT | 52.5 | CTTCTCTGTCTTCTTTCCT | 53.3 |
| Solyc04g077440.4.1 | SM | SM-F1 | SM-R1 |  | 109 | TCTTCGGATCTCTTCTTGT | 53.6 | GTTGTAGTGGTGCTAGTT | 53.8 |
| Solyc04g077440.4.1 | SM | SM-F2 | SM-R1 |  | 123 | GGTGGATTTAGGTCTCTT | 53.5 | GTTGTAGTGGTGCTAGTT | 53.8 |
| Solyc04g077440.4.1 | SM | SM-F1 | SM-R2 | ✓ | 155 | TCTTCGGATCTCTTCTTGT | 53.6 | GTCTTCGTTTCCATCCTT | 53.2 |
| Solyc02g079500.4.1 | TMP1 | TMP1-F1 | TMP1-R1 | ✓ | 144 | TTGATGGTTGTGATGGAG | 52.8 | TGGGGCATGTGTTTATGA | 52.9 |
| Solyc02g079500.4.1 | TMP1 | TMP1-F2 | TMP1-R2 |  | 154 | TGGTTGTGATGGAGGTATT | 54.2 | AGATACAGATACGTTGGG | 52.7 |
| Solyc02g079500.4.1 | TMP1 | TMP1-F3 | TMP1-R3 |  | 153 | GTTGTGGACAGTGCTATT | 54.5 | GTTGGGTGGTGAGTTTTG | 55.4 |

Supplementary Table 5 Expression level comparison between RNA-Seq and qPCR (*C*Lso haplotype B vs. negative).

UID is unique identifier of gene in the tomato ITAG4.1 reference transcriptome. Name is unofficial abbreviation of UID, used only in primer design. Log2FoldChange is equivalent to -ΔΔCt in qPCR analysis. In qPCR analysis, ACT51 was used for internal control.

| **UID** | **Name** | **qPCR** | | |  | **RNA-Seq** |
| --- | --- | --- | --- | --- | --- | --- |
|  |  | **Sample (ΔCt)** | **Control (ΔCt)** | **Log2 Fold Change (-ΔΔCt)** |  | **Log2 Fold Change** |
| Solyc05g007070.2.1 | AA3 | 2.85 | 6.36 | 3.51 |  | 2.17 |
| Solyc08g068330.4.1 | AAC | 5.45 | 8.33 | 2.88 |  | 1.77 |
| Solyc11g005330.2.1 | ACT51 | 0.00 | 0.00 | 0.00 |  | 0.00 |
| Solyc02g069250.5.1 | ADS | 6.78 | 6.60 | -0.18 |  | -2.26 |
| Solyc09g009190.5.1 | AGBE22 | 5.20 | 5.50 | 0.30 |  | -1.08 |
| Solyc01g079790.5.1 | AGPL3 | 10.68 | 7.26 | -3.42 |  | -1.89 |
| Solyc02g080540.1.1 | ASGC | 5.03 | 2.44 | -2.59 |  | -1.58 |
| Solyc06g065990.1.1 | ASSB | 14.96 | 13.55 | -1.42 |  | -1.88 |
| Solyc09g091030.3.1 | BAM1 | 1.86 | 2.79 | 0.93 |  | 1.37 |
| Solyc02g069690.1.1 | BBE15 | 8.67 | 7.26 | -1.41 |  | -2.23 |
| Solyc09g065540.3.1 | BCC | 3.53 | 4.29 | 0.75 |  | 2.24 |
| Solyc03g119080.4.1 | BMP | 7.59 | 3.91 | -3.68 |  | -2.70 |
| Solyc07g047850.3.1 | CAB4 | 1.05 | -1.40 | -2.45 |  | -1.54 |
| Solyc03g116910.3.1 | CCR2 | 6.02 | 7.30 | 1.27 |  | -1.67 |
| Solyc08g082250.3.1 | CEL8 | 6.82 | 3.04 | -3.77 |  | -2.21 |
| Solyc01g006300.3.1 | CEVI1 | 4.06 | 2.77 | -1.30 |  | -1.78 |
| Solyc01g008110.5.1 | CPY51 | 3.56 | 2.13 | -1.43 |  | -2.43 |
| Solyc02g085020.4.1 | DDR | 6.14 | 10.66 | 4.52 |  | -2.31 |
| Solyc02g069490.4.1 | DSR | 6.60 | 4.45 | -2.16 |  | -2.38 |
| Solyc10g055800.2.1 | EC4 | 0.77 | 3.97 | 3.21 |  | 5.75 |
| Solyc05g005080.3.1 | EG25 | 6.72 | 1.82 | -4.89 |  | -2.19 |
| Solyc02g083810.4.1 | FNR | 2.92 | 0.35 | -2.57 |  | -1.37 |
| Solyc07g043310.3.1 | GAT1 | 0.36 | 3.00 | 2.64 |  | 3.12 |
| Solyc08g083320.4.1 | GBSS1 | 3.64 | 1.90 | -1.74 |  | -1.67 |
| Solyc07g056140.3.1 | GPA | 3.16 | 2.03 | -1.12 |  | -2.17 |
| Solyc02g089440.3.1 | GTF | 5.82 | 4.06 | -1.76 |  | -1.05 |
| Solyc04g015270.3.1 | GTF4 | 5.66 | 4.20 | -1.46 |  | -1.05 |
| Solyc06g083310.3.1 | GTF8 | 3.65 | 2.39 | -1.26 |  | -1.15 |
| Solyc04g081400.3.1 | HXK4 | 4.20 | 4.24 | 0.04 |  | -0.99 |
| Solyc11g069180.2.1 | ICD | 3.20 | 5.19 | 1.98 |  | 2.16 |
| Solyc04g078460.3.1 | IPA2 | 7.05 | 5.47 | -1.58 |  | -4.87 |
| Solyc12g006530.2.1 | ITTS1 | 0.96 | 4.70 | 3.75 |  | 2.24 |
| Solyc08g080040.4.1 | LDX2 | 5.33 | 8.32 | 3.00 |  | -2.27 |
| Solyc06g005750.3.1 | MM22 | 6.75 | 5.05 | -1.70 |  | -2.42 |
| Solyc09g007890.1.1 | PAL2 | 10.84 | 10.21 | -0.63 |  | -2.20 |
| Solyc06g084050.4.1 | PIIRCW | 10.84 | 5.94 | -4.89 |  | -2.00 |
| Solyc06g082940.3.1 | PIRC6 | 4.17 | 0.26 | -3.91 |  | -1.73 |
| Solyc08g074683.1.1 | PPOB | 10.47 | 4.12 | -6.35 |  | -3.50 |
| Solyc08g074620.3.1 | PPOE | 9.51 | 3.23 | -6.28 |  | -3.52 |
| Solyc08g074630.2.1 | PPOF | 9.08 | 4.34 | -4.73 |  | -3.70 |
| Solyc04g071900.5.1 | PSE12 | 5.06 | 3.69 | -1.38 |  | 4.01 |
| Solyc07g052510.4.1 | PSE3 | NA | 2.83 | NA |  | -2.01 |
| Solyc02g080530.3.1 | PSE42 | 2.22 | -0.41 | -2.63 |  | -1.03 |
| Solyc06g076630.3.1 | PSE47 | 7.59 | 5.44 | -2.15 |  | -1.78 |
| Solyc02g087190.1.1 | PSE63 | 7.04 | 3.65 | -3.39 |  | -1.95 |
| Solyc02g083490.3.1 | PSE64 | NA | 5.87 | NA |  | -2.11 |
| Solyc10g044520.3.1 | SEND33 | 0.10 | -2.20 | -2.30 |  | -1.08 |
| Solyc04g077440.4.1 | SM | 4.01 | 3.52 | -0.49 |  | -1.32 |
| Solyc02g079500.4.1 | TMP1 | 2.41 | 3.11 | 0.69 |  | 1.81 |
